# Elderly vulnerability to temperature-related mortality risks in China

**DOI:** 10.1126/sciadv.ado5499

**Published:** 2025-02-05

**Authors:** Xin Yao, Ying Qu, Ashok K. Mishra, Michael E. Mann, Liqiang Zhang, Chen Bai, Mengting Li, Jintai Lin, Jing Wei, Qiwei Yu, Ruiqiang Ding, Yuebin Wang, Lei Zhang, Jing Yang, Junpei Tao, Suhong Liu, Qihao Wang

**Affiliations:** ^1^ Faculty of Geographical Science, Beijing Normal University, Beijing 100875, China.; ^2^ Glenn Department of Civil Engineering, Clemson University, Clemson, SC, USA.; ^3^ Department of Earth and Environmental Science, University of Pennsylvania, Philadelphia, PA, USA.; ^4^ School of Labor and Human Resources, Renmin University of China, Beijing, 100872, China.; ^5^ Department of Atmospheric and Oceanic Sciences, School of Physics, Peking University, Beijing 100871, China.; ^6^ Institute of Carbon Neutrality, Peking University, Beijing, China.; ^7^ Department of Atmospheric and Oceanic Science, Earth System Science Interdisciplinary Center, University of Maryland, College Park, MD, USA.

## Abstract

The elderly face elevated mortality risk due to rising temperature. Previous assessments of temperature-related mortality, however, lack a comprehensive analysis of distinct impacts of temperature change across different timescales and characteristics. Using a longitudinal survey of 27,233 elderly Chinese citizens from 2005 to 2018, we establish connections between rising temperatures, temperature variability, and extreme heat with increased mortality risk, assessed through four annual metrics that combine temperature and humidity. The intensity and prolonged duration of extreme heat are found to have the greatest impact on mortality risk. Furthermore, by identifying heterogeneous impacts based on location, sex, age, obesity, income, and diet, we reveal the pathways through which temperature metrics are likely to influence mortality risk. Our study highlights the compound effects of rising temperatures for elderly populations, and it could be expanded to other countries and regions experiencing similar challenges due to an aging population experiencing warming conditions.

## Teaser

Rising temperatures, temperature variability, and extreme heat are linked to increased mortality risk of elderly Chinese citizens.

## INTRODUCTION

Recent climate extremes have shattered longstanding records by considerable margins, and such unprecedented extremes result in substantial impacts that were not observed during historical periods (*1*), posing a substantial threat to human health. The most immediate consequence of a shifting global climate on human health is evident in the gradual rise of the global average temperature, accompanied by increased intensity and prolonged duration of heat extremes and humidity (*2*, *3*). While average and extreme temperatures are expected to increase in the near future (*4*), the impact of temperature change on mortality in natural populations remains unclear (*5*–*8*). This emphasizes the necessity for a more profound understanding of the direct impacts of climate change on human health (*9*).

Previous studies investigated the temperature and mortality relationship using daily mean temperature (*10*–*13*), non-optimum ambient temperature (*14*), non-optimum ambient temperature at lagged times (*15*), cold and hot temperatures (*16*), average monthly temperature (*17*), minimum mortality temperature (*18*), heat index (*19*), hot night excess (*20*), and excess heat factor (EHF) (*21*–*23*). In addition to these temperature metrics, temperature variability might play a substantial role in elevating mortality risk (*24*). Many of these studies have used daily temperature in correlation with all-cause mortality over a shorter observational period (e.g., a few weeks). However, they overlook the long-term (e.g., annual) effects of elevated temperature and temperature variability in the analysis of temperature-mortality relationships (*24*). To date, there has been limited exploration into the impacts of changes in various temperature metrics and their intricate interactions. This limitation poses a challenge to gaining a comprehensive understanding of the health consequences of climate change, particularly in light of the concurrent increase in average temperature, temperature variability, and the frequency and intensity of extreme temperature events (*25*, *26*). In addition, it is essential to recognize that temperature and temperature change exhibit pronounced variations across regions (*27*), making it unclear how these regional differences will affect mortality.

In particular, the potential health effects of climate change on older adults present a noteworthy concern (*28*). Their health is particularly vulnerable to temperature changes (*28*), which may be underestimated due to the absence of dedicated datasets (*29*). To precisely target public health interventions, conducting comprehensive longitudinal surveys involving older adults in diverse temperature settings is essential for assessing the associated health effects (*15*, *29*). China is now home to the largest population of older people (aged 65 and above), constituting 25.6% of the global older population in 2020 (*30*–*32*). The health burden associated with climate change in China is further compounded by demographic shifts (*33*). Furthermore, the risk of temperature-related mortality in developing countries including China where individuals face substantial vulnerabilities and climate change impacts (*34*, *35*) is less investigated compared to the risk in developed countries (*29*). Examining the influences of temperature change on the mortality risk of older adults in China serves as a representative case study for elucidating the temperature-mortality relationship.

Here, we explore the relationship between temperature change and mortality risk of Chinese older adults at different timescales and characteristics based on the Chinese Longitudinal Healthy Longevity Survey (CLHLS) from 2005 to 2018. The common definition of the older adults (aged 65 and above) corresponds to a retirement age of 65 in academic and policy discourse (*28*). The CLHLS is the most comprehensive longitudinal health dataset available for older adults in China. It consists of 27,233 individuals who are at least 65 years old from 917 counties in 23 provinces across China (the total populations of these counties contribute about 85% of the Chinese population). The temperature metrics used in previous studies are derived from four fundamental categories of daily temperatures (fig. S1), which capture temperature conditions at different times throughout the day. For example, hot night excess is calculated from daily minimal temperature (*20*), non-optimum ambient temperature is generated from time series of daily mean temperature (*14*, *15*), and EHF considers hot days or hot nights based on the average daily temperature (*21*–*23*). However, elevated temperatures have a more pronounced impact when compounded by high atmospheric relative humidity, slowing heat dissipation from the human body and increasing heat stress (*36*). Here, in addition to four types of daily temperature including daytime maximum temperature, nighttime minimum temperature, daily mean temperature, and average daily (i.e., the average of daytime maximum and nighttime minimum) temperature, we develop four respective daily indices by coupling these temperatures with relative humidity (Materials and Methods). Then, four annual temperature metrics (i.e., annual mean temperature index, day-to-day temperature index variability, annual mean EHF, and the number of days with EHF > 0; Materials and Methods) are derived from each of the eight daily temperature metrics (four temperatures plus four indices) to reflect the long-term trends and cumulative impacts of temperature change throughout the year (fig. S1).

Leveraging the four annual temperature metrics over 2004–2018 and the CLHLS data over 2005–2018, we apply a suite of high-dimensional fixed effects models (*37*, *38*) to quantify the linear and potential nonlinear temperature-mortality associations for the annual temperature metrics. Each of these models includes two sets of high-dimensional fixed effects: (i) year-specific shocks of each province using province-by-year fixed effects, which capture factors such as local medical policies, macroeconomy, and basic welfare facilities; and (ii) the month-specific shocks of each province using the province-by-month fixed effects (e.g., seasonality of diseases and seasonal variation in lifestyle habits in older adults). This approach allows us to account for unobserved differences between provinces, contemporaneous provincial shocks, and province-specific time trends (such as multiyear socioeconomic and demographic trends), thereby enhancing the precise identification of the relationship between temperature change and mortality risk. Furthermore, there are large interregional and urban-rural differences in climate, geography, built environment, social structure, and adaptive capacity, which may exacerbate health disparities among vulnerable populations (*39*). We thus investigate the potential roles of socioeconomic, geographic, and demographic factors in the temperature-mortality association, including household location, sex, age, body mass index (BMI), wealth, urban-rural difference, and diet. Our results provide a comprehensive assessment of the mortality burden associated with temperature changes and confirm the heightened mortality risk faced by older adults due to temperature fluctuations. This effort provides valuable resources for guiding policy decisions related to climate change and its implications for human health in a warming world.

## RESULTS

### Independent influences of the temperature metrics

We assess the effects of four different temperature metrics at the annual timescale on the mortality risk of older adults in China, by using multivariate linear panel regression with two fixed effects (province-by-year fixed effects and province-by-month fixed effects; based on the specification of model 1; Eq. 8 in Materials and Methods). A total of eight sets of linear regression, each with four annual temperature metrics derived from one of the eight daily temperature metrics, are conducted. Consistent with previous studies (*19*, *21*–*24*), our experiments show that increases in annual mean and extreme temperatures are associated with elevated mortality risk (fig. S4, A, C, and D). This suggests that the rise in long-term ambient temperature, increased intensity, and prolonged duration of extreme heat experienced by older adults enhance health risk. We also find a positive linear relationship between day-to-day temperature index variability and mortality risk (fig. S4B). The linear effects of the four annual temperature metrics are all statistically significant when they are simultaneously included in the regression (table S2), implying that each annual temperature metric constitutes additional effects distinct from others. In particular, the temperature variability represents an effect not previously identified in the temperature-mortality responses. Older adults living in environments with the same annual mean temperature and extreme heat events may, therefore, experience different overall impacts due to differences in temperature variability.

Daily temperature metrics represent temperatures at various times of day, such as daytime and nighttime. Because of high correlation and collinearity among these metrics derived from different daily measures (tables S4 and S5), we could not include all of them in a single regression analysis, except for those based on daily maximum and minimum temperatures. To identify the most effective daily temperature metrics for assessing the impact of annual temperature metrics on mortality risk, we calculated the Akaike information criterion (AIC) and Bayesian information criterion (BIC) and evaluated precision, recall, and F1 score through fivefold cross-validation for each annual metric-based regression model that included the four annual temperature metrics (Supplementary Text S4 and table S7). The temperature-humidity indices, considering the integration of temperature with humidity, often has a better explanatory power than temperature alone in the assessment of the temperature-mortality relationship (table S7). In all the regression models, the model including the annual mean temperature index calculated from the daily maximum temperature index, the day-to-day temperature index variability calculated from the daily mean temperature index, and the annual mean EHF and the days with EHF > 0 calculated from the average daily temperature index exhibit the lowest AIC and BIC (table S7). Therefore, we select the linear regression model containing this set of variables as the baseline regression model for subsequent analyses.

Given that extreme heat events usually occur during the warm season and that annual temperature variables may introduce unidentified confounding factors, we also investigate the relationship between mortality risk and four warm season temperature metrics derived exclusively from warm season climate data. Here, the warm season is defined as the six consecutive months with the highest monthly average temperatures in a year (often in April to September; Materials and Methods). The mortality effects of warm season day-to-day temperature index variability, mean EHF, and number of days with EHF > 0 are similar to those of the corresponding annual temperature metrics (figs. S4, B to D, and S5, B to D, and tables S2 and S3). However, the mortality effect of the warm season mean temperature index is lower than that of the annual mean temperature index (figs. S4A and S5A and tables S2 and S3), indicating an important association between mean temperature changes during the cold season and mortality risk of older adults.

To evaluate the appropriateness of parameter settings and the robustness of the effects of the four annual temperature metrics, we modified the baseline model and performed several comparative tests. These tests included (i) evaluating the appropriateness of using a linear probability model (Supplementary Text S5, fig. S6, and tables S8 and S9); (ii) examining the validity of province-by-year and province-by-month fixed effects (Supplementary Text S6, fig. S7, and table S10); (iii) assessing the adequacy of SE adjustments (Supplementary Text S7 and table S11); (iv) testing the robustness of the effects of the four annual temperature metrics (Supplementary Text S8, fig. S8, and table S12); and (v) evaluating the linear regression of control variables with mortality risk (Supplementary Text S9, fig. S9, and table S13). The results from all these tests align with the findings from the baseline model.

### Temperature-mortality associations

**Fig. 1.**
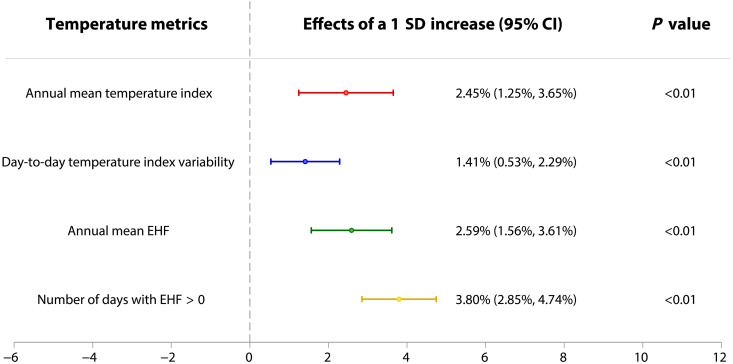
Linear effects of four annual temperature metrics on mortality risk of older adults. Points and lines represent mortality effect estimates and their 95% CIs for a 1 SD increase in each of annual temperature metrics for the baseline model. The baseline regression includes province-by-year fixed effects and province-by-month fixed effects, and the SEs are clustered at the county level. More details are presented in table S14.

In the baseline regression model, elevated mortality risk of older adults is linearly associated with an increase in each of the four annual temperature metrics (Fig. 1 and table S14). Since the magnitude of each annual temperature metric is different, the regression coefficients do not provide comparable estimates of the effects. Therefore, we multiply the regression coefficient by the sample SD for each annual temperature metric. The results show that annual mean EHF and number of days with EHF > 0 have stronger association with mortality risk than the other two annual temperature metrics, on the basis of per extra 1 SD change. As shown in Fig. 1, per extra 1 SD of the two metrics increases mortality risk by 2.59% [95% confidence interval (CI): 1.56 to 3.61%] and 3.80% (95% CI: 2.85 to 4.74%), respectively. This indicates that, among the changes in the four annual temperature metrics, the intensification of extreme heat events is the dominant factor contributing to the increase in mortality risk.

**Fig. 2.**
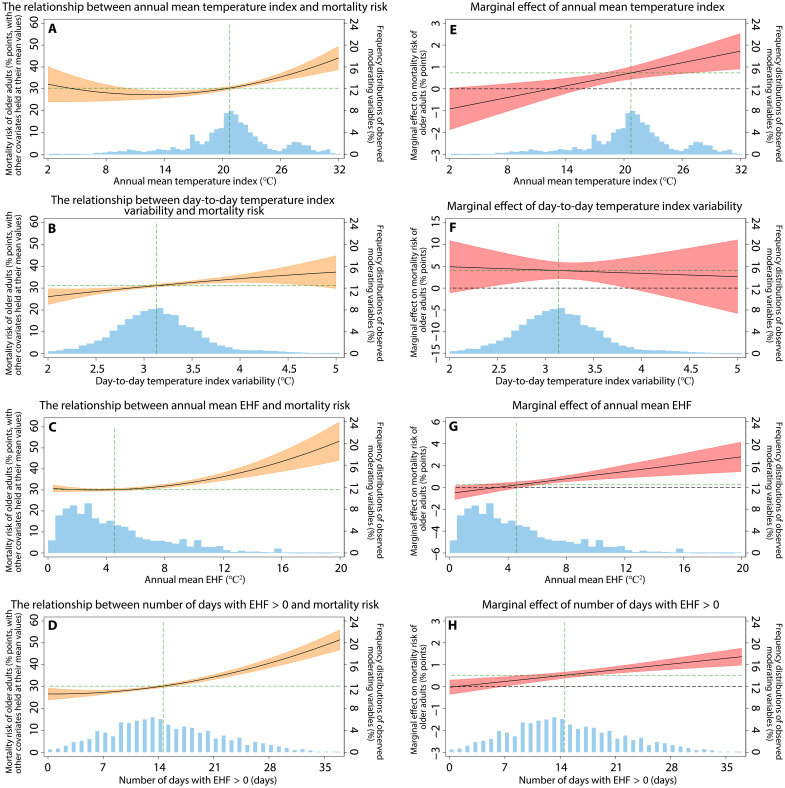
Nonlinear effects of four annual temperature metrics on mortality risk of older adults. (**A** to **D**) The relationship between each of four annual temperature metrics and overall mortality risk of older adults, with other covariates held at the sample means. (**E** to **H**) The marginal effect of each annual temperature metric on mortality risk, representing the mortality impact of a small change in the respective annual metric. The 95% CIs are shown in orange or red shades. The green dashed line represents the mortality risk or marginal effect at the sample mean of the respective annual temperature metric. The frequency distributions of observed moderating variables are shown as blue histograms. The regression supporting these results is based on the specification of model 2. More details are presented in column 1 in table S16.

To investigate the potential nonlinear effects and interactions of the annual temperature metrics, we included the quadratic terms for the annual metrics in models 2 and 3 (Eqs. 9 and 10; see Materials and Methods and table S16) and the interaction terms in model 4 (Eq. 11; see Materials and Methods and table S17) in the additional regression analyses. In the four temperature metrics, the quadratic terms of the annual mean temperature index, annual mean EHF, and the number of days with EHF > 0 are statistically significant (table S16). Specifically, the marginal effect of the annual mean temperature index on the mortality risk for older adults is smaller at lower values of the metric and increases progressively as the metric rises (Fig. 2E), indicating that the long-term warming trend attributed to climate change has a greater impact on population health in hot and humid regions. Similarly, the annual mean EHF and the number of days with EHF > 0 exert stronger effects on the mortality risk of older adults as these metrics increase (Fig. 2, C, D, G, and H). These quadratic effects imply spatial heterogeneity depending on local temperature and humidity conditions. The quadratic effect of day-to-day temperature index variability is statistically insignificant (model 2; column 1 in table S16). However, the linear effect of day-to-day temperature index variability in the baseline model (Fig. 1 and table S14) is statistically significant, as is the linear effect when it is regressed alongside the quadratic terms of the other three annual temperature metrics (model 3; Eq. 10 in Materials and Methods; column 2 in table S16). This indicates that day-to-day temperature index variability has an adverse effect on mortality risk, but its spatial heterogeneity is relatively weak (Fig. 2, B and F). In addition, the interaction effects between annual temperature metrics are not statistically significant (table S17).

The traditional EHF uses the 95th percentile of the average daily temperature within a climatic period as a threshold to capture extreme heat events (Materials and Methods). For sensitivity tests, we replace the threshold with the 90th percentile and the 99th percentile to further investigate the impacts of extreme heat events with varying intensities on mortality risk (figs. S10 and S11 and table S18). The linear and quadratic effects of number of days with EHF > 0 are similar for these three thresholds (figs. S10B and S11 and table S18). Notably, the linear effect of the annual mean EHF increases with higher thresholds (fig. S10A; columns 1 to 3 in table S18), while the quadratic effect is not statistically significant at the 90th percentile but becomes significant at the 95th and 99th percentiles (fig. S11, A to F; columns 4 to 6 in table S18). This suggests that more intense extreme heat events have a greater impact on the mortality risk of older adults and that the spatial heterogeneity of this impact also increases with greater intensity.

Each of the annual temperature metrics has a statistically significant effect on mortality risk in the baseline regression model, indicating that the temperature metrics have independent and additive effects. To assess the independence of these metrics, first, we progressively remove a specific annual temperature metric from the model (rows 2 to 5 in fig. S12 and table S19), and second, we evaluate each annual temperature metrics individually (rows 6 to 9 in fig. S12 and table S20). The effect of the annual mean temperature index on mortality risk increases as day-to-day temperature index variability or annual mean EHF is excluded from the model; and removing the annual mean temperature index enhances the effect of day-to-day temperature index variability and annual mean EHF [see fig. S12 (A to C) and table S19]. This indicates some level of interdependence among these annual temperature metrics, as they exert similar effects on mortality risk. However, the competition among the three metrics is minor, given the weak collinearity among them (table S15). Conversely, the effect of annual mean temperature index decreases as the number of days with EHF > 0 is excluded, and the regression model without annual mean temperature index also leads to a decrease in the effect of the number of days with EHF > 0 (fig. S12, A and D, and table S19). Thus, they are complementary metrics that assist one another in identifying their individual effects on mortality risk. The effect of a 1 SD change in a specific annual temperature metric, when assessed individually, is greater than the effect of the same change when all four metrics are analyzed together, yet it is less than the total effect of 1 SD changes across all four metrics (fig. S12 and table S20). This indicates that analyzing the relationship between a single temperature metric and mortality risk may present two potential issues: (i) overestimating the impact of that particular metric on mortality risk due to inadequate control for variations in other temperature characteristics, and (ii) underestimating the overall effect of temperature changes from multiple metrics by failing to account for these variations.

### Cumulative change in mortality risk linked to temperature

**Fig. 3.**
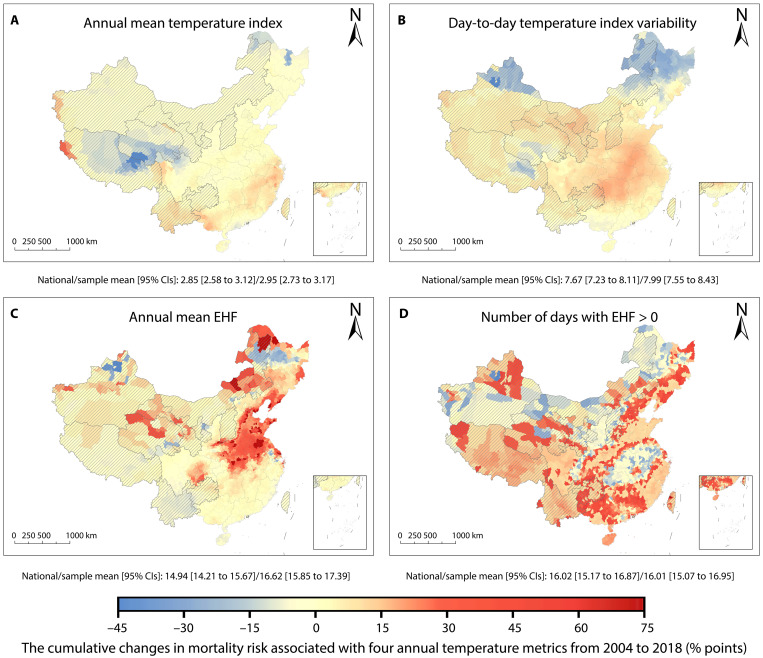
County-level estimates of the cumulative changes in mortality risk linked to temperature. The cumulative change in mortality risk attributed to variations in (**A**) annual mean temperature index, (**B**) day-to-day temperature index variability, (**C**) annual mean EHF, and (**D**) number of days with EHF > 0 from 2004 to 2018. For each county, the estimates are derived by summing the annual differences in mortality risk associated with the four annual temperature metrics over 2005–2018 relative to 2004, based on county-level climate data and mortality effect estimates from model 3. The hatching indicates the regions without CLHLS data. The national mean and CLHLS sample mean weighted by the population of impacts of each county are given in each panel.

On the basis of the mortality effect estimates derived from the regression model with quadratic terms (model 3; Eq. 10) and climate data from 2004 to 2018, we assess the cumulative changes in mortality risk attributed to the variations in the four annual temperature metrics. For each county, we separately calculate the annual differences in mortality risk associated with the four annual temperature metrics from 2005 to 2018, relative to 2004. We then sum these differences to represent the cumulative changes in mortality risk linked to temperature change (Materials and Methods). For the 11 provinces without CLHLS data (hatched in Fig. 3), we calculate the cumulative changes by using the temperature-mortality associations identified in other provinces.

The cumulative changes in mortality risk over 2005–2018 associated with the annual mean EHF and the number of days with EHF > 0 are more pronounced than the mortality risk changes linked to the other two metrics (Fig. 3). Among these, changes in the number of days with EHF > 0 have contributed to increased mortality risk in most counties nationwide, cumulatively raising the national mortality risk by 16.02% (95% CI: 15.17 to 16.87%). This indicates that, on average, the annual mortality risk of older adults in China during 2005–2018 is 1.14% (16.02%/14) higher than in 2004. The impact of the changes in the number of days with EHF > 0 is particularly great in southern China, with the average annual increases reaching 5.36% (75%/14) in certain regions. Conversely, the cumulative impacts of the annual mean EHF have been substantially greater in northern and northeastern China (Fig. 3C) due to larger marginal effects resulting from historically higher mean EHF levels in these regions. The cumulative changes in mortality risk linked to the annual mean temperature index align with the spatial pattern of China’s annual average temperature index, showing greater changes in the southeast and smaller changes in the northwest China (Fig. 3A). The cumulative impacts of the day-to-day temperature index variability on mortality risk is primarily concentrated in the central, eastern, and southern China (Fig. 3B).

### Impact heterogeneities across demographics, economic status, and diets

Older adults with different demographics, economic status, and diets might have different temperature-mortality responses. By adjusting the linear (model 5, Eq. 12) and quadratic (model 6, Eq. 13) models, we investigate the moderating effects of these factors on the mortality impacts of the four annual temperature metrics.

#### Heterogeneity across demographics

**Fig. 4.**
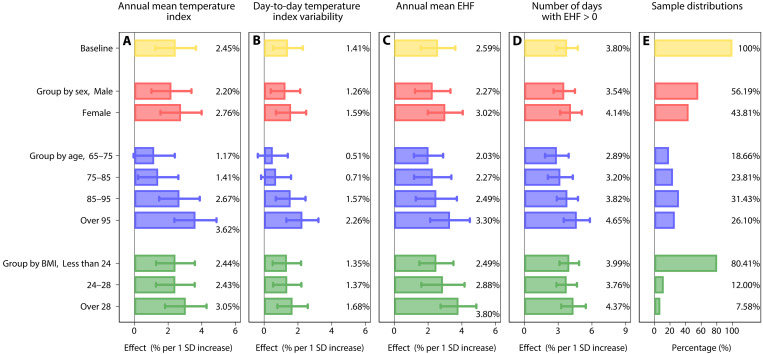
Heterogeneities by demographics in the mortality effects of four annual temperature metrics. The effects of a 1 SD increase in (**A**) annual mean temperature index, (**B**) day-to-day temperature index variability, (**C**) annual mean EHF, and (**D**) number of days with EHF > 0 on mortality risk across demographics subgroups. (**E**) Sample proportions among different subgroups. Dots and lines show mortality effect estimates and their 95% CIs. Each regression is computed by using a fixed-effects model with interaction terms based on the specification of model 5 (red bars include an interaction of sex factor variable with each of the four annual temperature metrics; blue bars include an interaction of age factor variable with each of the metrics; green bars include an interaction of the BMI factor variable with each of the metrics) and includes province-by-year and province-by-month fixed effects. SEs are clustered at the county level. More details are presented in tables S21 to S24.

We first assess the heterogeneity in the mortality effects by sex, age, and obesity subgroups (tables S21 to S24). Statistically significant differences exist across these subgroups in the mortality impacts of the four annual temperature metrics (Fig. 4).

The mortality effects of the four temperature metrics on older women are significantly higher than on older men (Fig. 4, A to D; column 1 in tables S21 to S24), and the effect differences become more pronounced in the regions with higher annual mean temperature index and annual mean EHF (fig. S13, A and B). The mortality effects for the annual mean temperature index and day-to-day temperature index variability increase with age (Fig. 4, A and B; column 2 in tables S21 and S22). The effects of 1 SD increase in the two metrics for the subgroup aged > 95 are nearly three to four times as large as those of the subgroups aged 65 to 75 and 75 to 85. For the age subgroups 65 to 75, 75 to 85, and 85 to 95 years, there is no statistically significant difference in the impacts of the annual mean EHF and number of days with EHF > 0 on mortality risk (column 2 in tables S23 and S24). However, in the age subgroup over 95 years, the impacts of these two metrics on mortality risk is significantly greater compared to the younger age subgroups (Fig. 4, C and D; column 2 in tables S23 and S24). In addition, the BMI strongly modulates the effects of the annual mean temperature index and day-to-day temperature index variability, with a greater effect on obese older adults (BMI > 28; Fig. 4, A and B; column 3 in tables S21 and S22).

#### Heterogeneity across economic status

**Fig. 5.**
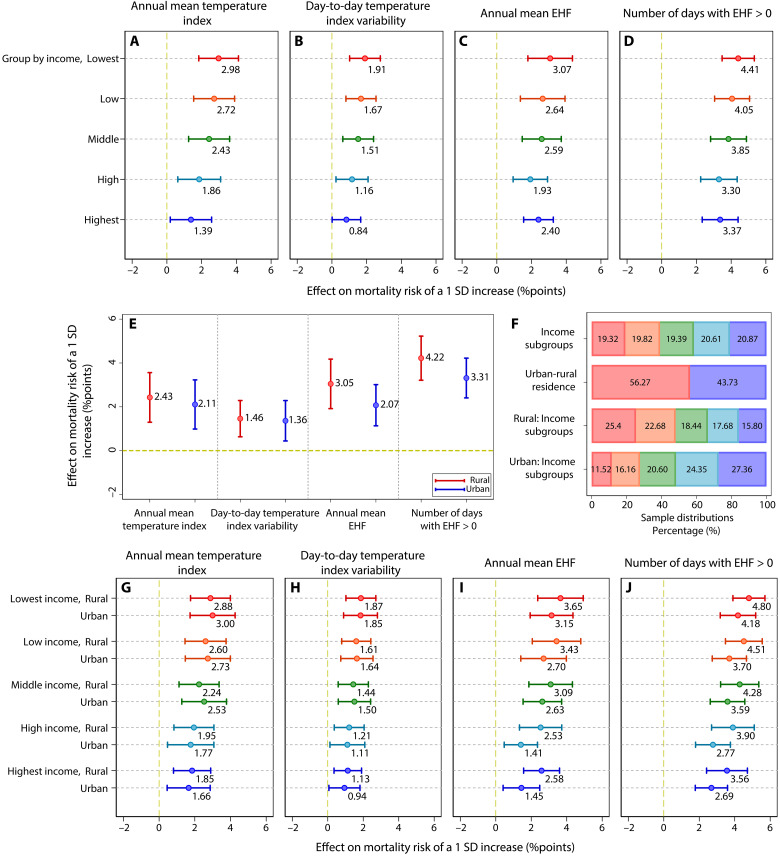
Heterogeneities of mortality effects across incomes and urban-rural residences. (**A** to **D**) The effects of a 1 SD. increase in the four annual temperature metrics on mortality risk across income subgroups. (**E**) The effects of a 1 SD increase in the four annual temperature metrics on the mortality risk with different urban-rural residences. (**F**) Sample proportions for different subgroups. The same colors in (F) and other panels represent the same subgroups. For example, the red color in the second row of (F) and the red color in (E) both indicate rural residences, while blue represents urban residences. (**G** to **J**) The effects of a 1 SD increase in the four annual temperature metrics on the mortality risk with different incomes and urban-rural residence. Dots and lines show effect estimates and their 95% CIs. Each regression is computed by using a fixed-effects model with interaction terms based on the specification of model 5 and includes province-by-year and province-by-month fixed effects. SEs are clustered at the county level. More details are presented in tables S25 to S28.

We further explore how the temperature-mortality relationship might be affected by household per capita annual income (Fig. 5, A to D; column 1 in tables S25 to S28). The mortality effects of all annual temperature metrics except annual mean EHF show statistically significant differences across five subgroups separated by household income quintiles (*P* < 0.01; column 1 in tables S25, S26, and S28); that is, the mortality risk decreases as the affluence increases (Fig. 5, A, B, and D). The marginal effects of annual mean temperature index and number of days with EHF > 0 are linear functions of their respective metrics, but the increasing rate of the marginal effects becomes smaller with the affluence increase (fig. S14), suggesting that higher income directly shields against the effects of increases in the two metrics to some extent. The mortality effects of annual mean EHF appear to be similar for low- and middle-income groups (Fig. 5C). The high-income subgroup is less affected by the annual mean EHF (Fig. 5C; column 1 in table S27), but the difference in impact between the highest- and lowest-income subgroups is not statistically significant (*P* > 0.1; column 1 in table S27). Thus, the adaptability to EHF-related effects due to higher economic levels is mainly reflected in the resistance against the duration of extreme heat events rather than the severity of extreme heat events.

Compared with those living in rural areas, older adults in urban areas are less affected by the four temperature metrics (Fig. 5E; column 2 in tables S25 to S28). We further investigate the heterogeneities through the lens of intersectionality by examining an interaction term of urban-rural residence and income. The effects of annual mean EHF and number of days with EHF > 0 on mortality risk show urban-rural differences across all income groups (Fig. 5, I and J, and fig. S15, G to R; column 3 in tables S27 and S28). However, the disparities in the mortality effects of annual mean temperature index and day-to-day temperature index variability are only observed in high- and highest-income subgroups (Fig. 5, G and H, and fig. S15, B to F; column 3 in tables S25 and S26). This indicates that the resilience against the long-term trend of temperature warming and temperature variability in urban areas mainly exists among older adults with higher-income households.

#### Diet heterogeneity

**Fig. 6.**
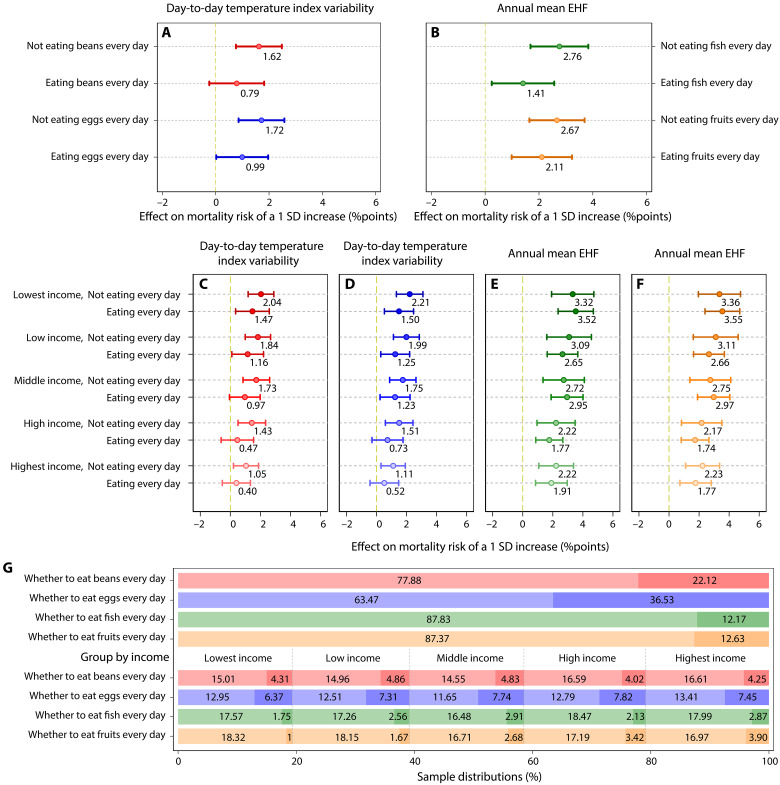
Heterogeneities across diets in mortality effects of day-to-day temperature index variability and annual mean EHF. (**A**) The effects of a 1 SD increase in the day-to-day temperature index variability on mortality risk across diet subgroups. (**B**) The effects of a 1 SD increase in the annual mean EHF on mortality risk across diet subgroups. (**C** and **D**) The effects of a 1 SD increase in the day-to-day temperature index variability on the mortality risk with different incomes and diet choice [beans in (C) and eggs in (D)]. (**E** and **F**) The effects of a 1 SD increase in the annual mean EHF on the mortality risk with different incomes and diet choice [fish in (E) and fruits in (F)]. (**G**) Sample proportions for different subgroups, with the colors corresponding to those in the other panels representing the same subgroups. Dark colors represent daily consumption of the specific food, while light colors indicate nondaily consumption. Dots and lines show effect estimates and their 95% CIs. Each regression is computed by using a fixed-effects model with interaction terms based on the specification of model 5 and includes province-by-year and province-by-month fixed effects. SEs are clustered at the county level. More details are presented in tables S29 to S36.

Diet choice is important to health (*40*–*42*), but it is not clear whether and how the mortality risk of temperature change is modulated by diet. We categorize older adults into different subgroups according to their staple food and whether they regularly consume vegetables, fruits, pork, fish, eggs, beans, pickles, tea, and garlic on a daily basis (Materials and Methods). As listed in tables S29 to S36, we find that the mortality effect of day-to-day temperature index variability is stronger on older adults who consume beans and eggs not everyday, compared to those who do (Fig. 6A). Consuming fish and fruits daily is associated with lower effects for the annual mean EHF (Fig. 6B).

Recognizing that the moderating effects of diet on the relationship between day-to-day temperature index variability, annual mean EHF, and mortality risk may be shaped by household income differences, we interact each of the four dietary binary variables (indicating daily consumption of beans, eggs, fish, or fruits) with five household income subgroups. This creates four factor variables, each containing 10 values. We then combine these interaction variables with the day-to-day temperature index variability and annual mean EHF to assess for heterogeneity (Fig. 6, C to F). Whether older adults consume beans or eggs daily is not related to income levels (Fig. 6G), and their moderating effects on the mortality impacts of day-to-day temperature index variability are highly significant across all income groups (Fig. 6, C and D). However, the proportion of older adults who consume fish or fruits daily increases with income (Fig. 6G), and the moderating effects on the mortality impacts of annual mean EHF do not appear in every income group (Fig. 6, E and F), suggesting that the moderating effect might be income dependent.

## DISCUSSION

In recent decades, heat waves worldwide have exhibited a surge in intensity, frequency, and duration. Concurrently, China is experiencing climate warming at an unprecedented rate (*43*, *44*). Assessing the mortality effects of temperature change based on longer periods of longitudinal datasets is crucial for a comprehensive understanding of the temperature-mortality relationship among older populations. We calculate four annual temperature metrics from daily temperatures: the annual mean temperature index, day-to-day temperature index variability, annual mean EHF, and the number of days with EHF > 0. These metrics capture long-term temperature trends and variability, as well as the intensity and duration of extreme heat events. Notably, the integrated temperature-humidity index demonstrates greater explanatory power than temperature alone in assessing the temperature-mortality relationship. Our findings highlight the adverse effects of day-to-day temperature index variability on the mortality risk of older adults, which occur independently of long-term warming trends and extreme heat events.

Unlike the day-to-day temperature index variability, the marginal effects of the other three annual temperature metrics demonstrate regional heterogeneity due to local temperature conditions. The annual mean temperature index exerts a more substantial impact on mortality risk in hot and humid regions. Likewise, changes in the annual mean EHF and the number of days with EHF > 0 have more pronounced effects on older adults residing in regions with more severe and prolonged extreme heat events, respectively. Furthermore, the impact of the four annual temperature metrics on the mortality risk of older adults exhibits either competitive dynamics (e.g., annual mean temperature index versus day-to-day temperature index variability or annual mean EHF) or complementary and supportive patterns (e.g., annual mean temperature index versus the number of days with EHF > 0). Consequently, the association between mortality risk and each annual temperature metric, as well as the assessment of the overall impact of temperature changes on mortality risk, may be skewed if all four metrics are not considered simultaneously.

The cumulative changes in mortality risk attributed to the variations in four annual temperature metrics exhibit substantial spatial heterogeneity. The greatest cumulative impacts on mortality from 2005 to 2018, relative to 2004, were due to changes in the annual mean EHF and the number of days with EHF > 0. In addition, the impacts of annual mean temperature index and day-to-day temperature variability are more evident in southern China, whereas the effects of annual mean EHF are more pronounced in northern China. Therefore, formulating response policies tailored to the characteristics of each region remains a priority for mitigating the impact of climate change.

The mortality risk of older adults from climate warming depends on not only the level of exposure to temperature changes but also the vulnerability of populations (*45*). Our results indicate that the effects of the four temperature metrics on mortality risk of older adults are modulated by the socioeconomic status of households. Older adults with higher household incomes are less affected by temperature change. This is likely because affluent households have better living conditions, such as the use of air conditioning, cool roof materials, and winter heating facilities, which can better mitigate the impact of ambient temperature variability or extreme temperatures (*46*–*48*). Among high-income older adults, those living in urban areas are less affected by the long-term trend of temperature warming and temperature variability compared to those in rural areas.

Differences in sex, age, and obesity may amplify the impacts. The older adults participating in this study were born during the socioeconomically challenging times of the 1920s to 1940s, when food scarcity and nutritional imbalances in infancy, childhood, and youth likely adversely affected their health. Typically, female nutrition was poorer than that of males (*49*), which could contribute to a higher temperature-related risk for older women compared to men. In addition, the mortality impacts of the annual mean temperature index and day-to-day temperature index variability increase with age, likely because aging itself is a substantial risk factor for many fatal diseases (*50*). Older adults are more vulnerable to temperature change due to aging development, declining physical functions, particularly in thermoregulation, and an underlying accumulation of pathophysiological changes that contribute to mortality over time (*51*). In addition, the BMI strongly modulates the effects of the four annual temperature metrics on mortality risk, with a greater effect on obese older adults. Obesity is a well-known factor in aging (*50*), with physiological changes caused by obesity increasing older adults’ susceptibility to temperature fluctuations.

Older adults who consume beans and eggs daily are less susceptible to day-to-day temperature index variability. This may be related to the thermoregulatory mechanisms of older adults in response to environmental temperature changes, such as digestive burden, protein loss, and electrolyte imbalance (*52*–*55*). Compared to previous studies (*48*, *56*, *57*), our study sheds light on the temperature-mortality relationship by identifying diet as a moderator. Specifically, certain diets serve as a buffer, helping mitigate the mortality impact of temperature changes.

Additional research could address some of the limitations of this initial study. The CLHLS data only cover 23 provinces in China, and caution should be exercised in extrapolating our results to the remaining 11 provinces. The focus on all-cause mortality, moreover, hides the possible cause-specific impacts. Last, the lack of historical residential address information for the elderly adult samples hinders the assessment of the effect of population migration. However, given that all participants were aged 65 years and older, the probability of migration is relatively low.

Overall, our study provides a more comprehensive understanding of elderly mortality risk of from human-caused warming. Given the heightened sensitivity of older adults to changing temperature characteristics, there is a need for targeted mitigation measures in the places where the challenges of an aging population have been identified as particularly acute (*58*). Our basic methodology could potentially be extended to other countries and regions grappling with the increased compound risk exposure from an aging population and increasing temperatures.

## MATERIALS AND METHODS

### Climate data

The primary source of climate data is the European Centre for Medium-Range Weather Forecasts Reanalysis v5 (ERA5) (*59*). The ERA5 combines satellite and in situ observations with state-of-the-art assimilation and modeling techniques to provide estimates of climate variables with global coverage and at one-hourly and 0.25° × 0.25° resolution. We obtain daily mean temperature, daily maximum temperature, daily minimum temperature at 2 m above the surface, as well as daily total precipitation from 2004 to 2018. In addition, we use the EWEMBI (EarthH2Observe, WFDEI, and ERA-Interim data merged and bias-corrected for the Inter-Sectoral Impact Model Intercomparison Project) (*60*) reanalysis datasets and test the consistency of the assimilation and interpolation techniques applied by reanalyses to the observational data. The EWEMBI reanalysis data are obtained at a daily timescale and on a regular 0.5° × 0.5° grid for the years 2004–2016. All data are used to calculate temperature metrics at the county level.

### CLHLS data

Our survey samples are obtained from the CLHLS that is a prospective, longitudinal, population-based study of older adults in China. The survey began in 1998 and has been conducted every 2 to 3 years. It covers half of the cities and counties across 23 provinces (including provincial-level municipalities and autonomous regions) in China; these counties account for approximately 85% of the national total population. The CLHLS survey questionnaire consists of two types: the questionnaire for surviving respondents and the questionnaire for family members of deceased older adults. The survey for surviving respondents includes information about their demographic factors, socioeconomic status, self-reported health and quality of life, cognitive function, physical function, mental health, personality traits, lifestyle, care, disease treatment, and medical expenses. The survey for family members of deceased elderly includes information about the time and place of death. CLHLS was approved by the Biomedical Ethics Committee, Peking University, Beijing, China (IRB00001052-13074). Written informed consent was obtained from all participants.

We use all samples from the five waves CLHLS between 2005 and 2018 (wave 1: 2005; wave 2: 2008–2009; wave 3: 2011–2012; wave 4: 2014; wave 5: 2017–2018; fig. S2B), including 27,233 elderly respondents from 917 county-level administrative units. Of these respondents, 11,412 (41.91%) are female and 15,821 (58.09%) are male. Among them, 1108 older adults participated in five waves (0 of whom died); 2566 older adults participated in four waves (747 of whom died); 3288 participated in three waves (1940 of whom died); 5975 participated in two waves (3907 of whom died); and 14,296 participated in one wave (9274 of whom died). Last, we construct a panel dataset comprising 27,193 respondents and 51,914 observations with a follow-up of 13 years, of whom 15,868 respondents passed away during the follow-up.

Each of older adults is matched with temperature metrics in the county where he/she lives from 1 year before the start time (using the interview time as the start time for surviving respondents and the death time as the start time for deceased participants). During the data matching process, we reassign the county of some older adults due to the factors like county boundary changes (Supplementary Text S1). We also collect their socioeconomic status, health, dietary habits, medical conditions, and family information.

### Daily temperatures and indices

We choose daily mean temperature, daily maximum temperature, daily minimum temperature, and average daily temperature as four fundamental categories of daily temperatures, where the average daily temperature is the average of daily maximum temperature and daily minimum temperature. For each of the four daily temperatures, we calculate four corresponding daily temperature indices using the method similar to that calculating heat index by the US National Weather Service for (*61*). A brief description of the algorithm is provided in the Supplementary Text S2. The relative humidity in the daily temperature index is based on ERA5 daily maximum temperature and daily mean dew point temperature estimated by using the Magnus approximation (*62*).

$$\text{RH}=\frac{\text{exp}\left(\frac{17.625\times T_{d}}{243.04+T_{d}}\right)}{\text{exp}\left(\frac{17.625\times T_{\text{max}}}{243.04+T_{\text{max}}}\right)}\times 100$$ (1)

where $T_{d}$ and $T_{\text{max}}$ represent daily mean dew point temperature and daily maximum temperature in degrees Celsius. We also take these four daily temperature indices as four daily temperature metrics. Each of the eight daily temperature metrics is used to create a set of four distinct annual temperature metrics, capturing different characteristics of the daily metrics on an annual scale (fig. S1). Ultimately, the mortality risk of older adults was modeled eight times, with each model incorporating four annual temperature metrics based on one of the eight daily temperature metrics (Eq. 8; fig. S4 and table S2). In addition, we use only the warm season temperature data from the year before the date of survey or death to generate four different warm season temperature metrics for each of the eight daily temperature metrics. The warm season is defined as the six consecutive months with the highest average monthly temperatures in the year. These warm season temperature metrics are also used to fit eight models to compare their differences with the annual temperature metrics (Eq. 8; fig. S5 and table S3).

### Day-to-day temperature index variability

Similar to the previous study (*63*), we measure day-to-day temperature index variability as the intramonthly SD of daily temperature metrics and then average the SDs across months in a given year to yield an annual metric as Eq. 2.

$$\tilde{T_{i,t}}=\frac{1}{12}\sum_{m=1}^{12}\sqrt{\frac{1}{D_{m}}\sum_{d=1}^{D_{m}}{\left(T_{i,d,m,y}-\bar{T_{i,m,y}}\right)}^{2}}$$ (2)

where $\tilde{T_{i,t}}$ is the day-to-day temperature index variability in the county where an older adult i lives during the year preceding time t. $T_{i,d,m,y}$ is the daily temperature metric in the county of i on month m and day d in year y, $\bar{T_{i,m,y}}$ is the monthly mean daily temperature metrics in the county of i in m of y, and $D_{m}$ is the number of days in m of y.

### Excess heat factor

EHF is an index for measuring heatwaves (*64*) based on two EHIs

$$\begin{array}{c} \text{EHI}{\left(\text{accl}.\right)}_{i,d,m,y}=\frac{1}{3}\left(T_{i,d,m,y}+T_{i,d-1,m,y}+T_{i,d-2,m,y}\right)\\ -\frac{1}{30}\left(T_{i,d-3,m,y}+\ldots +T_{i,d-32,m,y}\right) \end{array}$$ (3)

$$\text{EHI}{\left(\text{sig.}\right)}_{i,d,m,y}=\frac{1}{3}\left(T_{i,d,m,y}+T_{i,d-1,m,y}+T_{i,d-2,m,y}\right)-T_{i}^{95}$$ (4)

where $T_{i}^{95}$ is the climatological (i.e., nontime-varying) 95th percentile in the county during 2004–2018. EHI(accl.) describes the anomaly over a 3-day window against the preceding 30 days. Positive values are associated with hot weather or excess heat, and negative values are associated with cool weather. EHI(sig.) describes the anomaly of the same window against an extreme threshold. On the basis of this index, excess heat is typically only possible in the summer half-year, because hot winter weather is not hot by annual standards. The comparison against $T_{i}^{95}$ gives a measure of the statistical significance of the event. Equations 3 and 4 are then combined to obtain EHF

$${\text{EHF}}_{i,d,m,y}=\text{max}\left[1,\text{EHI}{\left(\text{accl}.\right)}_{i,d,m,y}\right]\times \text{EHI}{\left(\text{sig}.\right)}_{i,d,m,y}$$ (5)

where positive values of ${\text{EHF}}_{i,d,m,y}$ define excess heat conditions in the county of the older adult i on month m and day d in the given year y. Because of the multiplication of the EHI(accl.) and EHI(sig.) indices, the unit of EHF is degrees Celsius squared. To compare EHF with other annual temperature metrics, the assessment is made at the annual timescale, so we define the annual mean EHF as

$$E_{i,t}=\sum_{d=1}^{D_{y}}\text{max}\left[0,{\text{EHF}}_{i,d,m,y}\right]/N_{i,t}$$ (6)

where $E_{i,t}$ is the annual mean EHF in the county of an older adult i during the year preceding time t, and $D_{y}$ is the number of days in the year y. We also define the number of days with EHF > 0 on the basis of daily EHF

$$N_{i,t}=\sum_{d=1}^{D_{y}}H\left({\text{EHF}}_{i,d,m,y}\right)$$ (7)

where $N_{i,t}$ is the number of days with EHF > 0 in the county of an older adult i during the year preceding time t, and H is the Heaviside step function, yielding a value of 0 when its argument is less than or equal to 0 and a value of 1 when the argument is greater than 0.

### Statistical analysis

We use a linear probability regression with high-dimensional fixed effects to estimate the impacts of annual temperature metrics on the mortality risk of older adults while controlling for potential confounders. Linear models are known for their stability in parameter estimates and ease of interpretation (*65*, *66*), making them suitable for this analysis. Therefore, we use the specification of model 1 (Eq. 8) to examine the relationships between annual temperature metrics and mortality risk of older adults

$$\begin{array}{c} M_{i,t}=\alpha_{1}\bar{T_{i,t}}+\alpha_{2}\tilde{T_{i,t}}+\alpha_{3}E_{i,t}+\alpha_{4}N_{i,t}\\ +\beta P_{i,t}+\delta Z_{i,t}+\mu_{p,y}+\eta_{p,m}+b+\varepsilon_{i,t} \end{array}$$ (8)

where $M_{i,t}$ represents the mortality risk of an older adult i at time t. The mortality status of older adults in CLHLS samples is a binary 0-1 variable, where 0 represents survival and 1 represents death. Therefore, the model fitting yields $M_{i,t}$, which is the estimated response probability when the mortality status is equal to 1. We refer to it as mortality risk for older adults. For a thorough explanation of why we chose a linear probability model over nonlinear alternatives like logistic regression for binary outcomes, please see Supplementary Text S5.

Our explanatory variables include annual mean temperature index $\bar{T_{i,t}}$, day-to-day temperature index variability $\tilde{T_{i,t}}$, annual mean EHF $E_{i,t}$, and number of days with EHF > 0 $N_{i,t}$, with regression coefficients $\alpha_{1}$ to $\alpha_{4}$, intercept term b, and error $\varepsilon_{i,y}$. We find that the annual mean temperature index based on daily maximum temperature index, the day-to-day temperature index variability calculated from the daily mean temperature index, and the annual mean EHF and number of days with EHF > 0 based on average daily temperature index perform best in assessing the impacts of annual temperature metrics on mortality risk (Supplementary Text S4 and table S7). Therefore, we use model 1, which includes this set of annual temperature metrics, as the baseline model and derive models 2 through 6 based on it. $\mu_{p,y}$ is the province-by-year fixed effect. We control for the year-specific shocks of each province using province-by-year fixed effects (e.g., annual local medical policies, macroeconomic trends, and basic welfare facilities). $\eta_{p,m}$ is the province-by-month fixed effects that remain constant across years. We control for the month-specific shocks of each province using the province-by-month fixed effects (e.g., seasonality of diseases and seasonal variation in lifestyle habits in older adults). The rationale for using these two sets of fixed effects in the baseline model is discussed in detail in Supplementary Text S6.

As additional control variables, the annual total precipitation $P_{i,t}$ in the county of the older adult i during the year preceding time t is estimated. We obtain 5 variables on individual characteristics, 6 variables on family and daily life, 3 variables on education and economic status, 10 variables on dietary habits, and 14 variables on diseases affecting older adults, making a total of 38 variables $Z_{i,y}$ from the CLHLS data used as controls (Supplementary Text S3). β and δ are the coefficients of the control variables. We examine all variables in the baseline model and find no evidence of collinearity (table S15). In addition, to account for autocorrelation within samples from the same living cluster and to address potential heteroskedasticity arising from nonresponse due to death or loss to follow-up among older adults, we compute robust SEs clustered at the county level (Supplementary Text S7). We also conduct several robustness tests on the effects of the four temperature metrics in the baseline regression model (Supplementary Text S8) and justify the inclusion of continuous control variables in a linear form in the baseline model through comparative regressions (Supplementary Text S9).

To further analyze the potential nonlinear effects of the four annual temperature metrics on mortality risk (model 2), we include quadratic terms for the four metrics in the regression model.

$$\begin{array}{c} M_{i,t}=\alpha_{1}\bar{T_{i,t}}+\alpha_{5}{\bar{T_{i,t}}}^{2}+\alpha_{2}\tilde{T_{i,t}}+\alpha_{6}{\tilde{T_{i,t}}}^{2}+\alpha_{3}E_{i,t}+\alpha_{7}{E_{i,t}}^{2}\\ +\alpha_{4}N_{i,t}+\alpha_{8}{N_{i,t}}^{2}+\beta P_{i,t}+\delta Z_{i,t}+\mu_{p,y}+\eta_{p,m}+b+\varepsilon_{i,t} \end{array}$$ (9)

The regression results for model 2 (Eq. 9) are presented in the first column of table S16 and are used in the analysis for Fig. 2. Given that the quadratic effect of the day-to-day temperature index variability is not significant, we also fit the following model (model 3)

$$\begin{array}{c} M_{i,t}=\alpha_{1}\bar{T_{i,t}}+\alpha_{5}{\bar{T_{i,t}}}^{2}+\alpha_{2}\tilde{T_{i,t}}+\alpha_{3}E_{i,t}+\alpha_{7}{E_{i,t}}^{2}\\ +\alpha_{4}N_{i,t}+\alpha_{8}{N_{i,t}}^{2}+\beta P_{i,t}+\delta Z_{i,t}+\mu_{p,y}+\eta_{p,m}+b+\varepsilon_{i,t} \end{array}$$ (10)

The regression results for model 3 (Eq. 10) are shown in the second column of table S16 and are used to compute the county-specific mortality risk associated with annual temperature metrics in Fig. 3. Subsequently, we use model 4 (Eq. 11) to explore the interactive effects between the annual temperature metrics.

$$\begin{array}{c} M_{i,t}=\alpha_{1}\bar{T_{i,t}}+\alpha_{2}\tilde{T_{i,t}}+\alpha_{3}E_{i,t}+\alpha_{4}N_{i,t}\\ +\alpha_{9}\left\{\bar{T_{i,t}}\tilde{T_{i,t}},\bar{T_{i,t}}E_{i,t},\bar{T_{i,t}}N_{i,t},\tilde{T_{i,t}}E_{i,t},\tilde{T_{i,t}}N_{i,t}\right\}\\ +\beta P_{i,t}+\delta Z_{i,t}+\mu_{p,y}+\eta_{p,m}+b+\varepsilon_{i,t} \end{array}$$ (11)

The interaction variables within the curly brackets are sequentially entered into model 4 to evaluate the interactions between temperature warming, temperature variability, and extreme heat.

The effects of annual temperature metrics on mortality risk of older adults may differ across subgroups. To estimate the heterogeneity, we fit model 5 (Eq. 12).

$$\begin{array}{c} M_{i,t}=\alpha_{1}\bar{T_{i,t}}+\alpha_{2}\tilde{T_{i,t}}+\alpha_{3}E_{i,t}+\alpha_{4}N_{i,t}\\ +\beta P_{i,t}+\delta Z_{i,t}+\gamma_{1}z_{i,t}\left\{\bar{T_{i,t}},\tilde{T_{i,t}},E_{i,t},N_{i,t}\right\}\\ +\mu_{p,y}+\eta_{p,m}+b+\varepsilon_{i,t} \end{array}$$ (12)

For model 3 with quadratic terms shown in Eq. 10, we assess heterogeneity by fitting model 6 (Eq. 13).

$$\begin{array}{c} M_{i,t}=\alpha_{1}\bar{T_{i,t}}+\alpha_{5}{\bar{T_{i,t}}}^{2}+\alpha_{2}\tilde{T_{i,t}}+\alpha_{3}E_{i,t}+\alpha_{7}{E_{i,t}}^{2}+\alpha_{4}N_{i,t}\\ +\alpha_{8}{N_{i,t}}^{2}+\beta P_{i,t}+\delta Z_{i,t}+\gamma_{1}z_{i,t}\left\{\bar{T_{i,t}},\tilde{T_{i,t}},E_{i,t},N_{i,t}\right\}\\ +\gamma_{2}z_{i,t}\left\{{\bar{T_{i,t}}}^{2},/,{E_{i,t}}^{2},{N_{i,t}}^{2}\right\}+\mu_{p,y}+\eta_{p,m}+b+\varepsilon_{i,t} \end{array}$$ (13)

where $z_{i,t}$ is the grouping variable of interest, indicating the characteristics of the older adult samples (table S1), including sex, age (divided into four subgroups aged 65 to 75, 75 to 85, 85 to 95, and >95) and the following categorical variables.

Older adults are divided into subgroups according to their obesity status [nonobese, overweight, and obese divided by using the thresholds of BMI = 24 and BMI = 28 according to Chinese obesity standards (*67*)], household per capita annual income [lowest (0 to 2.2 thousand yuan/year), low (2.2 to 6.0 thousand yuan/year), middle (6 to 15 thousand yuan/year), high (15 to 38 thousand yuan/year), and highest (more than 38 thousand yuan/year) according to the quintiles of household per capita annual income in the entire samples], urban-rural residence (i.e., whether the older adults live in urban or rural areas at the time of the survey or death), staple food category (one group with rice or wheat as staple food and the other group with coarse grain as staple food), and whether to eat specific food everyday (inclusive of nine food types: vegetables, fruits, pork, fish, eggs, beans, pickle, tea, and garlic; for each food group, older adults are characterized by whether they consume the specific food every day). In addition, we create interaction terms between the five household per capita annual income subgroups and the two urban-rural residence categories, resulting in a total of 10 subgroups for these interaction models.

The model 5 is primarily used to assess whether the effect of the four annual temperature metrics on mortality risk varies across different subgroups (for Figs. 4 to 6 and tables S21 to S36). The statistical significance of the interaction terms indicates whether the effect difference between the subgroup and the reference group is significant (details of the reference group for each factor variable are provided in Supplementary Text S3). Moreover, model 6, which includes both interaction and quadratic terms, further evaluates whether the observed differences in the model 5 are influenced by variations in local temperature conditions (for figs. S13 to S15).

### Marginal effects and temperature change cumulative effects

The marginal effect is a common concept in economics (*68*–*70*), representing the expected change in a dependent variable resulting from a small change in an independent variable, holding all other factors constant. In recent years, marginal effects have been widely applied in research across various disciplines (*63*, *71*, *72*). The marginal effect is calculated as the partial derivative of the dependent variable with respect to the independent variable, given an equation describing the regression model specification. In the specification shown in model 1 (Eq. 8), the marginal effect of a specific independent variable on mortality risk is equivalent to the regression coefficient of that variable, e.g.,

$${\text{ME}}_{\bar{T_{i,t}}}^{\text{model}\;1}=\frac{\delta M_{i,t}^{\text{model}\;1}}{\delta \bar{T_{i,t}}}=\alpha_{1}$$ (14)

In other regression model specifications with quadratic terms, as shown in models 2 and 3 (Eqs. 9 and 10), the marginal effect of the quadratic term is as follows

$${\text{ME}}_{\bar{T_{i,t}}}^{\text{model}\;3}=\frac{\delta M_{i,t}^{\text{model}\;3}}{\delta \bar{T_{i,t}}}=\alpha_{1}+2\alpha_{5}\bar{T_{i,t}}$$ (15)

${\text{ME}}_{\bar{T_{i,t}}}^{\text{model}\;3}$ represents how a small change in the annual mean temperature index affects mortality risk, and it is a function of the annual mean temperature index. Given the relatively small year-to-year variations in annual temperature metrics within the same region, the marginal effect of the quadratic term essentially reflects regional heterogeneity. This means that the impact of changes in the annual temperature metrics on mortality risk varies across regions due to differences in local climatological temperature conditions.

We used the temperature-mortality relationship derived from model 3 (Eq. 10), which accounts for spatial heterogeneity due to differences in local temperature conditions, to evaluate the cumulative changes in mortality risk attributed to the variations in the four annual temperature metrics in each county. For each county, we calculate the annual differences in mortality risk linked to the four annual temperature metrics for each year over 2005–2018, relative to 2004. We then sum these differences to represent the cumulative impacts of changes in the four annual temperature metrics. For example, the cumulative change in mortality risk linked to the annual mean temperature index is as follows

$${\text{CE}}_{\bar{T_{i,y}}}^{2005-2018}=\sum_{y=2005}^{2018}\left(\alpha_{1}\bar{T_{i,y}}+\alpha_{5}{\bar{T_{i,y}}}^{2}-\alpha_{1}\bar{T_{i,2004}}-\alpha_{5}{\bar{T_{i,2004}}}^{2}\right)$$ (16)

Both coefficients $\alpha_{1}$ and $\alpha_{5}$ in Eq. 16 are derived from model 3. $\bar{T_{i,y}}$ denotes the computed annual mean temperature index at the county level by using the ERA5 climate data for the years 2004–2018. The cumulative changes in the mortality risks linked to the other three annual temperature metrics are calculated in the same manner. From this, we obtain the cumulative effects from the four annual temperature metrics on the mortality risks of older adults in each county and further analyze their spatial patterns by mapping these effects.

## Supplementary Materials

### This PDF file includes:

Supplementary Text S1 to S9 Figs. S1 to S15 Tables S1 to S36 References
