# Supplementary material for: Elderly vulnerability to temperature-related mortality risks in China
Source: Sci Adv. 2025 Feb 5;11(6):eado5499. doi: 10.1126/sciadv.ado5499 (PMC11797541; doi:10.1126/sciadv.ado5499)
Supplement: Supplementary file 1 — Supplementary Text S1 to S9 Figs. S1 to S15 Tables S1 to S36 References [file sciadv.ado5499_sm.pdf]

Science Advances
AAAS

Supplementary Materials for

Elderly vulnerability to temperature-related mortality risks in China

Xin Yao *et al.*

Corresponding author: Liqiang Zhang, zhanglq@bnu.edu.cn; Chen Bai, baichen-slhr@ruc.edu.cn; Jintai Lin, linjt@pku.edu.cn

Sci. Adv. **11**, eado5499 (2025)
DOI: 10.1126/sciadv.ado5499

This PDF file includes:

Supplementary Texts S1 to S9
Figs. S1 to S15
Tables S1 to S36
References

Supplementary Text

Supplementary Text 1: Adjust administrative codes of the counties

To protect the privacy of the older adults in CLHLS, their exact geographic coordinates are obscured, and we can only match each older adult sample with the temperature metrics of their counties of residence (Fig. S2E). However, between 2005 and 2018, 109 county administrative codes were changed for various reasons. To address this issue, we reassigned new administrative codes to the samples of older adults whose codes changed. Specifically, there are four types:

Firstly, there are 16 counties that have been merged with neighboring counties to form new counties due to the county consolidation policy, and their real geographic locations do not change. For example, in July 2010, the Chinese State Council approved the revocation of Xicheng District (110102) and Xuanwu District (110104) in Beijing, and established a new Xicheng District (110102). We replace the older administrative codes with the new ones of the merged counties.

Secondly, there are 85 counties that have formed new counties due to policies such as "Abolishing counties and establishing districts", "Abolishing cities and establishing districts", "Abolishing districts and establishing counties", "Abolishing districts and establishing cities", and "Adjusting administrative regions". The administrative regions of these counties do not change substantially or at all. For example, in April 2015, the Chinese State Council approved the abolishment of Xushui County (130625) and the establishment of Xushui District (130609). We replace the original administrative codes of these counties with the new ones after the adjustment.

Thirdly, there are 7 counties where administrative codes were recorded incorrectly during the survey, leading to the inability to match temperature metrics. We have corrected the erroneous county codes, such as recording Hetang District (430202) as Hetang District (430220).

Fourthly, there is one county that has been split into multiple counties. For example, the Daxing'anling area (232700) was split into Mohe City (232701), Tahe County, and Huma County. We replace the original county code with the code of the county with the largest area after the split.

Supplementary Text 2: Daily temperature index similar to the heat index

The calculation of the heat index follows a multi-step procedure developed by Steadman (73, 74), along with a regression equation and several adjustments (75). These equations consider the relationship between temperature and relative humidity. The procedure for the calculation of heat index is available at https://www.wpc.ncep.noaa.gov/html/heatindex_equation.shtml and can be summarized in the following five steps:

Step 1: Calculate the heat index using the simple formula provided by Steadman (73, 74) given as,

$$HI = 0.5 \times \{T + 61.0 + [(T - 68.0) \times 1.2] + (RH \times 0.094)\} \quad \text{(S.1)}$$

where *T* is the temperature in °F, and *RH* is relative humidity in percent. *HI* is the heat index expressed as an apparent temperature in °F.

Step 2: Average the *HI* with the *T*.

Step 3: If the result in step 2 is no less than 80°F, then we apply the regression equation provided by Rothfusz (75) given as,

$$\begin{aligned} HI =& -42.34 + 2.05 \times T + 10.14 \times RH - 0.22 \times T \times RH - 0.0068 \times T \times T \\ & -0.05481717 \times RH \times RH + 0.0012 \times T \times T \times RH + 0.00085 \times T \times RH \times RH \\ & -0.00000199 \times T \times T \times RH \times RH \end{aligned} \quad \text{(S.2)}$$

Step 4: If the *RH* is less than 13% and *T* is between 80°F and 112°F, then the following adjustment is subtracted from the *HI* calculated in Step 3.

$$HI_{adj(-)} = \frac{13-RH}{4} \times \sqrt{\frac{17-|T-95|}{17}} \quad \text{(S.3)}$$

Step 5: If *RH* is greater than 85%, and *T* is between 80°F and 87°F, then the following adjustment is added to *HI* calculated in Step 3.

$$HI_{adj(+)} = \frac{RH-85}{10} \times \frac{87-T}{5} \quad \text{(S.4)}$$

The heat index is typically calculated using daily max temperature. In this study, we compute the heat index from four fundamental daily temperature metrics, resulting in the daily max temperature index, daily mean temperature index, daily min temperature index and average daily temperature index (Fig. S1).

Supplementary Text 3: Control variables

In the regression models, in addition to the province-by-year fixed effects, province-by-month fixed effects and the climate control of annual precipitation, we include 38 survey variables from five dimensions as the control variables to enhance the description of the model and reduce the interference of the confounding factors (Table S1).

The first dimension characterizes the individual characteristics of CLHLS older adult samples, including their sex, age, ethnicity, BMI (calculated from height and weight), and the interviewer's health rating of older adults. Among them, sex is a binary factor variable, with 0 representing "Man" and 1 representing "Woman", and "Man" is used as the sex reference group in all regression models. Age is a continuous variable obtained by subtracting the older adults' birth date from their survey or death date. Ethnicity is a multi-value factor variable assigned based on the older adults' ethnic group, including Han, Hui, Zhuang, Yao, Korea, Man, Mongolia, and others. "Han" is used as the ethnicity reference group in all regression models. BMI is factor variable categorized according to Chinese obesity standard (67), with BMI = 24 and BMI = 28 as thresholds, dividing into non-obese, overweight, and obese groups. "BMI < 24 (non-obese)" is used as the BMI reference group in all regression models. The interviewer's health rating of older adults includes four levels: "1-very healthy", "2-relatively healthy", "3-weak", and "4-very ill". In all regression models, "1-very healthy" is used as the health rating reference group.

The second dimension characterizes the family information and daily life of CLHLS older adult samples, including their cohabitation situation, marital status, whether they have children, smoking status, drinking status, and exercise status. Among them, the cohabitation situation includes "1-living with family", "2-living alone", "3-living in a nursing institution". In all regression models, "1-living with family" is used as the cohabitation situation reference group. Marital status includes "1-currently married, living with spouse", "2-separated", "3-divorced", "4-widowed", "5-never married". In all regression models, "1-currently married, living with spouse" is used as the marital status reference group. The presence or absence of children is a binary factor variable, with 0 representing "No children" and 1 representing "Having children", and "No children" is used as the children reference group. Smoking status, drinking status, and exercise status are each divided into four levels from low to high, namely "1-never smoked/drank/exercised", "2-previously did not smoke/drink/exercise but currently smoke/drink/exercise", "3-smoked/drank/exercised in the past", "4-consistently smoke/drink/exercise". In all regression models, "1-never smoked/drank/exercised" are used as the smoking/drinking/exercise status reference groups.

The third dimension characterizes the education and economic status of CLHLS older adult samples, including the urban-rural residence, education years, and the household per capita annual income. Among them, the urban-rural residence is a binary factor variable, with 0 indicating living in rural areas and 1 indicating living in urban areas, and "0-Rural" is used as the urban-rural reference group in all regression models. The number of education years is a continuous variable, measured in years. This variable does not distinguish between different levels of education, and represents the total number of years the respondent received any form of education prior to the survey. The household per capita annual income is a continuous variable that reflects the economic status of the older adult families.

The fourth dimension characterizes the dietary habits of older adult samples in CLHLS, including the staple food category and whether to eat specific food (vegetables, fruits, pork, fish, egg, bean, pickle, tea, and garlic) everyday. The staple food category includes “0-rice and wheat” and “1-coarse grain”, and “0-rice and wheat” is used as the staple food reference group in all regression models. Whether to eat specific food everyday includes nine binary factor variables, with 0 indicating “Not eat everyday” and 1 indicating “Eating everyday”, and "Not eat everyday" are used as the specific food reference groups in all regression models.

The fifth dimension characterizes the diseases suffered by the older adult samples, including hypertension, diabetes, heart disease, stroke and cerebrovascular disease, bronchitis/emphysema/pneumonia and asthma, tuberculosis, cataracts, glaucoma, cancer, gastrointestinal ulcers, Parkinson's disease, pressure ulcers, arthritis, and dementia. All of the above diseases are binary factor variables, with 0 indicating the absence of the disease, and 1 indicating the presence of the disease. In all regression models, “Absence of the disease” are used as the disease reference groups.

Supplementary Text 4: Optimal daily temperature metrics for assessing the impact of the annual temperature metrics on mortality risk

The daily temperature metrics reflect temperatures at various times of the day, such as daytime and nighttime. Because these metrics are highly correlated and exhibit collinearity when derived from different daily temperature measures (Tables S4-S6), we could not include all of them in the regression analysis simultaneously, expect for those based on daily maximum and minimum temperatures (indices).

To determine the most effective daily temperature metrics for evaluating the impact of the annual temperature metrics on mortality risk, we conduct three sets of regressions based on the specification of Model 1, totaling 30 regressions, and evaluate the performance of each regression model. In the first set of regressions, we progressively vary the daily temperature metric used to calculate the annual mean temperature index (a total of 8 times). Then, we include the annual mean temperature indices based on daily max and min temperatures (the ninth regression), as well as those based on daily max temperature index and daily min temperature index (the tenth regression). From the ten regressions, we select the optimal daily temperature index for evaluating the impact of annual mean temperature index on mortality. Based on this, we then progressively vary the daily temperature indices used to calculate day-to-day temperature index variability (second set of ten regressions) and the daily temperature indices used to calculate annual EHF metrics (third set of ten regressions). It is important to note that the annual mean EHF and the number of days with EHF > 0 represent two different characteristics of extreme heat events. Therefore, the daily temperature metrics used to calculate these two annual temperature metrics should be the same within the same regression model.

We use the Akaike Information Criterion (AIC) and the Bayesian Information Criterion (BIC) as the primary evaluation criteria for model selection in assessing the regression models. AIC and BIC are evaluated using a trade-off between the maximized likelihood function and penalties for additional model terms which could result in overfitting. As such, they are used to assess the relative strength of different models in terms of best describing the data and limiting the possibility of overfitting (76-78).

Additionally, we conduct five-fold cross-validation for each regression model. This process involves splitting the sample data into five equal parts, or "folds", using four of these folds for model training and the remaining fold for testing. This process is repeated five times, with each fold serving as the test set, while the other four folds are used for fitting. The final performance metric is the average of the five iterations. Cross-validation can evaluate the model's robustness to different data distributions by randomly shuffling the data in each fold, thereby assessing the model performance on unknown data. We use a threshold of 0.5 to classify the predicted mortality risk from the regression models into "0-Survival" and "1-Death". These predictions are then compared with the actual survival status of the older adult samples. According to convention, we denote death samples as "Positive (P)", survival samples as "Negative (N)", correct model predictions as "True (T)", and incorrect predictions as "False (F)".

Specifically, "TP" (True Positive) refers to instances where both the model predicts "Death" and the actual outcome is "Death"; "FP" (False Positive) denotes cases where the model predicts "Death" but the actual outcome is "Survival"; "FN" (False Negative) indicates cases where the

model predicts “Survival” but the actual outcome is “Death”; and “TN” (True Negative) signifies instances where both the model predicts “Survival” and the actual outcome is “Survival”. Precision is calculated as the proportion of true positive predictions out of all positive predictions made by the model, reflecting the accuracy of the positive predictions.

Precision is given by

$$\text{Precision} = \frac{\text{TP}}{\text{TP+FP}} \tag{S.5}$$

Recall measures the completeness of the positive predictions, which is the proportion of the true positive predictions among all actual positive samples in the samples. Recall is given by

$$\text{Recall} = \frac{\text{TP}}{\text{TP+FN}} \tag{S.6}$$

F1 score is the harmonic mean of precision and recall, ranging from 0 to 1, with 1 indicating perfect precision and recall. A high F1 score indicates that a model has both high precision and high recall. It is expressed by Eq. (S.7).

$$\text{F1} = \frac{2\times\text{Precision}\times\text{Recall}}{\text{Precision+Recall}} \tag{S.7}$$

Supplementary Text 5: Comparison between linear probability model and logistic regression

Nonlinear models such as logit and probit are commonly used for regression modeling when the outcome is binary, although advances in statistics and methods have established that this is often not optimal, justified or appropriate (65, 66, 79, 80). The main argument in favor of nonlinear models such as logit or probit for analyzing binary outcomes is that these models constrain predictions to fall between 0 and 1. A different but related argument in favor of nonlinear models argument is that linear probability model (LPM) coefficients may be biased and inconsistent for binary outcomes, particularly because such models can produce predictions outside the [0, 1] interval of the outcome variable. Horrace and Oaxaca (81) demonstrated that bias and inconsistency of the estimator increase with the proportion of predicted probabilities that fall outside of the support and recommend the use of logit or probit models.

There are several reasons to prefer the linear probability model over nonlinear models such as logit and probit when the outcome is binary. The linear probability model allows for the direct interpretation of coefficients as probabilities and is more reliable when the model includes fixed effects or interaction terms. In contrast, logit and probit coefficients are not immediately interpretable; converting them into probabilities requires additional complexity through methods such as marginal standardization, prediction at the means, or prediction at the modes (65, 79, 82). Moreover, nonlinear models like logit and probit become unsuitable in the presence of high-dimensional fixed effects (79, 83). Additionally, the linear probability model utilizes all available data in the regression, whereas the logit and probit models exclude groups that show no variation in the dependent variable ("homogeneous groups"). Consequently, the logit and probit models are estimated on a subset of the data used by the linear model, which may be substantially smaller, especially when outcomes are rare events (83).

In the study, the aim is to explain the impact of temperature change on mortality risk rather than to predict mortality outcomes. Therefore, the appropriateness of using a linear probability model for binary outcomes depends on whether the out-of-bound predictions from this model introduce bias into the effect estimates. We design three sets of comparative experiments to examine this issue by comparing the marginal effects estimated by logistic regression at the sample mean with those estimated by the linear probability model (Fig. S6; Table S8). Given that logistic regression performs poorly with high-dimensional fixed effects (84, 85), we compare the linear probability model and logistic regression under the following conditions: (i) without any fixed effects; (ii) with province and year fixed effects; and (iii) with province, year, and month fixed effects. Additionally, we calculate the summary statistics (mean, standard deviation, maximum, minimum, and the proportion of sample predictions outside the [0,1] interval) for the mortality risk of older adults as estimated by these three linear regression models and the baseline model (Table S9). In the three sets of regressions with different parameter settings, the effects of the four annual temperature metrics on mortality risk obtained from both the LPM and logistic regression models are similar in nature (Fig. S6). Moreover, in the three linear regressions for sensitivity tests, only a small number of predictions fall outside the [0, 1] range (8.66%, 0.19%, and 0.04%, respectively), whereas in the baseline model, only 0.03% of the predictions exceed the [0, 1] range (Table S9), indicating that the use of the linear probability model is reasonable and reliable in this study.

Supplementary Text 6: Comparison of the regression specifications with different fixed effects

The baseline model, along with all subsequent models used for heterogeneity analysis with interaction terms (Models 2-6), includes two sets of high-dimensional fixed effects: (i) year-specific shocks of each province using province-by-year fixed effects, which may include annual local medical policies, macro-economy, and basic welfare facilities; and (ii) the month-specific shocks of each province using the province-by-month fixed effects (e.g., seasonality of diseases and seasonal variation in lifestyle habits in older adults). The province-by-year fixed effects include a set of indicator variables for each province (23 of them) in each year (14 years), resulting in a total of 307 indicator variables (note that not all provinces have samples in each year). The province-by-month fixed effects consist of a set of indicator variables for each province (23 of them) in each month (12 months), leading to a total of 276 indicator variables (again, not all provinces have samples in each month). The province-by-month fixed effects remain constant across years. These fixed effects allow us to account for unobserved differences between provinces, contemporaneous province shocks, and province-specific time trends (e.g., multi-year socioeconomic and demographic trends of each province), strengthening the precise identification of the association between temperature change and mortality risk.

The inclusion of a greater number of fixed effects in the model generally enhances the fit of the linear regression (83). However, as the number of fixed effects increases, the number of model parameters also rises rapidly, leading to potential overfitting. This overfitting in fixed effects panel models can result in standard errors of the effect coefficients becoming too small (86). Therefore, we conduct two sets of comparative regressions to test the appropriateness of the fixed effects used in the baseline model (Fig. S7; Table S10). (i) We only include province-by-year fixed effects but exclude province-by-month fixed effects in the baseline regression model to avoid model fishing and overfitting. (ii) We add province-by-day fixed effects (a set of indicator variables for each province in each day of month (31 days), leading to a total of 712 indicator variables; note that not all provinces have samples in each day of month) to the baseline regression model besides province-by-year fixed effects and province-by-month fixed effects to further control for the date-specific shocks across provinces (such as fixed dates for wage and pension payments). The province-by-day fixed effects remain constant across both years and months. Comparing regressions with more stringent fixed effects to our baseline model can indicate whether our current fixed effects are well-controlled, thereby ensuring a true and robust relationship between temperature change and mortality risk.

The similarity of the effect coefficients across the three regressions indicates that our current fixed effects are reasonable and robust, and do not lead to model overfitting (Fig. S7; Table S10). Another noteworthy issue is that the province-by-month fixed effects in our model may obscure some short-term effects of temperature changes. However, since each older adult is matched with temperature metrics from the entire year preceding their survey or death date, the month in which the older adult was surveyed or died (the primary control of province-by-month fixed effects) is unlikely to influence our four annual temperature metrics. Considering that the monthly differences in mortality risk among older adults may also arise from various seasonal confounding factors (such as the seasonality of diseases, precipitation, air pollution, and other climatic factors, as well as lifestyle variations across different seasons), we ultimately decide to retain province-by-month fixed effects in the baseline model.

Supplementary Text 7: Comparison between balanced and unbalanced panel regressions and the adjustment of standard errors

In this study, some older adults may have exited the panel data due to “nonresponse” such as death or loss of contact, resulting in varying numbers of data entries for different individuals. Consequently, the panel data became an “unbalanced panel”. Unbalanced panels are likely to be the norm in typical empirical research settings (87). The individual nonresponse leading to an unbalanced panel can be categorized into “random and ignorable” and “non-random and non-ignorable” types (88). If the nonresponse reason is random for the parameters of interest, one can use the standard panel data methods for consistent estimation (87, 88). If the nonresponse reason is non-random, it may lead to heteroskedasticity in the residuals (87, 88).

We initially use two balanced subsets of older adults to conduct comparative regressions, examining the differences in the effect coefficients of the annual temperature index between the baseline model and the balanced panel condition (Columns 1 and 2 in Table S11).To address the impact of sample size on the regression, we select the two largest balanced subsets of the data: older adults who participated in two survey waves (5,975 individuals and 11,950 observations) and those who participated in four waves (2,566 individuals and 10,264 observations). In these two regressions, we use robust standard errors without clustering at any level. The effect coefficients for the four annual temperature metrics in these regressions are slightly lower than those in the baseline model, but all effects remain statistically significant (Columns 1, 2, and 4 in Table S11). Another common method for handling individual nonresponse in panel data is to apply weighted adjustments to the regression. This involves modeling the response propensities for each sampling unit or group of units and using the inverse of these estimated propensities as weighting factors in the regression (89). Our study focuses on the effects of annual temperature metrics on mortality risk and addresses potential heteroskedasticity in the residuals by adjusting the standard errors. Rather than modeling response propensities for older adults, we use the inverse of the number of survey waves each older adult participated in as a simple weighting adjustment factor for the comparative regression (see Column 3 in Table S11). The effects of the four annual temperature metrics in the weighted regression are comparable to those in the baseline model (see Columns 3 and 4 in Table S11). Modeling response propensities for each older adult presents an interesting area for future research on individual nonresponse, but it is beyond the scope of this manuscript.

Cluster-robust standard errors have become increasingly common in empirical research in recent years (90, 91). The basic idea is to divide the sample into disjoint clusters, such as provinces, counties, and years. Any pattern of heteroskedasticity and/or dependence is allowed within each cluster, but it is assumed that there is independence across clusters and that the assignment of observations to clusters is known (91). Additionally, cluster-robust standard errors can adjust for correlations induced by sampling the outcome variable from a data-generating process (90). In this study, we calculate cluster-robust standard errors for the baseline model at five levels: county, individual, province, year, and month (Columns 4-8 in Table S11). The effects of the four annual temperature metrics are statistically significant across all clustering levels (Columns 4-8 in Table S11). Therefore, we use cluster-robust standard errors at the county level in the baseline model to address the heteroskedasticity issue associated with the unbalanced panel.

Supplementary Text 8: Robustness checks

We conduct multiple robustness tests which indicate that the estimated results in the baseline regression are not qualitatively influenced by additional confounding factors (Fig. S8; Table S12).

Firstly, we exclude older adult samples from Guangxi in the baseline regression (Row 2 in Fig. S8; Column 2 in Table S12). Among the 23 provinces and municipalities surveyed in CLHLS, the cumulative death toll in Guangxi is substantially higher than in other provincial-level administrative regions (Fig. S2C). Regression without samples of Guangxi excludes the possibility that the response is dominated by a province with particularly high mortality risk of older adults. The regression results after excluding samples are similar to those in the baseline regression (Fig. S8; Column 1, 2 in Table S12).

Secondly, we find that the highest number of deaths among older adults in CLHLS from 2005 to 2018 occurred in 2006, which may introduce some errors in our estimation due to the influence of specific events on death. Therefore, we exclude the samples of older adults in 2006 for regression analysis, and find that the results are similar to the baseline regression (Row 3 in Fig. S8; Column 3 in Table S12).

Thirdly, we add two additional control variables to the baseline regression, including self-reported living conditions and self-reported health status of older adults (Row 4 in Fig. S8; Column 4 in Table S12). Both are divided into five levels: “1-very good”, “2-good”, “3-fair”, “4-poor”, and “5-very poor”. In the regression, “1-very good” is used as the reference group. The estimates remain unchanged in quantity after their inclusion (Row 4 in Fig. S8; Column 4 in Table S12).

Fourth, we remove all control variables from the model, retaining only the four annual temperature metrics and annual precipitation. The effects of the four annual temperature metrics in this regression are similar to those in the baseline model, but with slightly larger standard errors (Row 5 in Fig. S8; Column 5 in Table S12).

Fifth, we use different climate re-analysis datasets (EarthH2Observe, WFDEI, and ERA-Interim data merged and bias-corrected for the Inter-Sectoral Impact Model Inter-Comparison Project, EWEMBI) to test the consistency of assimilation and interpolation techniques used in the observational data reanalysis. After replacing the climate data, the effect of annual temperature metrics on mortality risk of older adults shows some fluctuations, but the overall trend and level of effects remain largely unchanged (Row 6 in Fig. S8; Column 6 in Table S12).

Supplementary Text 9: Comparison of the regression specifications with different age-mortality functions

Linear models are often the preferred choice for causal inferences in empirical research (65, 66). Advances in econometric and statistical theory and methods explain this choice primarily from three perspectives: (i) Under the assumption that the conditional expectation function is linear, the overall regression equation should be a linear function (The Linear Conditional Expectation Function Theorem, LCEFT); (ii) Given the independent variable X, the function βX is the best linear predictor of the dependent variable in terms of minimizing mean squared error (The Best Linear Predictor Theorem, BLPT); (iii) The function βX provides the optimal linear approximation of $E[Y|X]$ in terms of minimizing mean squared error (The Regression Conditional Expectation Function Theorem, RCEFT). Theorems (ii) and (iii) offer two additional perspectives on the linear regression. On one hand, the linear regression provides the best linear estimate of the dependent variable even without relying on the linearity assumption. On the other hand, if we consider approximating $E[Y|X]$ rather than predicting Y, the RCEFT tells us that, even if the conditional expectation function is not linear, the linear regression still provides the best linear approximation.

Considering that the aim of this study is to assess the effects of four annual temperature metrics on mortality risk, we use the linear regression for all variables in the baseline model. However, age is likely to be critically important for estimating mortality risk, and the linear treatment of the age-mortality relationship may warrant scrutiny. In addition to the linear age-mortality relationship in the baseline model, we perform five regressions by including age in the form of a quadratic function, a cubic function, a spline function with three knots, a spline function with four knots, and a factor variable with four levels (65-75 years, 75-85 years, 85-95 years, and over 95 years). In the six regressions, the effects of the four annual temperature metrics show almost no difference (Fig. S9; Table S13).

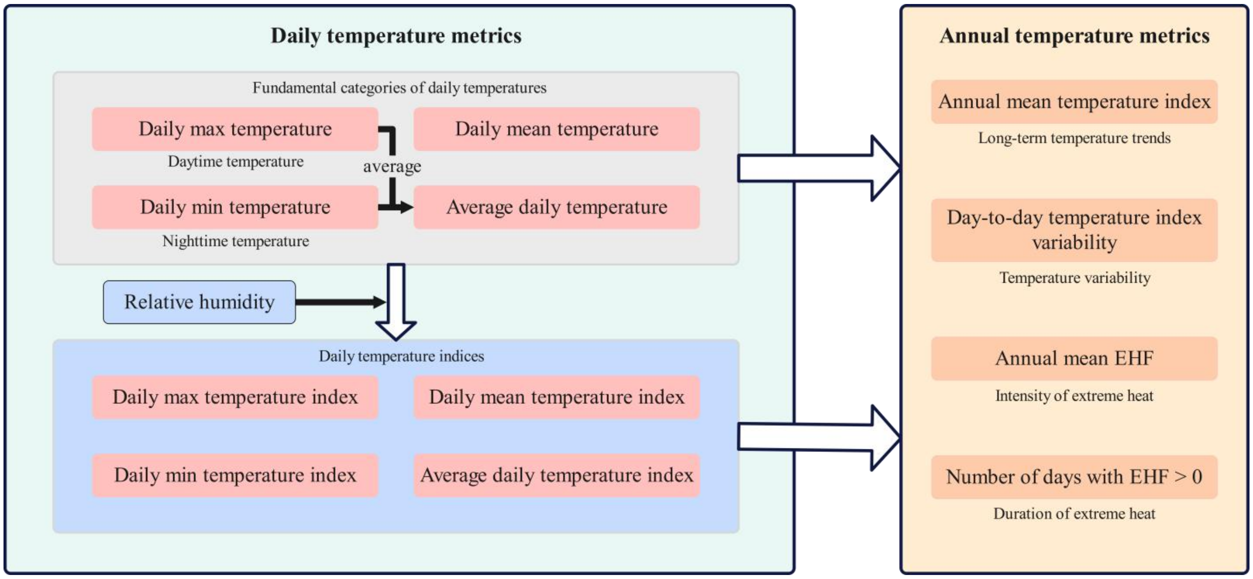

Fig. S1. Temperature metrics at different timescales and related indicators. The four day temperature metrics in the light pink box represent temperature conditions at different times of the day. The four temperature indices in the light blue box are derived by considering the interaction between the day temperature metrics from the light pink box and relative humidity. Each of the eight daily temperature metrics is then used to generate a set of four distinct annual temperature metrics (orange box). These annual metrics describe the long-term trends and variability, as well as the intensity and duration of extreme heat, measured on an annual timescale.

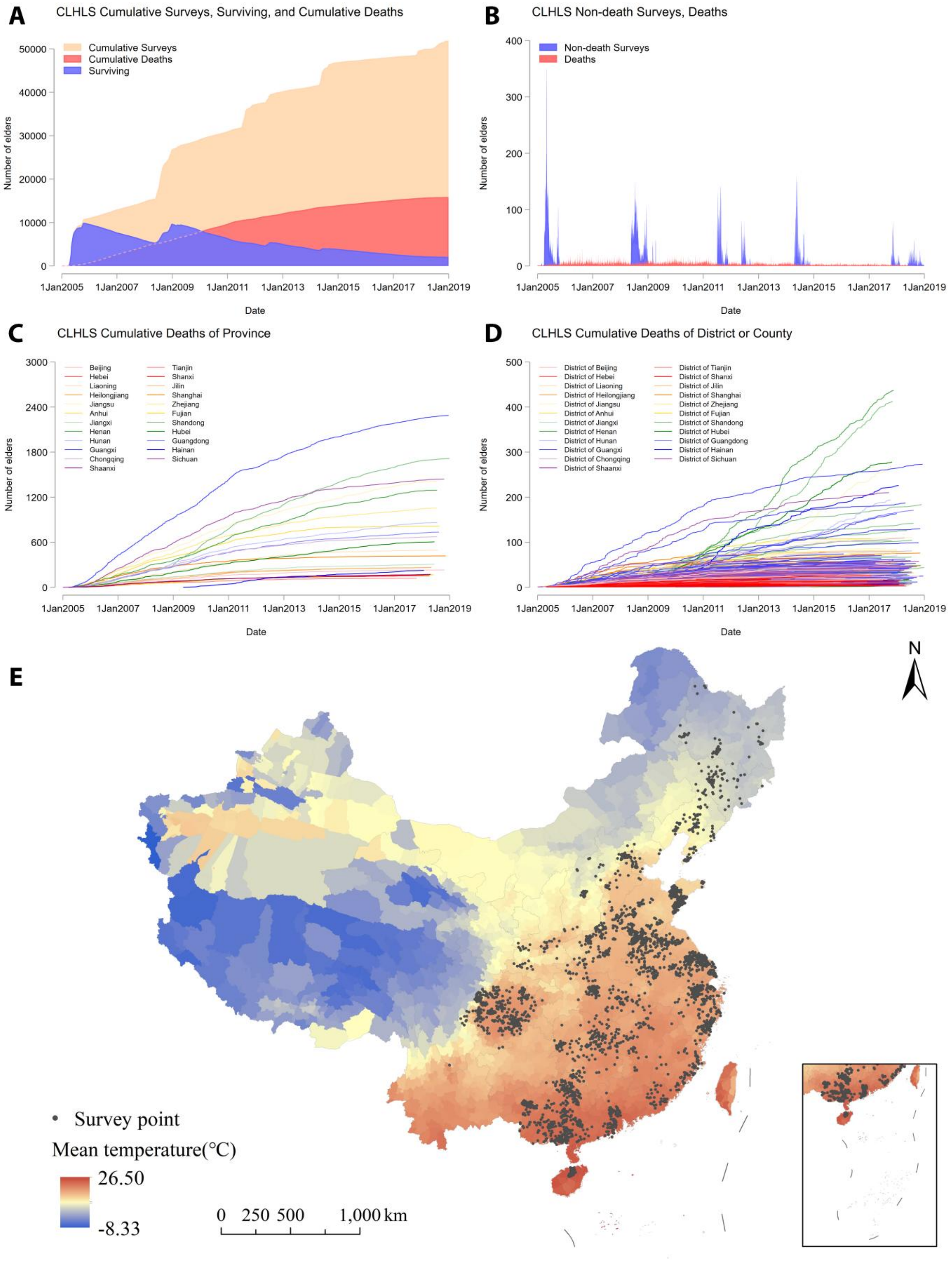

Fig. S2. Survey and mortality of CLHLS older adults. A, **B**, The CLHLS samples from January 1, 2005 to December 31, 2018. **A**, The cumulative number of survey cases (person-times), the cumulative number of deaths of older participants (person), and the current number of

surviving older participants (person). **B**, The daily number of survey cases of surviving older participants (person-times) and the daily number of deaths of older participants (person). **C**, the cumulative number of deaths of older participants in 23 provinces and autonomous regions surveyed in the CLHLS, with different colors of lines representing different provincial-level administrative regions. **D**, The cumulative number of deaths of older participants in 917 counties surveyed in the CLHLS, with different colors of lines representing different provincial-level administrative regions. **E**, The black dots represent the survey locations in the CLHLS. To protect the privacy of the survey participants, the exact coordinates of the survey locations are not obtained. Therefore, the dot locations do not represent the exact coordinates of the participants but only indicate the counties where the participants are located. The base map shows the mean temperature from 2004 to 2018.

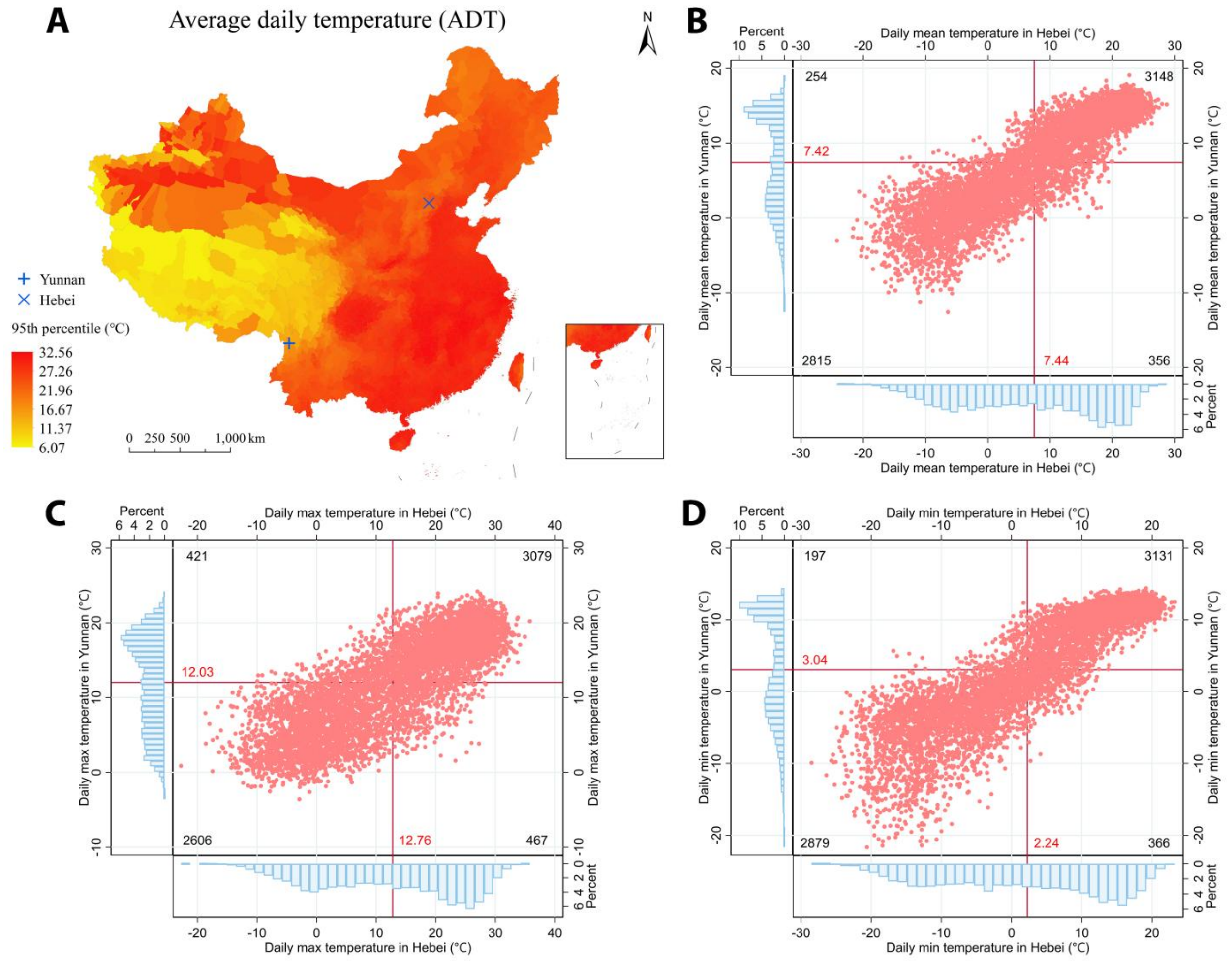

Fig. S3. The distribution of daily temperature. **A**, Spatial distribution of the 95th percentile of average daily temperature (the average of the maximum temperature and the minimum temperature within a 24-hour cycle, see Materials and Methods) during 2004-2018. The blue plus and cross represent Weixi County in Yunnan Province, and Yue County in Hebei Province, respectively, as examples. **B**-**D**, The distributions of daily mean temperature in (**B**), daily maximum temperature in (**C**), and daily minimum temperature in (**D**) in the two counties, which have a large disparity, despite their similar annual mean temperature. The red dots represent the temperatures of the two locations on the same day, the blue histograms represent the marginal distribution of daily temperatures in each location, and the red orthogonal lines represent the yearly average of daily temperature in the two counties.

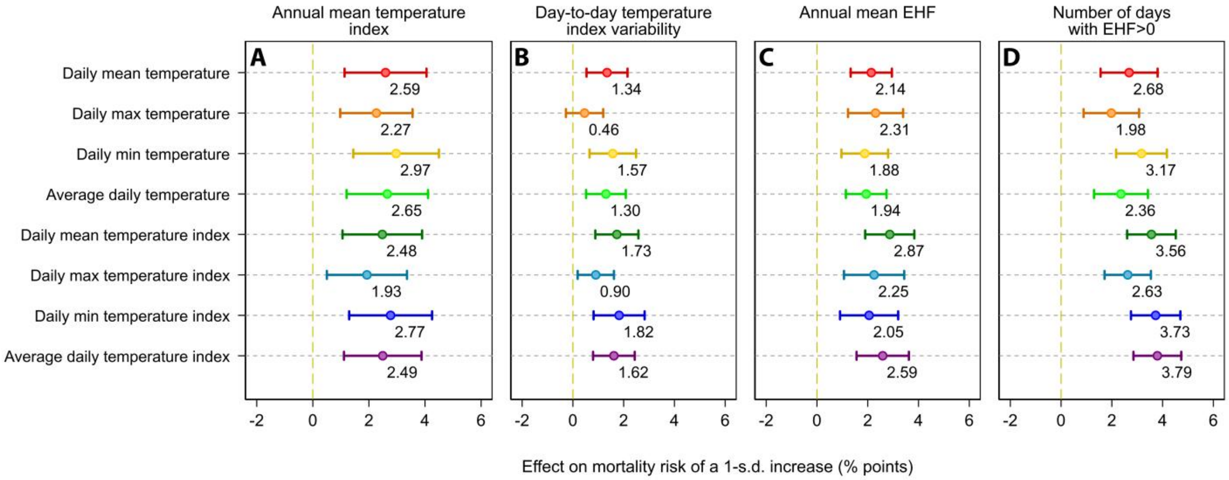

Fig. S4. Effects of the four annual temperature metrics based on each of eight daily temperature metrics on mortality risk of older adults. Estimates of the effect on mortality risk of a 1-s.d. increase in each annual temperature metric. Points and lines represent mortality effect estimates and their corresponding 95% confidence intervals (CIs), respectively. Each row in the figure (error bars of the same color) represents a separate fixed-effects regression based on the specification of Model 1, where the explanatory variables include four different annual temperature metrics derived from a specific daily temperature metric. Province-by-year fixed effects and province-by-month fixed effects are included in each regression, and standard errors are clustered at the county level. More details are presented in Table S2.

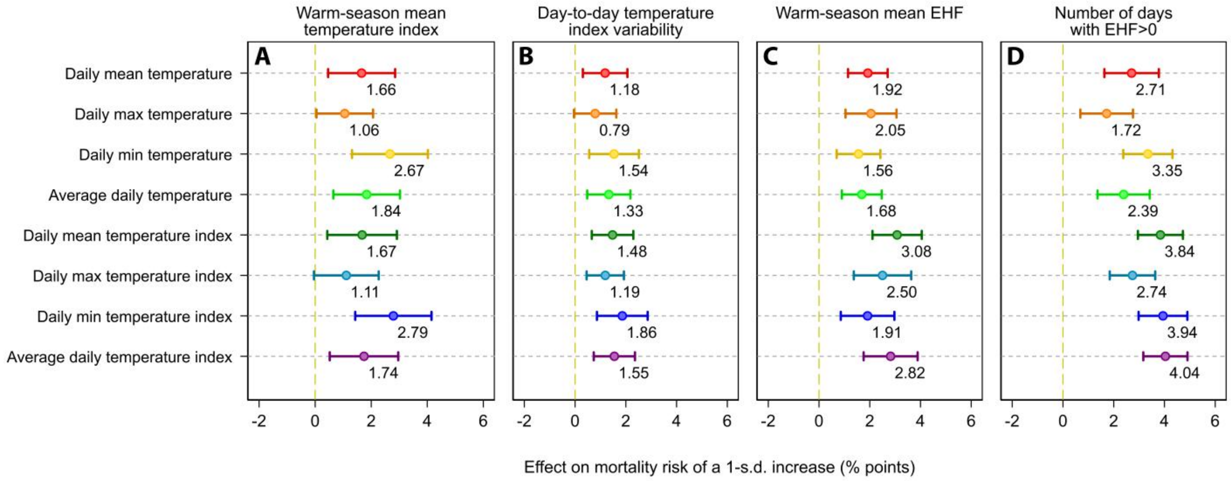

Fig. S5. Effects of the four warm-season temperature metrics based on each of eight daily temperature metrics on mortality risk of older adults. Estimates of the effect on mortality risk of a 1-s.d. increase in each warm-season temperature metric. Points and lines represent mortality effect estimates and their corresponding 95% CIs, respectively. Each row in the figure (error bars of the same color) represents a separate fixed-effects regression based on the specification of Model 1, where the explanatory variables include four different warm-season temperature metrics derived from a specific daily temperature metric. Province-by-year fixed effects and province-by-month fixed effects are included in each regression, and standard errors are clustered at the county level. More details are presented in Table S3.

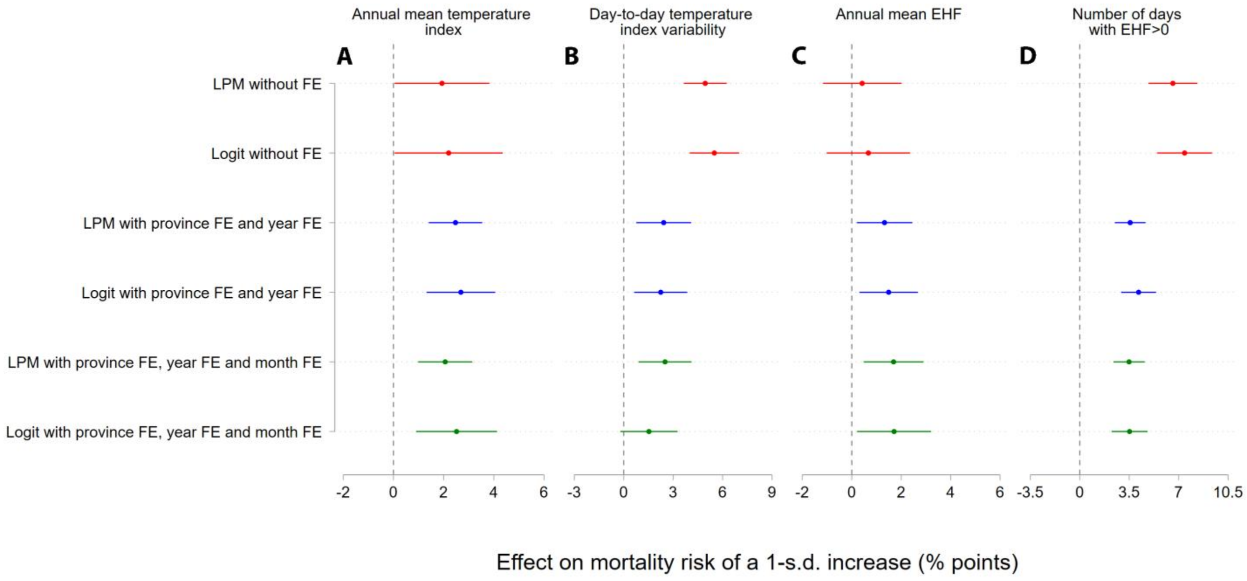

Fig. S6. Comparison of estimates of the effect on mortality risk using the linear probability model (LPM) and logistic regression. Estimates of the effect on mortality risk of a 1-s.d. increase in each annual temperature metric. Points and lines represent mortality effect estimates and their corresponding 95% CIs, respectively. For logistic regression, the marginal effect estimates refer to the marginal effects at the sample means. Each row in the figure represents a separate regression. The regressions in the first and second rows do not include fixed effects. The regressions in the third and fourth rows include province fixed effects and year fixed effects. The regressions in the fifth and sixth rows include province fixed effects, year fixed effects, and month fixed effects. Standard errors in all regressions are clustered at the county level. More details are presented in Supplementary Text 5 and Table S8.

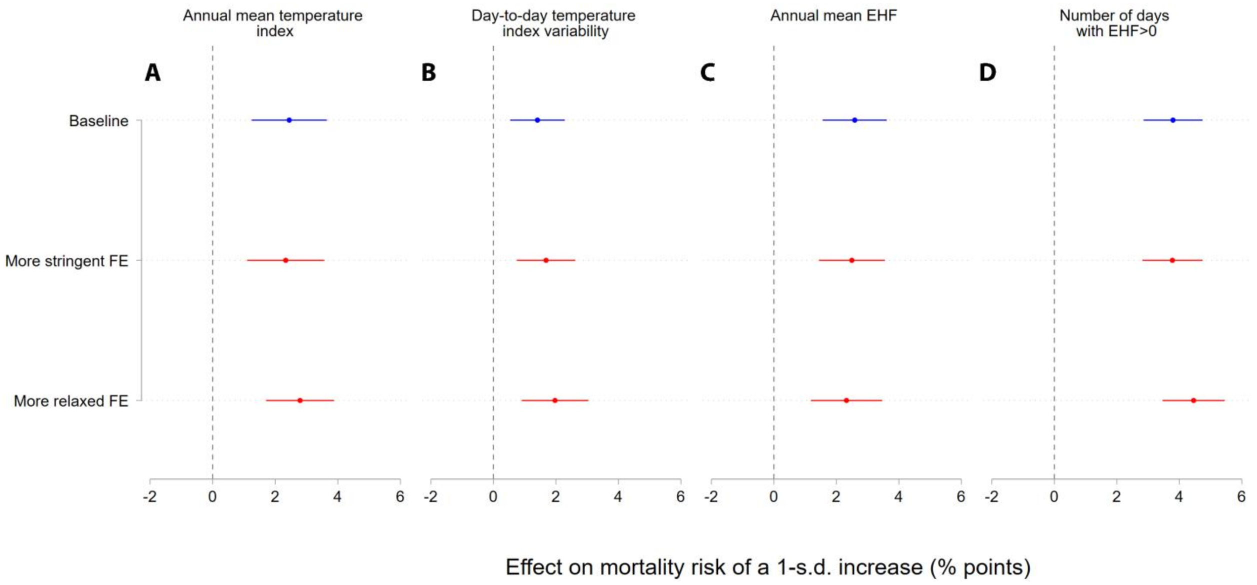

Fig. S7. Comparison of the regression specifications with different fixed effects for the effects of annual temperature metrics on mortality risk of older adults. Estimates of the effect on mortality risk of a 1-s.d. increase in each annual temperature metric. Points and lines represent mortality effect estimates and their corresponding 95% CIs, respectively. Each row in the figure represents a separate regression based on the specification of Model 1. The baseline regression in the first row includes province-by-year fixed effects and province-by-month fixed effects. The more stringent fixed effect regression in the second row includes province-by-year fixed effects, province-by-month fixed effects and province-by-day fixed effects. The more relaxed fixed effect regressions in the third rows only includes province-by-year fixed effects. Standard errors in all regressions are clustered at the county level. More details are presented in Supplementary Text 6 and Table S10.

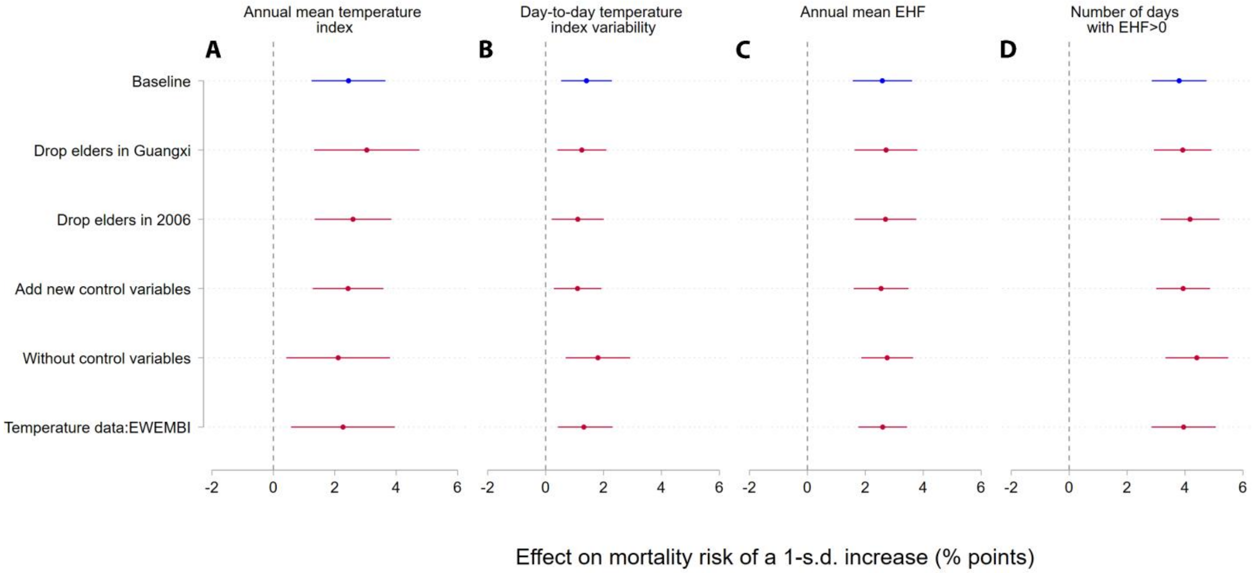

Fig. S8. Robustness tests for baseline regression. The robustness tests for the baseline of annual mean temperature index (**A**), day-to-day temperature index variability (**B**), annual mean EHF (**C**), and the number of days with EHF>0 (**D**). Points and lines represent mortality effect estimates and their corresponding 95% CIs, respectively. Each row in the figure represents a separate regression based on the specification of Model 1. The first row describes the baseline estimates. The second row excludes older adult samples from Guangxi province which has the highest number of deaths. The third row excludes older adult samples from the year with the highest number of deaths in 2006. The fourth row adds additional control variables (self-reported living conditions and self-reported health status). The fifth row does not include any control variables. The sixth row uses a different climate re-analysis datasets (EWEMBI). Province-by-year fixed effects and province-by-month fixed effects are included in each regression, and standard errors are clustered at the county level. More details are presented in Supplementary Text 8 and Table S12.

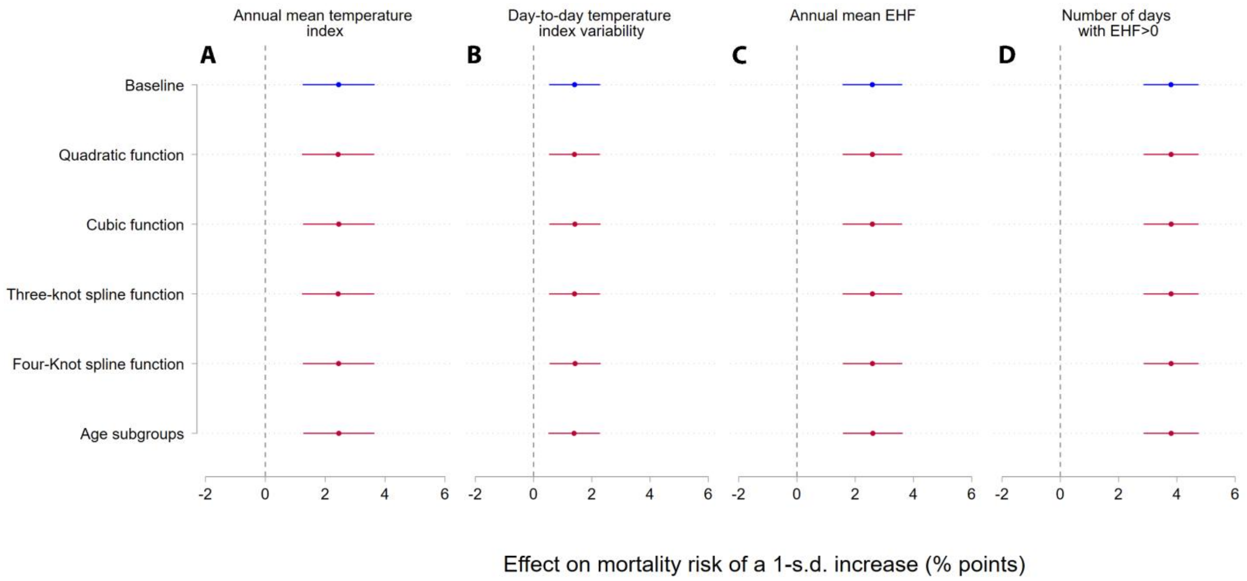

Fig. S9. Comparison of the regression specifications with different age-mortality functions for the effects of annual temperature metrics on mortality risk of older adults. Estimates of the effect on mortality risk of a 1-s.d. increase in each annual temperature metric. Points and lines represent mortality effect estimates and their corresponding 95% CIs, respectively. Each row represents a separate regression based on the specification of Model 1. The first row includes age in the regression model in the form of a linear function. The second row includes age in the regression model in the form of a quadratic function. The third row includes age in the regression model in the form of a cubic function. The fourth row includes age in the regression model in the form of a three-knot spline function. The fifth row includes age in the regression model in the form of a four-knot spline function. The sixth row includes age in the regression model in the form of a factor variable (4 different non-numeric levels). Province-by-year fixed effects and province-by-month fixed effects are included in each regression, and standard errors are clustered at the county level. More details are presented in Supplementary Text 9 and Table S13.

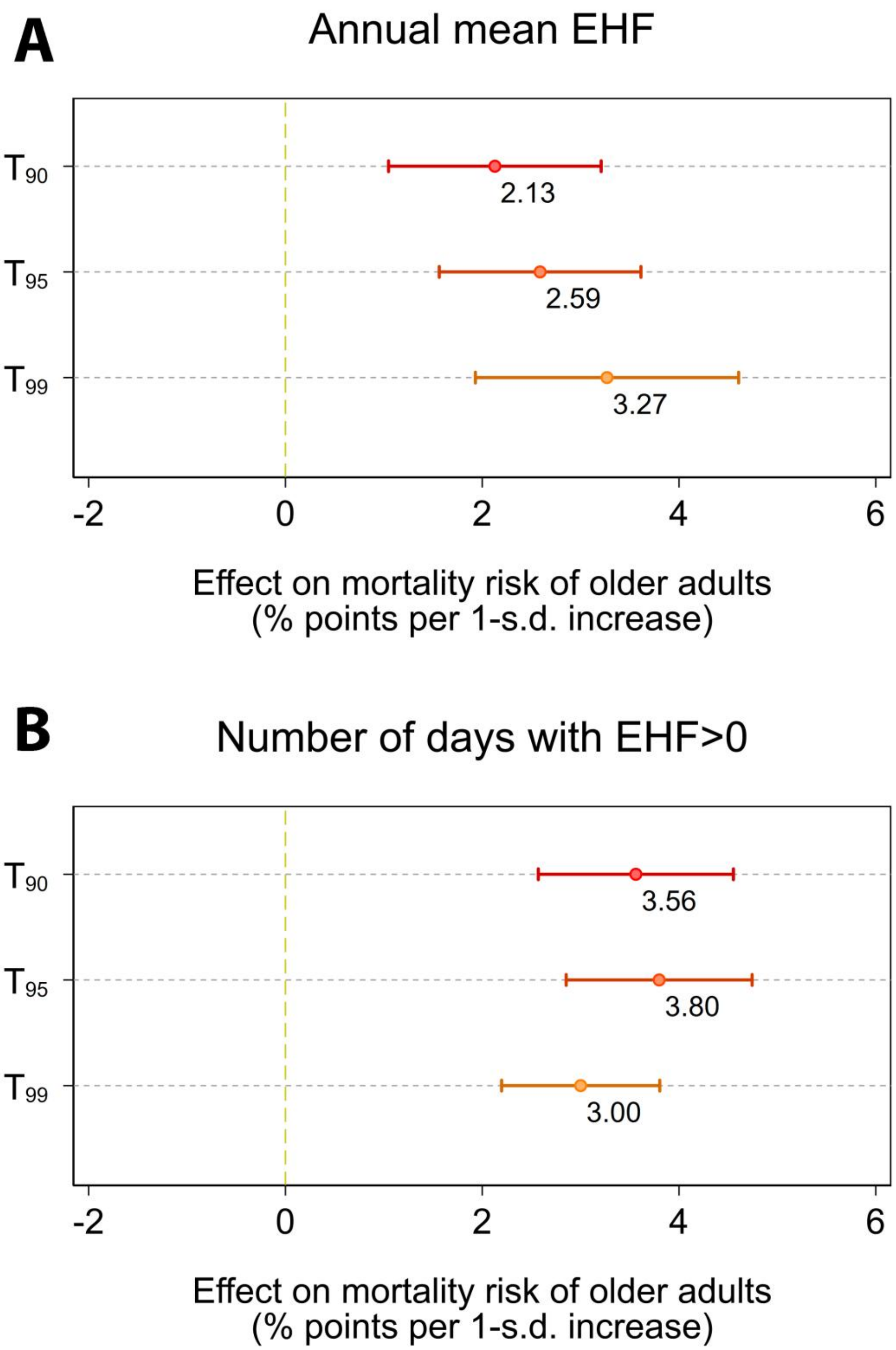

Fig. S10. Linear effects of extreme heat events of varying intensities (EHF using 90th, 95th and 99th percentiles as the thresholds) on mortality risk of older adults. Points and lines represent mortality effect estimates and their corresponding 95%CIs of a 1-s.d. increase in temperature metrics, respectively. Province-by-year fixed effects and province-by-month fixed effects are included in each regression based on the specification of Model 1, and standard errors are clustered at the county level. More details are presented in Table S18.

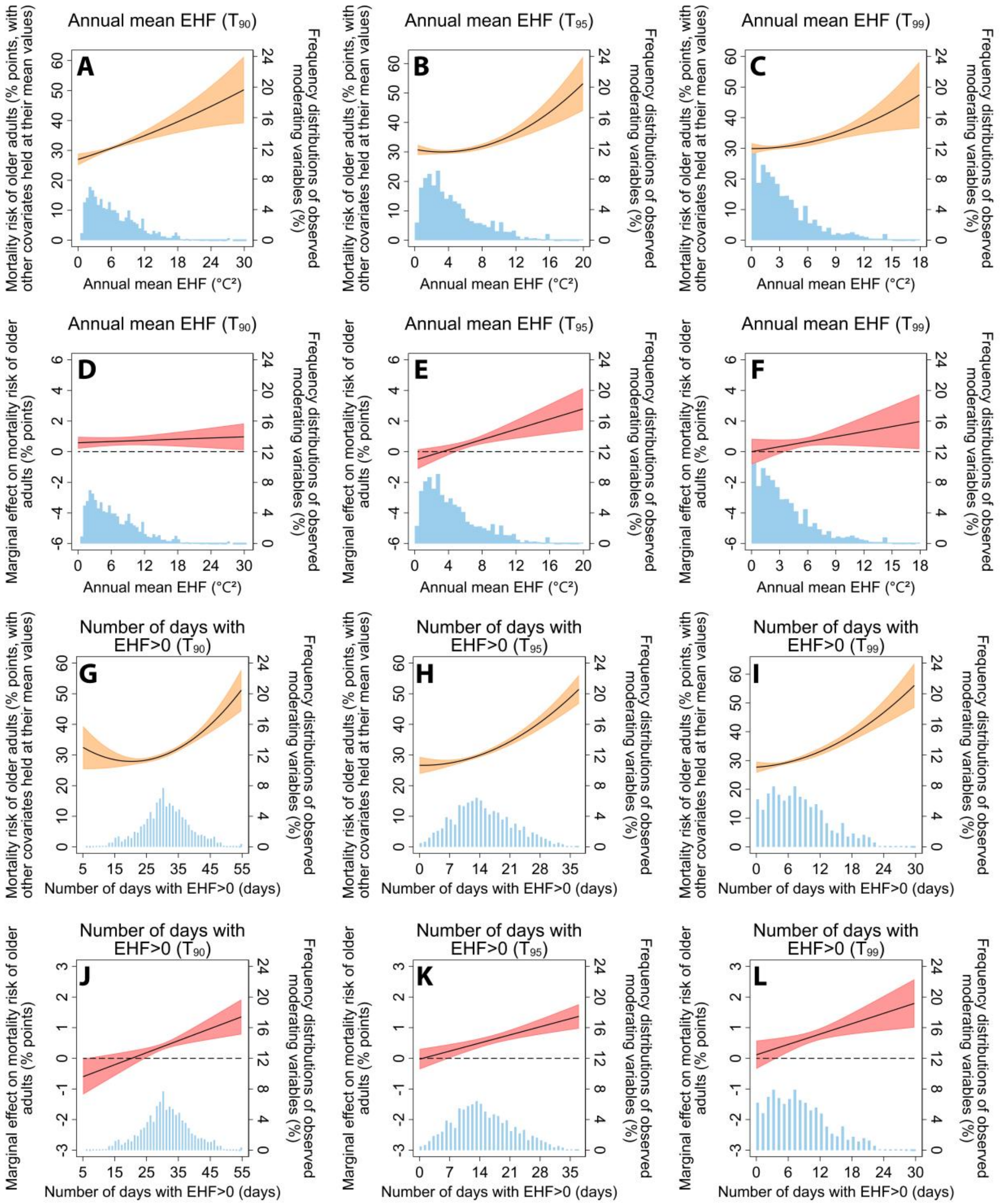

Fig. S11. Nonlinear effects of extreme heat events of varying intensities (EHF using 90th, 95th and 99th percentiles as the thresholds) on mortality risk of older adults. The relationships between annual mean EHF (**A**-**C**), number of days with EHF>0 (**G**-**I**), calculated using different thresholds, and mortality risk, with other covariates fixed at the sample means. The marginal effects in annual mean EHF (**D**-**F**) and number of days with EHF>0 (**J**-**L**), calculated using different thresholds, on mortality risk of older adults. The 95% CIs are shown in orange or red. The frequency distributions of observed moderating variables are shown as blue histograms. Province-by-year fixed effects and province-by-month fixed effects are included in each regression based on the specification of Model 3, and standard errors are clustered at the county level. More details are presented in Table S18.

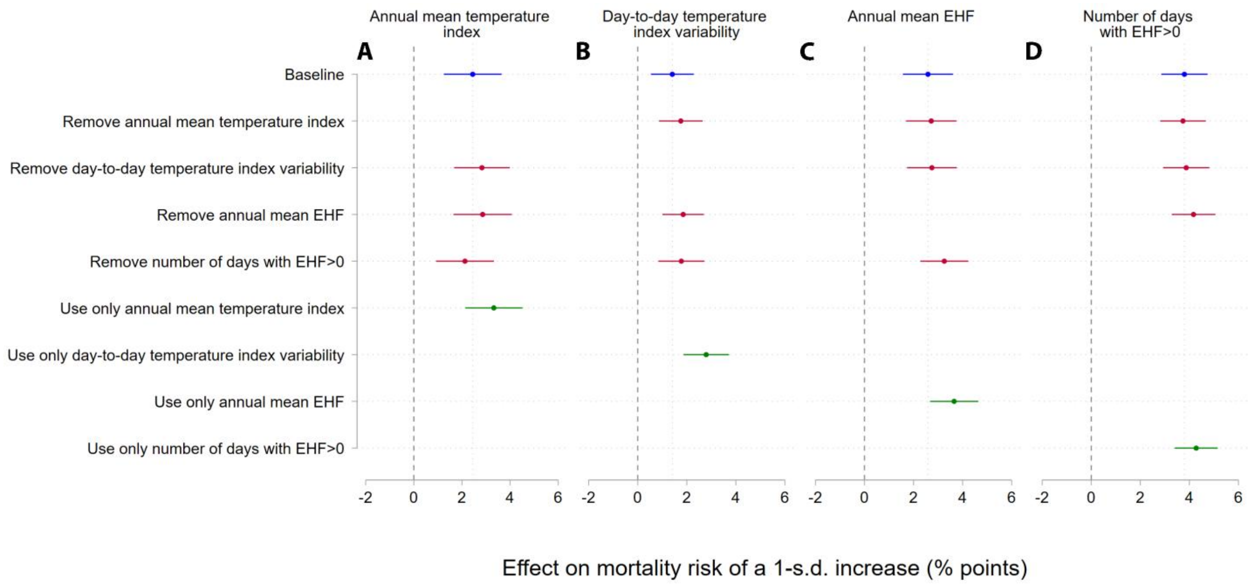

Fig. S12. The interdependence of the annual temperature metrics and the explanatory power of individual annual temperature metric. Estimates of the effect on mortality risk of a 1-s.d. increase in each annual temperature metric. Points and lines represent mortality effect estimates and their corresponding 95% CIs, respectively. Each row in the figure represents a separate regression based on the specification of Model 1. The first row describes the baseline estimates. The second through fifth rows each remove a specific annual temperature metric from the baseline regression model. The sixth through ninth rows individually evaluate each of the annual temperature metrics. Province-by-year fixed effects and province-by-month fixed effects are included in each regression, and standard errors are clustered at the county level. More details are presented in Tables S19-20.

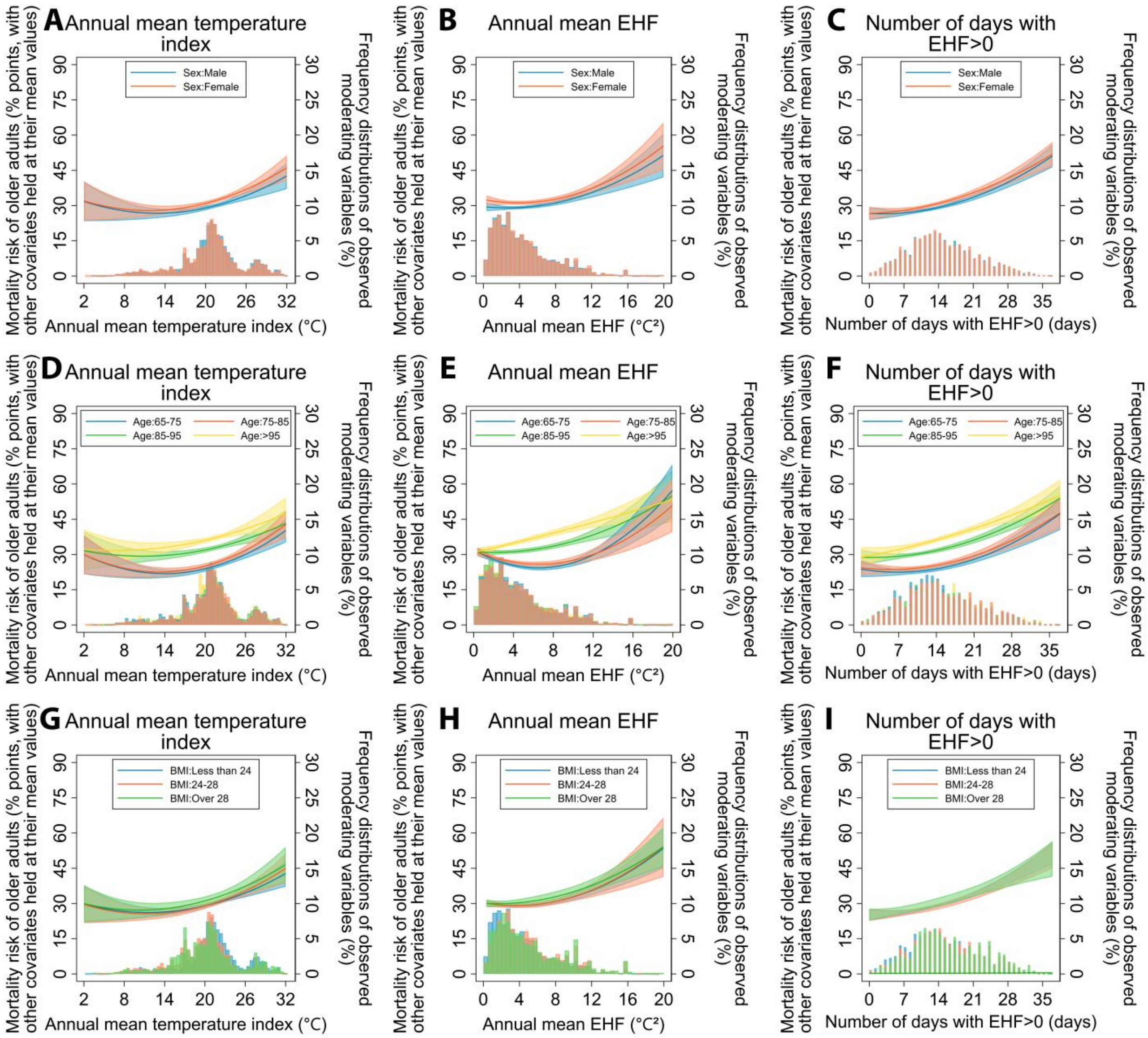

Fig. S13. Individual characteristics heterogeneity on the relationships between annual temperature metrics and mortality risk of older adults. A-C, The relationships between annual temperature metrics and mortality risk of different subgroups of older adults with different sex. **D-F**, The relationships between annual temperature metrics and mortality risk of different subgroups of older adults with different age. **G-I**, The relationships between annual temperature metrics and mortality risk of different subgroups of older adults with different BMIs. The colored shaded areas represent the 95%CIs. Province-by-year fixed effects and province-by-month fixed effects are included in each regression based on the specification of Model 6, and standard errors are clustered at the county level. The frequency distributions of observed moderating variables are shown as histograms.

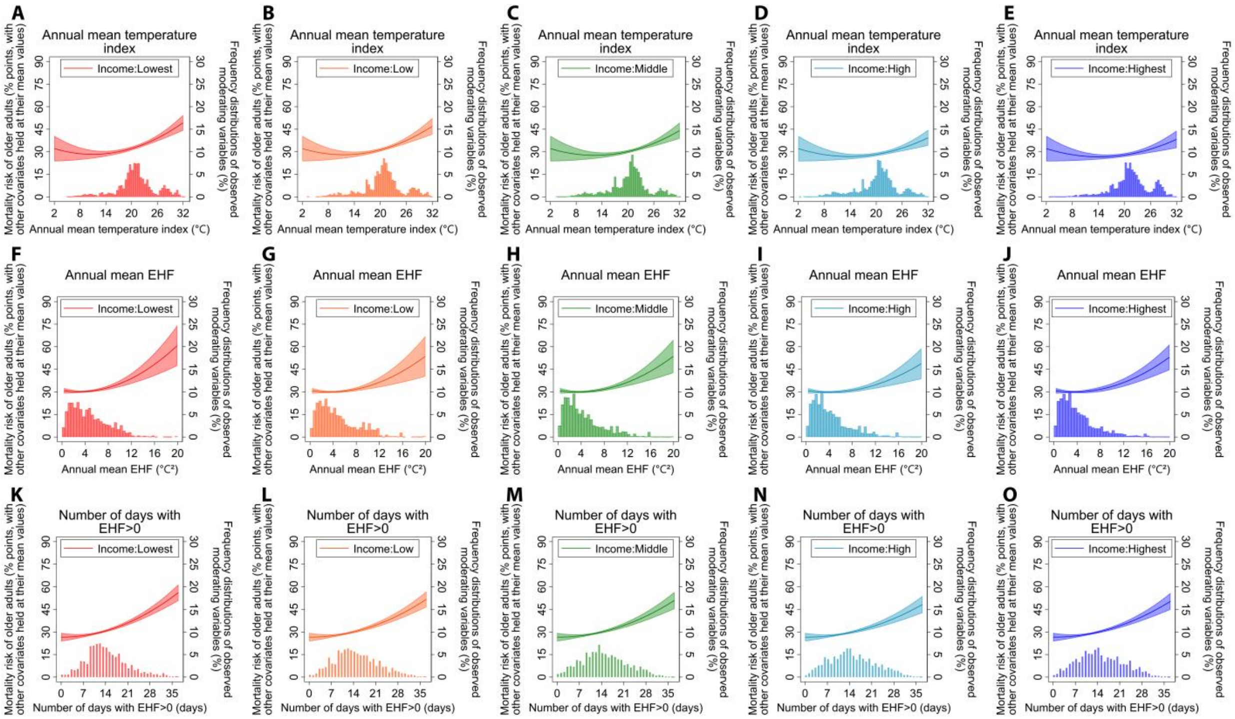

Fig. S14. Income heterogeneity on the relationships between annual temperature metrics and mortality risk of older adults. A-E, The relationships between annual mean temperature index and mortality risk of different subgroups of older adults with different household per capita annual income. **F-J**, The relationships between annual mean EHF and mortality risk of different subgroups of older adults with different household per capita annual income. **K-O**, The relationships between number of days with EHF > 0 and mortality risk of different subgroups of older adults with different household per capita annual income. Province-by-year fixed effects and province-by-month fixed effects are included in each regression based on the specification of Model 6, and standard errors are clustered at the county level. The frequency distributions of observed moderating variables are shown as histograms.

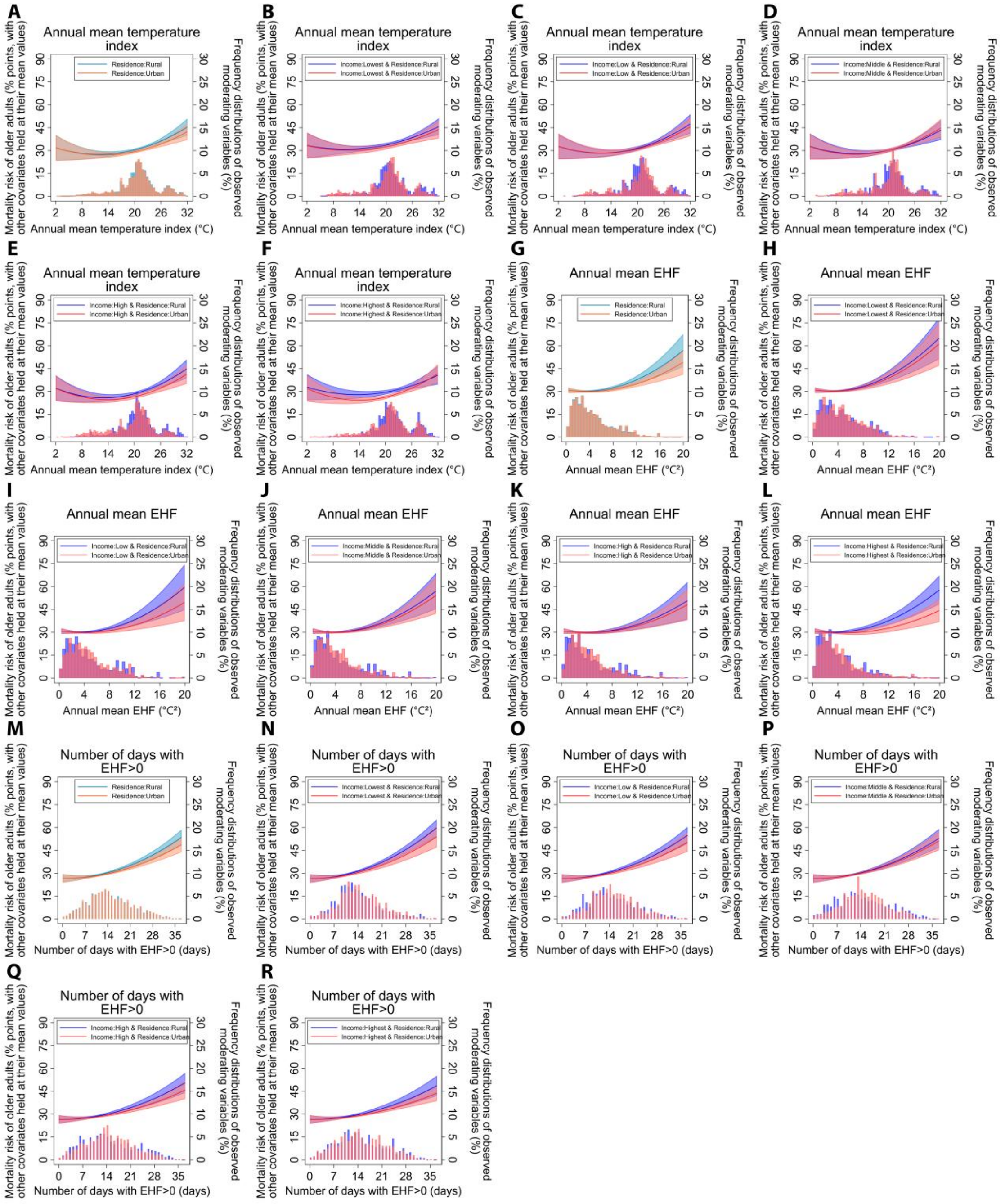

Fig. S15. Residence and income heterogeneity the relationships between annual temperature metrics and mortality risk of older adults. **A**, **G**, **M**, The relationships between annual temperature metrics and mortality risk of different subgroups of older adults with different urban-rural residences. **B**-**F**, **H**-**L**, **N**-**R**, The relationships between annual temperature metrics and mortality risk of different subgroups of older adults with different household per capita annual income and urban-rural residences. The colored shaded areas represent the 95%CIs. Province-by-year fixed effects and province-by-month fixed effects are included in each regression based on the specification of Model 6, and standard errors are clustered at the county level. The frequency distributions of observed moderating variables are shown as histograms.

Table S1. Statistics of the survey data (Individuals=27,233; Records=51,914).

Variable	Observation		Mean	Std. Dev.	Min	Max	Missing Obs.
Mortality status	51,914						0
Survival	36,046	(69.43%)					
Mortality	15,868	(30.57%)					
Sex	51,914						0
Man	29,233	(56.31%)					
Woman	22,681	(43.69%)					
Age	51,914		86.405	11.225	65	120	0
Group by age	51,914						0
65-75	9,620	(18.53%)					
75-85	12,475	(24.03%)					
85-95	16,110	(31.03%)					
Over 95	13,709	(26.41%)					
BMI	51,914						0
Less than 24	41,259	(79.48%)					
24-28	6,215	(11.97%)					
Over 28	4,440	(8.55%)					
Health rating	51,620						294
Very healthy	11,991	(23.23%)					
Relatively healthy	30,547	(59.18%)					
Weak	7,934	(15.37%)					
Very ill	1,148	(2.22%)					
Cohabitation situation	51,568						346
Living with family	42,177	(81.79%)					
Living alone	8,220	(15.94%)					
Living in institution	1,171	(2.27%)					
Marital status	51,555						359
Currently married, living with spouse	17,208	(33.38%)					
Separated	883	(1.71%)					
Divorced	168	(0.33%)					
Widowed	32,808	(63.64%)					
Never married	488	(0.95%)					
Whether they have children	50,072		0.969	0.171	0	1	1,842
Smoking status	51,574						340
Never smoked	34,397	(66.69%)					
Previously did not smoke but currently smoke	911	(1.77%)					
Smoked in the past	7,986	(15.48%)					
Consistently smoke	8,280	(16.05%)					
Drinking status	51,476						438
Never drank	35,646	(69.25%)					
Previously did not drink but currently drink	1,674	(3.25%)					
Drank in the past	6,751	(13.11%)					
Consistently drink	7,405	(14.39%)					
Exercise status	51,287						627
Never exercised	29,953	(58.40%)					
Previously did not exercise but currently exercise	5,841	(11.39%)					
Exercised in the past	6,146	(11.98%)					
Consistently exercise	9,347	(18.22%)					
Residence	51,914						0
Rural	29,213	(56.27%)					
Urban	22,701	(43.73%)					
Education years	51,660		2.169	3.473	0	25	254
Group by household per capita annual income	51,228						686
Lowest (0-2.2 thousand yuan per year)	9,897	(19.32%)					
Low (2.2-6 thousand yuan per year)	10,154	(19.82%)					
Middle (6-15 thousand yuan per year)	9,932	(19.39%)					
High (15-38 thousand yuan per year)	10,556	(20.61%)					
Highest (over 38 thousand yuan per year)	10,689	(20.87%)					
Staple food category	51,691						223
Rice and wheat	49,890	(96.52%)					
Coarse grain	1,801	(3.48%)					
Whether ot eat vegetables everyday	51,914		0.557	0.497	0	1	0
Whether ot eat fruits everyday	51,914		0.126	0.332	0	1	0
Whether ot eat pork everyday	51,914		0.339	0.473	0	1	0
Whether ot eat fish everyday	51,914		0.122	0.327	0	1	0
Whether ot eat egg everyday	51,914		0.365	0.482	0	1	0
Whether ot eat beans everyday	51,914		0.221	0.415	0	1	0
Whether ot eat pickle everyday	51,914		0.186	0.389	0	1	0
Whether ot eat tea everyday	51,914		0.258	0.437	0	1	0
Whether ot eat garlic everyday	51,914		0.187	0.389	0	1	0
Hypertension	49,588		0.242	0.428	0	1	2,326
Diabetes	49,244		0.036	0.186	0	1	2,670
Heart disease	49,431		0.107	0.309	0	1	2,483
Stroke and cerebrovascular disease	49,461		0.070	0.256	0	1	2,453
Bronchitis/emphysema/pneumonia and asthma	49,598		0.117	0.321	0	1	2,316
Tuberculosis	49,462		0.008	0.087	0	1	2,452
Cataracts	49,383		0.123	0.329	0	1	2,531
Glaucoma	46,934		0.022	0.146	0	1	4,980
Cancer	48,874		0.007	0.079	0	1	3,040
Gastrointestinal ulcers	46,919		0.051	0.219	0	1	4,995
Parkinson's disease	48,287		0.006	0.079	0	1	3,627
Pressure ulcers	47,123		0.007	0.081	0	1	4,791
Arthritis	49,720		0.176	0.381	0	1	2,194
Dementia	49,785		0.027	0.163	0	1	2,129

Table S2. Effects of the four annual temperature metrics based on each of eight daily temperature metrics on mortality risk of older adults.

Variables	Mortality risk of older adults							
**Daily temperature metric**	**Daily mean temperature**	**Daily max temperature**	**Daily min temperature**	**Average daily temperature**	**Daily mean temperature index**	**Daily max temperature index**	**Daily min temperature index**	**Average daily temperature index**
Annual mean temperature index	0.00642***	0.00606***	0.00656***	0.00650***	0.00529***	0.00409***	0.00540***	0.00523***
	[0.00184]	[0.00176]	[0.00172]	[0.00181]	[0.00154]	[0.00154]	[0.00147]	[0.00148]
Day-to-day temperature index variability	0.03117***	0.00924	0.03563***	0.03076***	0.03760***	0.01782**	0.03687***	0.03575***
	[0.00954]	[0.00753]	[0.01058]	[0.00942]	[0.00939]	[0.00720]	[0.01037]	[0.00926]
Annual mean EHF	0.01108***	0.00703***	0.01199***	0.01055***	0.00833***	0.00315***	0.00914***	0.00767***
	[0.00215]	[0.00169]	[0.00298]	[0.00222]	[0.00143]	[0.00085]	[0.00260]	[0.00156]
Number of days with EHF>0	0.00367***	0.00274***	0.00434***	0.00324***	0.00513***	0.00403***	0.00509***	0.00546***
	[0.00078]	[0.00077]	[0.00070]	[0.00074]	[0.00070]	[0.00071]	[0.00068]	[0.00069]
Annual total precipitation	0.00590***	0.00553***	0.00574***	0.00578***	0.00564***	0.00475***	0.00542***	0.00571***
	[0.00136]	[0.00132]	[0.00133]	[0.00135]	[0.00135]	[0.00134]	[0.00140]	[0.00137]
Province-by-year FE	YES	YES	YES	YES	YES	YES	YES	YES
Province-by-month FE	YES	YES	YES	YES	YES	YES	YES	YES
Control variables	YES	YES	YES	YES	YES	YES	YES	YES
Observations	41246	41246	41246	41246	41246	41246	41246	41246
Adjusted R^2	0.723	0.722	0.723	0.722	0.724	0.722	0.724	0.724
Within R^2	0.0914	0.0890	0.0915	0.0895	0.0963	0.0906	0.0944	0.0966
AIC	134.511	240.9584	128.8673	219.8073	-89.6198	167.4945	-4.689631	-102.0264
BIC	678.0315	784.4789	672.3878	763.3278	453.9007	711.015	538.8309	441.4941

Notes: Each column in the table represents a separate fixed-effects regression based on the specification of Model 1, where the explanatory variables include four different annual temperature metrics derived from a specific daily temperature metric. The section above the dashed line shows the regression results for the main explanatory variables, while the section below the dashed line presents the parameters of the regression model. The bolded parts indicate the varying parameters. The numbers show the regression coefficients of each annual temperature metric on mortality risk of older adults. Standard errors are clustered at the county level and shown in parentheses. Within R^2 represents the proportion of the variation in the dependent variable explained by the independent variables, excluding the contribution of the fixed effects. AIC and BIC refer to the Akaike Information Criterion and the Bayesian Information Criterion, respectively. *p<0.1, **p<0.05, ***p<0.01.

Table S3. Effects of the four warm-season temperature metrics based on each of eight daily temperature metrics on mortality risk of older adults.

Variables	Mortality risk of older adults							
**Daily temperature metric**	**Daily mean temperature**	**Daily max temperature**	**Daily min temperature**	**Average daily temperature**	**Daily mean temperature index**	**Daily max temperature index**	**Daily min temperature index**	**Average daily temperature index**
Warm-season mean temperature index	0.00447***	0.00296**	0.00653***	0.00488***	0.00385***	0.00234*	0.00603***	0.00390***
	[0.00164]	[0.00144]	[0.00169]	[0.00161]	[0.00145]	[0.00124]	[0.00150]	[0.00139]
Day-to-day temperature index variability	0.02211***	0.01214*	0.02891***	0.02492***	0.02750***	0.01864***	0.03172***	0.02893***
	[0.00836]	[0.00651]	[0.00939]	[0.00812]	[0.00776]	[0.00586]	[0.00868]	[0.00773]
Warm-season mean EHF	0.00979***	0.00608***	0.00957***	0.00897***	0.00867***	0.00342***	0.00827***	0.00813***
	[0.00203]	[0.00152]	[0.00270]	[0.00213]	[0.00139]	[0.00079]	[0.00233]	[0.00156]
Number of days with EHF>0	0.00358***	0.00238***	0.00440***	0.00317***	0.00529***	0.00399***	0.00517***	0.00553***
	[0.00072]	[0.00073]	[0.00065]	[0.00070]	[0.00062]	[0.00067]	[0.00064]	[0.00061]
Annual total precipitation	0.00557***	0.00483***	0.00569***	0.00549***	0.00538***	0.00436***	0.00556***	0.00538***
	[0.00138]	[0.00133]	[0.00135]	[0.00137]	[0.00140]	[0.00134]	[0.00142]	[0.00140]
Province-by-year FE	YES	YES	YES	YES	YES	YES	YES	YES
Province-by-month FE	YES	YES	YES	YES	YES	YES	YES	YES
Control variables	YES	YES	YES	YES	YES	YES	YES	YES
Observations	41246	41246	41246	41246	41246	41246	41246	41246
Adjusted R^2	0.723	0.722	0.723	0.722	0.725	0.722	0.724	0.724
Within R^2	0.0910	0.0877	0.0925	0.0894	0.0966	0.0927	0.0965	0.0966
AIC	151.9682	301.0978	84.88081	224.0368	-99.4788	71.70053	-99.23041	-101.1216
BIC	695.4887	844.6183	628.4013	767.5573	444.0417	615.221	444.2901	442.3989

Notes: Each column in the table represents a separate fixed-effects regression based on the specification of Model 1, where the explanatory variables include four different warm-season temperature metrics derived from a specific daily temperature metric. The section above the dashed line shows the regression results for the main explanatory variables, while the section below the dashed line presents the parameters of the regression model. The bolded parts indicate the varying parameters. The numbers show the regression coefficients of each warm-season temperature metric on mortality risk of older adults. Standard errors are clustered at the county level and shown in parentheses. Within R^2 represents the proportion of the variation in the dependent variable explained by the independent variables, excluding the contribution of the fixed effects. AIC and BIC refer to the Akaike Information Criterion and the Bayesian Information Criterion, respectively. *p<0.1, **p<0.05, ***p<0.01.

Table S4. The Pearson correlation coefficients between the same types of annual temperature metrics derived from eight different daily temperature metrics.

**Daily temperature metric used to calculate the annual temperature metric**		Daily mean temperature	Daily max temperature	Daily min temperature	Average daily temperature	Daily mean temperature index	Daily max temperature index	Daily min temperature index	Average daily temperature index
		**Annual mean temperature index**							
Daily mean temperature	**Annual mean temperature index**	1.0000							
Daily max temperature		0.9822	1.0000						
Daily min temperature		0.9910	0.9493	1.0000					
Average daily temperature		0.9997	0.9848	0.9895	1.0000				
Daily mean temperature index		0.9989	0.9748	0.9947	0.9983	1.0000			
Daily max temperature index		0.9878	0.9959	0.9624	0.9901	0.9844	1.0000		
Daily min temperature index		0.9872	0.9410	0.9996	0.9854	0.9921	0.9561	1.0000	
Average daily temperature index		0.9987	0.9776	0.9933	0.9988	0.9996	0.9872	0.9905	1.0000
		**Day-to-day temperature index variability**							
Daily mean temperature	**Day-to-day temperature index variability**	1.0000							
Daily max temperature		0.9073	1.0000						
Daily min temperature		0.9218	0.7185	1.0000					
Average daily temperature		0.9959	0.9142	0.9250	1.0000				
Daily mean temperature index		0.9668	0.8095	0.9381	0.9638	1.0000			
Daily max temperature index		0.8275	0.8988	0.6506	0.8412	0.8194	1.0000		
Daily min temperature index		0.8789	0.6487	0.9912	0.8832	0.9235	0.6101	1.0000	
Average daily temperature index		0.9562	0.8085	0.9373	0.9624	0.9945	0.8338	0.9253	1.0000
		**Annual mean EHF**							
Daily mean temperature	**Annual mean EHF**	1.0000							
Daily max temperature		0.8164	1.0000						
Daily min temperature		0.7292	0.5079	1.0000					
Average daily temperature		0.9508	0.8022	0.7606	1.0000				
Daily mean temperature index		0.6212	0.3644	0.6609	0.5883	1.0000			
Daily max temperature index		0.5703	0.4272	0.4525	0.5826	0.8419	1.0000		
Daily min temperature index		0.5039	0.3097	0.8312	0.5229	0.7994	0.5534	1.0000	
Average daily temperature index		0.5803	0.3658	0.7006	0.5949	0.9558	0.8390	0.8403	1.0000
		**Number of days with EHF > 0**							
Daily mean temperature	**Number of days with EHF > 0**	1.0000							
Daily max temperature		0.8371	1.0000						
Daily min temperature		0.8396	0.6853	1.0000					
Average daily temperature		0.9646	0.8886	0.8476	1.0000				
Daily mean temperature index		0.8600	0.6965	0.8796	0.8394	1.0000			
Daily max temperature index		0.8341	0.7543	0.7817	0.8441	0.8796	1.0000		
Daily min temperature index		0.7438	0.5807	0.9276	0.7478	0.8451	0.7400	1.0000	
Average daily temperature index		0.8252	0.6782	0.8946	0.8300	0.9474	0.8899	0.8713	1.0000

Notes: The table presents four sections from top to bottom, showing the Pearson correlation coefficients among the eight annual mean temperature indices, the eight day-to-day temperature index variabilities, the eight annual mean EHF, and the eight numbers of days with EHF > 0, all derived from the eight daily temperature metrics. Due to the fact that different daily temperature metrics capture temperature conditions at different times of the day, the same type of annual temperature metric derived from different daily temperature metrics may also vary. Their correlation coefficients can reflect the degree of similarity between them.

Table S5. Testing for collinearity among the same type of annual temperature metrics derived from eight different daily temperature metrics.

Daily temperature metric used to calculate the annual temperature metric	VIF			
	Annual mean temperature index	Day-to-day temperature index variability	Annual mean EHF	Number of days with EHF > 0
Daily mean temperature	135053.87	2281.64	18.1	18.19
Daily max temperature	$-2.57e^{13}$	121.41	3.89	5.6
Daily min temperature	$-3.71e^{13}$	567.12	8.12	12.9
Average daily temperature	$-1.21e^{14}$	2379.7	15.11	20.43
Daily mean temperature index	128722.44	1804.52	19.32	13.26
Daily max temperature index	2935.65	38.53	5.66	6.84
Daily min temperature index	12186.77	421.78	8.66	8.4
Average daily temperature index	121772.87	1733.61	21.81	15.34

Notes: Each column in the table represents a separate test for collinearity, examining the same type of annual temperature metric derived from eight daily temperature metrics. VIF stands for Variance Inflation Factor, and if VIF<10, it indicates that there is no collinearity among the variables.

Table S6. Testing for collinearity among the four annual temperature metrics based on each of eight daily temperature metrics.

Annual temperature metric	VIF							
	Daily mean temperature	Daily max temperature	Daily min temperature	Average daily temperature	Daily mean temperature index	Daily max temperature index	Daily min temperature index	Average daily temperature index
Annual mean temperature index	1.49	1.11	2.06	1.46	1.39	1.03	1.94	1.35
Day-to-day temperature index variability	1.66	1.21	2.14	1.61	1.53	1.05	2.03	1.44
Annual mean EHF	1.38	1.3	1.4	1.36	1.28	1.21	1.32	1.28
Number of days with EHF>0	1.18	1.14	1.12	1.14	1.22	1.2	1.14	1.2

Notes: Each column in the table represents a separate test for collinearity, examining the four annual temperature metrics derived from each of eight daily temperature metrics. VIF stands for Variance Inflation Factor, and if VIF < 10, it indicates that there is no collinearity among the variables.

Table S7. The Akaike Information Criterion (AIC), Bayesian Information Criterion (BIC), Precision, Recall, and F1 for regressions incorporating annual temperature metrics.

Annual mean temperature index	Day-to-day temperature index variability	Annual mean EHF	Number of days with EHF>0	AIC	BIC	Precision	Recall	F1
DTM1 ***	DTM1 ***	DTM1 ***	DTM1 ***	134.511	678.0315	43.5269	50.3505	46.6784
DTM2 ***	DTM1 **	DTM1 ***	DTM1 ***	133.941	677.4615	43.3101	49.827	46.3297
DTM3 ***	DTM1 ***	DTM1 ***	DTM1 ***	140.6345	684.155	43.7049	50.2751	46.7494
DTM4 ***	DTM1 ***	DTM1 ***	DTM1 ***	134.4268	677.9473	43.504	50.2934	46.6409
DTM5 ***	DTM1 ***	DTM1 ***	DTM1 ***	128.3964	671.9169	43.4935	50.5843	46.7591
DTM6 ***	DTM1 **	DTM1 ***	DTM1 ***	123.5064	667.0269	43.2302	50.1549	46.4249
DTM7 ***	DTM1 ***	DTM1 ***	DTM1 ***	138.961	682.4815	43.7658	50.4575	46.8631
DTM8 ***	DTM1 ***	DTM1 ***	DTM1 ***	127.6833	671.2038	43.4315	50.5145	46.6938
DTM2&DTM3	DTM1 **	DTM1 ***	DTM1 ***	135.4112	687.559	43.3274	50.0166	46.4205
DTM6&DTM7	DTM1 **	DTM1 ***	DTM1 ***	125.3863	677.5341	43.2382	50.1179	46.4128
DTM6 ***	DTM1 **	DTM1 ***	DTM1 ***	123.5064	667.0269	43.2302	50.1549	46.4249
DTM6 ***	DTM2 *	DTM1 ***	DTM1 ***	133.0567	676.5772	43.0012	50.0307	46.2375
DTM6 ***	DTM3 **	DTM1 ***	DTM1 ***	128.9373	672.4578	43.0886	49.9339	46.2478
DTM6 ***	DTM4 **	DTM1 ***	DTM1 ***	126.2557	669.7762	43.1989	50.1356	46.3987
DTM6 ***	DTM5 ***	DTM1 ***	DTM1 ***	104.5609	648.0814	43.6983	50.7222	46.9379
DTM6 ***	DTM6 **	DTM1 ***	DTM1 ***	123.5049	667.0254	43.3073	50.2812	46.5227
DTM6 ***	DTM7 **	DTM1 ***	DTM1 ***	121.2324	664.7529	43.1769	50.0094	46.3312
DTM6 ***	DTM8 ***	DTM1 ***	DTM1 ***	108.2395	651.76	43.6465	50.6276	46.8674
DTM6 ***	DTM2&DTM3	DTM1 ***	DTM1 ***	130.56	682.7078	43.0934	49.9716	46.2669
DTM6 **	DTM6&DTM7	DTM1 ***	DTM1 ***	120.1009	672.2487	43.3408	50.1807	46.4989
DTM6 ***	DTM5 ***	DTM1 ***	DTM1 ***	104.5609	648.0814	43.6983	50.7222	46.9379
DTM6 ***	DTM5 ***	DTM2 ***	DTM2 ***	190.3595	733.88	42.8985	49.1448	45.7994
DTM6 ***	DTM5 ***	DTM3 ***	DTM3 ***	82.95674	626.4772	44.3089	51.4269	47.5918
DTM6 ***	DTM5 ***	DTM4 ***	DTM4 ***	185.1552	728.6757	43.6066	50.0612	46.5989
DTM6 ***	DTM5 ***	DTM5 ***	DTM5 ***	-97.67111	445.8494	44.1459	51.9796	47.7339
DTM6 ***	DTM5 ***	DTM6 ***	DTM6 ***	141.2513	684.7718	43.3765	49.3071	46.1422
DTM6 ***	DTM5 ***	DTM7 ***	DTM7 ***	-46.91967	496.6008	44.2842	51.9047	47.7824
DTM6 ***	DTM5 ***	DTM8 ***	DTM8 ***	-111.0884	432.4321	44.5244	51.9784	47.9529
DTM6 ***	DTM5 ***	DTM2***&DTM3*	DTM2&DTM3**	-28.42622	532.3489	44.2528	51.7276	47.6902
DTM6 ***	DTM5 ***	DTM6***&DTM7**	DTM6&DTM7**	-24.5135	536.2616	44.2523	51.7277	47.6901

Notes: Each row in the table represents a separate fixed-effects regression based on the specification of Model 1, where the explanatory variables include four annual temperature metrics derived from the specific daily temperature metrics (DTM). DTM1: daily mean temperature; DTM2: daily max temperature; DTM3: daily min temperature; DTM4: average daily temperature; DTM5: daily mean temperature index; DTM6: daily max temperature index; DTM7: daily min temperature index; DTM8: average daily temperature index. Since the Pearson correlation coefficients and collinearity between annual temperature metrics derived from daily max temperature and daily min temperature are relatively low (Table S4-S5), both can be included in the regression simultaneously. Additionally, since the annual mean EHF and the number of days with EHF > 0 represent two different characteristics of extreme heat events, the daily temperature metrics used to calculate these two annual temperature metrics should be the same in the same regression model. The precision, recall, and F1 score for each regression are obtained as the average values from five-fold cross-validation. Within each set of experiments, the combination with the lowest AIC and BIC is highlighted in red font, and an asterisk (*) following daily temperature metrics indicates the significance of the derived annual temperature metrics in the regression. *p<0.1, **p<0.05, ***p<0.01.

Table S8. Comparison of estimates of the effect on mortality risk using the linear probability model (LPM) and logistic regression.

Variables	Mortality risk of older adults					
Annual mean temperature index	0.00410**	0.02471**	0.00524***	0.05987***	0.00437***	0.06037***
	[0.00204]	[0.01242]	[0.00115]	[0.01518]	[0.00117]	[0.01928]
Marginal effects at the sample means		0.00467**		0.00571***		0.00534***
		[0.00233]		[0.00148]		[0.00175]
Day-to-day temperature index variability	0.10739***	0.63256***	0.05264***	0.51546***	0.05442***	0.37835*
	[0.01444]	[0.09090]	[0.01845]	[0.19079]	[0.01776]	[0.22327]
Marginal effects at the sample means		0.11941***		0.04915***		0.03347*
		[0.01667]		[0.01800]		[0.01941]
Annual mean EHF	0.00125	0.01054	0.00393**	0.04665**	0.00501***	0.05770**
	[0.00240]	[0.01354]	[0.00169]	[0.01967]	[0.00183]	[0.02620]
Marginal effects at the sample means		0.00199		0.00445**		0.00511**
		[0.00255]		[0.00180]		[0.00228]
Number of days with EHF>0	0.00948***	0.05654***	0.00513***	0.06384***	0.00502***	0.05826***
	[0.00127]	[0.00752]	[0.00080]	[0.00805]	[0.00081]	[0.00983]
Marginal effects at the sample means		0.01067***		0.00609***		0.00515***
		[0.00143]		[0.00092]		[0.00095]
Annual total precipitation	0.00589***	0.03499***	0.00663***	0.08448***	0.00384**	0.03757*
	[0.00167]	[0.00992]	[0.00140]	[0.01728]	[0.00150]	[0.02210]
**Regression model**	**LPM**	**Logistic**	**LPM**	**Logistic**	**LPM**	**Logistic**
**Province FE**	**NO**	**NO**	**YES**	**YES**	**YES**	**YES**
**Year FE**	**NO**	**NO**	**YES**	**YES**	**YES**	**YES**
**Month FE**	**NO**	**NO**	**NO**	**NO**	**YES**	**YES**
Control variables	YES	YES	YES	YES	YES	YES
Observations	41251	41251	41251	35006	41251	35006
Adjusted R^2	0.235	0.215	0.640	0.423	0.680	0.534

Notes: Each column in the table represents a separate regression. The section above the dashed line shows the regression results for the main explanatory variables, while the section below the dashed line presents the parameters of the regression model, with the bolded parts indicating the varying parameters. The numbers show the regression coefficients of each annual temperature metric on mortality risk of older adults. For the logistic regression model, we compute the marginal effects at the sample means of each explanatory variable, and compare them with the regression coefficients from the LPM. Standard errors are clustered at the county level and shown in parentheses. *p<0.1, **p<0.05, ***p<0.01.

Table S9. Predicted mortality probability of older adults using the Linear Probability Model.

	Predicted mortality probability			
Mean value	0.31141	0.31141	0.31141	0.31133
Standard deviation	0.22504	0.12066	0.10721	0.10162
Minimum value	-0.38299	-0.08228	-0.05811	-0.05781
Maximum value	1.25806	0.88878	0.79939	0.81213
Proportion of predictions outside interval	8.66%	0.19%	0.04%	0.03%
**Province FE**	**NO**	**YES**	**YES**	**NO**
**Year FE**	**NO**	**YES**	**YES**	**NO**
**Month FE**	**NO**	**NO**	**YES**	**NO**
**Province-by-Year FE**	**NO**	**NO**	**NO**	**YES**
**Province-by-Month FE**	**NO**	**NO**	**NO**	**YES**
Control variables	YES	YES	YES	YES
Obs.	41,251	41,251	41,251	41,251

Notes: Each column in the table represents a separate linear probability regression based on the specification of Model 1. The section below the dashed line presents the parameters of the regression model, with the bolded parts indicating the varying parameters. The numbers show the statistical descriptions of the elderly mortality probabilities predicted by each linear probability regression. The fourth column represents the baseline regression model of this study.

Table S10. Comparison of the regression specifications with different fixed effects for the effects of annual temperature metrics on mortality risk of older adults.

Variables	Mortality risk of older adults		
Annual mean temperature index	0.00519***	0.00495***	0.00592***
	[0.00130]	[0.00133]	[0.00118]
Day-to-day temperature index variability	0.03061***	0.03657***	0.04279***
	[0.00968]	[0.01035]	[0.01178]
Annual mean EHF	0.00767***	0.00739***	0.00688***
	[0.00155]	[0.00159]	[0.00172]
Number of days with EHF>0	0.00546***	0.00544***	0.00641***
	[0.00069]	[0.00071]	[0.00073]
Annual total precipitation	0.00586***	0.00589***	0.00783***
	[0.00135]	[0.00137]	[0.00139]
**Province-by-Year FE**	**YES**	**YES**	**YES**
**Province-by-Month FE**	**YES**	**YES**	**NO**
**Province-by-Day FE**	**NO**	**YES**	**NO**
Control variables	YES	YES	YES
Observations	41246	41245	41246
Adjusted R^2	0.724	0.728	0.664
Within R^2	0.0968	0.0946	0.120

Notes: Each column in the table represents a separate fixed-effects regression based on the specification of Model 1. The first column represents the baseline regression model. The section above the dashed line shows the regression results for the main explanatory variables, while the section below the dashed line presents the parameters of the regression model, with the bolded parts indicating the varying parameters. The numbers show the regression coefficients of each annual temperature metric on mortality risk of older adults. Standard errors are clustered at the county level and shown in parentheses. Within R^2 represents the proportion of the variation in the dependent variable explained by the independent variables, excluding the contribution of the fixed effects. *p<0.1, **p<0.05, ***p<0.01.

Table S11. Comparison between balanced and unbalanced panel regressions and the adjustment of standard errors.

Variables	Mortality risk of older adults							
Annual mean temperature index	0.00463**	0.00474***	0.00520***	0.00519***	0.00519***	0.00519**	0.00519**	0.00519**
	[0.00174]	[0.00118]	[0.00157]	[0.00130]	[0.00084]	[0.00203]	[0.00183]	[0.00191]
Day-to-day temperature index variability	0.02786**	0.02863**	0.03252***	0.03061***	0.03061***	0.03061**	0.03061**	0.03061**
	[0.01017]	[0.01002]	[0.01237]	[0.00968]	[0.00640]	[0.01211]	[0.01052]	[0.01108]
Annual mean EHF	0.00591***	0.00579***	0.00685***	0.00767***	0.00767***	0.00767***	0.00767**	0.00767**
	[0.00144]	[0.00106]	[0.00182]	[0.00155]	[0.00073]	[0.00213]	[0.00259]	[0.00247]
Number of days with EHF>0	0.00421***	0.00471***	0.00643***	0.00546***	0.00546***	0.00546***	0.00546**	0.00546**
	[0.00056]	[0.00057]	[0.00082]	[0.00069]	[0.00034]	[0.00096]	[0.00203]	[0.00224]
Annual total precipitation	0.00388***	0.00610***	0.00666***	0.00586***	0.00586***	0.00586***	0.00586**	0.00586***
	[0.00114]	[0.00104]	[0.00163]	[0.00135]	[0.00056]	[0.00194]	[0.00252]	[0.00127]
Province-by-Year FE	YES	YES	YES	YES	YES	YES	YES	YES
Province-by-Month FE	YES	YES	YES	YES	YES	YES	YES	YES
Control variables	YES	YES	YES	YES	YES	YES	YES	YES
**Standard errors clustered**	**Robust**	**Robust**	**County**	**County**	**Individual**	**Province**	**Year**	**Month**
**Samples**	**2nd**	**4th**	**All**	**All**	**All**	**All**	**All**	**All**
**Weight**	/	/	**1/waves**	/	/	/	/	/
Observations	11023	9930	41246	41246	41246	41246	41246	41246
Adjusted R^2	0.783	0.782	0.704	0.724	0.724	0.724	0.724	0.724
Within R^2	0.0511	0.0426	0.102	0.0968	0.0968	0.0968	0.0968	0.0968

Notes: Each column in the table represents a separate fixed-effects regression based on the specification of Model 1. The fourth column represents the baseline regression model. The section above the dashed line shows the regression results for the main explanatory variables, while the section below the dashed line presents the parameters of the regression model, with the bolded parts indicating the varying parameters. The numbers show the regression coefficients of each annual temperature metric on mortality risk of older adults. Standard errors of different columns are clustered at different levels and shown in parentheses. Within R^2 represents the proportion of the variation in the dependent variable explained by the independent variables, excluding the contribution of the fixed effects. *p<0.1, **p<0.05, ***p<0.01.

Table S12. Robustness check of the baseline regression.

Variables	Mortality risk of older adults					
Annual mean temperature index	0.00519***	0.00639***	0.00549***	0.00516***	0.00446**	0.00505**
	[0.00130]	[0.00184]	[0.00135]	[0.00125]	[0.00181]	[0.00192]
Day-to-day temperature index variability	0.03061***	0.02878***	0.02411**	0.02402***	0.03956***	0.02810**
	[0.00968]	[0.00992]	[0.00994]	[0.00914]	[0.01243]	[0.01024]
Annual mean EHF	0.00767***	0.00790***	0.00795***	0.00755***	0.00796***	0.00809***
	[0.00155]	[0.00161]	[0.00160]	[0.00143]	[0.00132]	[0.00133]
Number of days with EHF>0	0.00546***	0.00571***	0.00601***	0.00568***	0.00629***	0.00599***
	[0.00069]	[0.00074]	[0.00075]	[0.00068]	[0.00079]	[0.00086]
Annual total precipitation	0.00586***	0.00534***	0.00610***	0.00569***	0.00329**	0.00566***
	[0.00135]	[0.00151]	[0.00143]	[0.00124]	[0.00148]	[0.00126]
Province-by-Year FE	YES	YES	YES	YES	YES	YES
Province-by-Month FE	YES	YES	YES	YES	YES	YES
**Control variables**	**YES**	**YES**	**YES**	**YES**	**NO**	**YES**
**Additional CV**	**NO**	**NO**	**NO**	**YES**	**NO**	**NO**
**Excluding**	**/**	**Guangxi**	**2006**	**/**	**/**	**/**
**Meteorological data**	**ERA5**	**ERA5**	**ERA5**	**ERA5**	**ERA5**	**EWEMBI**
Observations	41246	37405	39650	37499	51912	38636
Adjusted R^2	0.724	0.724	0.704	0.732	0.677	0.719
Within R^2	0.0968	0.101	0.100	0.0862	0.0195	0.0922

Notes: Each column in the table represents a separate fixed-effects regression based on the specification of Model 1. The section above the dashed line shows the regression results for the main explanatory variables, while the section below the dashed line presents the parameters of the regression model, with the bolded parts indicating the varying parameters. The numbers show the regression coefficients of each annual temperature metric on mortality risk of older adults. The first column describes the baseline estimates. The second column excludes older adult samples from Guangxi province with the highest number of deaths. The third column excludes older adult samples from the year with the highest number of deaths in 2006. The fourth column adds additional control variables (self-reported living conditions and self-reported health status). The fifth column does not include any control variables. The sixth column uses a different climate re-analysis datasets (EWEMBI). Standard errors are clustered at the county level and shown in parentheses. Within R^2 represents the proportion of the variation in the dependent variable explained by the independent variables, excluding the contribution of the fixed effects. *p<0.1, **p<0.05, ***p<0.01.

Table S13. Comparison of the regression specifications with different age-mortality functions for the effects of annual temperature metrics on mortality risk of older adults.

Variables	Mortality risk of older adults					
Annual mean temperature index	0.00519***	0.00516***	0.00520***	0.00516***	0.00519***	0.00521***
	[0.00130]	[0.00130]	[0.00129]	[0.00131]	[0.00129]	[0.00128]
Day-to-day temperature index variability	0.03061***	0.03050***	0.03084***	0.03055***	0.03095***	0.03022***
	[0.00968]	[0.00967]	[0.00970]	[0.00967]	[0.00967]	[0.00978]
Annual mean EHF	0.00767***	0.00767***	0.00767***	0.00767***	0.00768***	0.00771***
	[0.00155]	[0.00154]	[0.00154]	[0.00154]	[0.00154]	[0.00155]
Number of days with EHF>0	0.00546***	0.00547***	0.00547***	0.00547***	0.00547***	0.00547***
	[0.00069]	[0.00069]	[0.00069]	[0.00069]	[0.00069]	[0.00069]
Annual total precipitation	0.00586***	0.00586***	0.00585***	0.00587***	0.00584***	0.00585***
	[0.00135]	[0.00135]	[0.00135]	[0.00135]	[0.00134]	[0.00135]
Age	0.00428***	-0.00160	-0.13902***			
	[0.00020]	[0.00196]	[0.02222]			
Age*Age		0.00003***	0.00164***			
		[0.00001]	[0.00026]			
Age*Age*Age			-0.00001***			
			[0.00000]			
Age_spline1				0.00348***	-0.00007	
				[0.00028]	[0.00043]	
Age_spline2				0.00101***	0.01308***	
				[0.00039]	[0.00146]	
Age_spline3					-0.04130***	
					[0.00511]	
Age_group [75-85]						0.01325***
						[0.00292]
Age_group [85-95]						0.07562***
						[0.00400]
Age_group [>95]						0.11535***
						[0.00564]
Province-by-Year FE	YES	YES	YES	YES	YES	YES
Province-by-Month FE	YES	YES	YES	YES	YES	YES
Control variables	YES	YES	YES	YES	YES	YES
**Functional form**	**Linear**	**Quadratic**	**Cubic**	**Three-knot spline**	**Four-Knot spline**	**Age subgroups**
Observations	41246	41246	41246	41246	41246	41246
Adjusted R^2	0.724	0.724	0.725	0.724	0.725	0.724
Within R^2	0.0968	0.0970	0.0982	0.0970	0.0986	0.0957

Notes: Each column in the table represents a separate fixed-effects regression based on the specification of Model 1. The section above the dashed line shows the regression results for the main explanatory variables, while the section below the dashed line presents the parameters of the regression model, with the bolded parts indicating the varying parameters. The numbers show the regression coefficients of each annual temperature metric on mortality risk of older adults. The first column includes age in the regression model in the form of a linear function (baseline). The second column includes age in the regression model in the form of a quadratic function. The third column includes age in the regression model in the form of a cubic function. The fourth column includes age in the regression model in the form of a three-knot spline function. The fifth column includes age in the regression model in the form of a four-knot spline function. The sixth column includes age in the regression model in the form of a factor variable (4 different non-numeric levels, with the 65-75 age subgroup as the reference group). Standard errors are clustered at the county level and shown in parentheses. Within R^2 represents the proportion of the variation in the dependent variable explained by the independent variables, excluding the contribution of the fixed effects. *p<0.1, **p<0.05, ***p<0.01.

Table S14. Full regression results of the baseline regression specification for the effects of annual temperature metrics on mortality risk of older adults.

**Annual mean temperature index**	**Day-to-day temperature index variability**	**Annual mean EHF**	**Number of days with EHF>0**	**Annual total precipitation**	**Sex_woman**
0.00519***	0.03061***	0.00767***	0.00546***	0.00586***	0.02154***
[0.00130]	[0.00968]	[0.00155]	[0.00069]	[0.00135]	[0.00327]
**Age**	**Ethnicity_Hui**	**Ethnicity_Zhuang**	**Ethnicity_Yao**	**Ethnicity_Korea**	**Ethnicity_Man**
0.00428***	0.01546	0.02233**	-0.00474	-0.03605	-0.01606
[0.00020]	[0.01734]	[0.01093]	[0.02066]	[0.06470]	[0.01883]
**Ethnicity_Mongolia**	**Ethnicity_Others**	**BMI_24-28**	**BMI_>28**	**Health rating_2**	**Health rating_3**
0.04476	-0.04373	-0.00616	0.01989***	0.02100***	0.08338***
[0.04662]	[0.02819]	[0.00748]	[0.00382]	[0.00318]	[0.00564]
**Health rating_4**	**Cohabitation_2**	**Cohabitation_3**	**Marital_2**	**Marital_3**	**Marital_4**
0.13916***	0.02274***	0.00342	0.01784*	-0.02266	0.02814***
[0.01326]	[0.00358]	[0.01056]	[0.00967]	[0.01792]	[0.00341]
**Marital_5**	**Children_have**	**Smoke_2**	**Smoke_3**	**Smoke_4**	**Drink_2**
0.02688	-0.00439	-0.00487	0.00204	0.01670***	0.00001
[0.02174]	[0.00917]	[0.00794]	[0.00358]	[0.00401]	[0.00660]
**Drink_3**	**Drink_4**	**Exercise_2**	**Exercise_3**	**Exercise_4**	**Residence_urban**
0.00780*	-0.00264	0.01263***	-0.02098***	-0.01741***	-0.01615***
[0.00445]	[0.00396]	[0.00464]	[0.00420]	[0.00366]	[0.00353]
**Education years**	**Income**	**Staple food_1**	**Vegetables_1**	**Fruits_1**	**Pork_1**
-0.00117***	-0.00338***	0.00833	-0.00182	-0.01221**	-0.00258
[0.00042]	[0.00056]	[0.00748]	[0.00326]	[0.00488]	[0.00338]
**Fish_1**	**Egg_1**	**Bean_1**	**Pickle_1**	**Tea_1**	**Garlic_1**
0.00692	-0.00878***	0.00419	0.00086	0.00249	0.00301
[0.00434]	[0.00329]	[0.00371]	[0.00318]	[0.00313]	[0.00312]
**Hypertension_1**	**Diabetes_1**	**Heart disease_1**	**Stroke_1**	**Bronchitis_1**	**Tuberculosis_1**
0.00798**	0.00653	-0.00181	0.00156	0.00599	-0.01211
[0.00349]	[0.00690]	[0.00442]	[0.00485]	[0.00431]	[0.01542]
**Cataracts_1**	**Glaucoma_1**	**Cancer_1**	**Gastrointestinal_1**	**Parkinson_1**	**Pressure ulcers_1**
-0.00521	-0.00821	0.08197***	0.01227**	-0.00196	0.02079
[0.00439]	[0.00951]	[0.02001]	[0.00589]	[0.01949]	[0.01978]
**Arthritis_1**	**Dementia**				
-0.00567	0.04273***				
[0.00366]	[0.01167]				
Province-by-Year FE				Yes	
Province-by-Month FE				Yes	
Control variables				Yes	
Observations				41246	
Adjusted R^2				0.724	
Within R^2				0.0968	

Notes: The table represents the full results of baseline regression. The section above the dashed line shows the regression results for the independent variables, while the section below the dashed line presents the parameters of the regression model. The numbers show the regression coefficients of each independent variable on mortality risk of older adults. The detailed description of each factor variable is presented in Supplementary Text 3. Standard errors are clustered at the county level and shown in parentheses. Within R^2 represents the proportion of the variation in the dependent variable explained by the independent variables, excluding the contribution of the fixed effects. *p<0.1, **p<0.05, ***p<0.01.

Table S15. Testing for collinearity among independent variables of baseline regression.

**Independent variable**	Annual mean temperature index	Day-to-day temperature index variability	Annual mean EHF	Number of days with EHF>0
**VIF**	1.94	1.43	1.47	1.26
**Independent variable**	Annual total precipitation	Sex	.Age	Ethnicity
**VIF**	2.06	1.74	1.59	1.06
**Independent variable**	BMI	Health rating	Cohabitation	Marital
**VIF**	1.07	1.22	1.14	1.66
**Independent variable**	Children	Smoke	Drink	Exercise
**VIF**	1.03	1.49	1.3	1.19
**Independent variable**	Residence	Education years	Income	Staple food
**VIF**	1.18	1.44	1.08	1.03
**Independent variable**	Vegetables	Fruits	Pork	Fish
**VIF**	1.13	1.21	1.26	1.23
**Independent variable**	Egg	Bean	Pickle	Tea
**VIF**	1.33	1.23	1.07	1.11
**Independent variable**	Garlic	Hypertension	Diabetes	Heart disease
**VIF**	1.09	1.12	1.07	1.14
**Independent variable**	Stroke	Bronchitis	Tuberculosis	Cataracts
**VIF**	1.09	1.04	1.02	1.08
**Independent variable**	Glaucoma	Cancer	Gastrointestinal	Parkinson
**VIF**	1.03	1.01	1.02	1.01
**Independent variable**	Pressure ulcers	Arthritis	Dementia	
**VIF**	1.02	1.06	1.05	

Notes: The table represents the test for collinearity among independent variables of baseline regression. VIF stands for Variance Inflation Factor, and if VIF < 10, it indicates that there is no collinearity among the variables.

Table S16. The quadratic effects of annual temperature metrics on mortality risk of older adults.

Variables	Mortality risk of older adults	
Annual mean temperature index	-0.01098**	-0.01062*
	[0.00544]	[0.00553]
Annual mean temperature index^2	0.00044***	0.00043***
	[0.00014]	[0.00015]
Day-to-day temperature index variability	0.06395	0.03939***
	[0.07713]	[0.00969]
Day-to-day temperature index variability^2	-0.00379	
	[0.01185]	
Annual mean EHF	-0.00492	-0.00491
	[0.00328]	[0.00327]
Annual mean EHF^2	0.00084***	0.00084***
	[0.00025]	[0.00025]
Number of days with EHF>0	-0.00029	-0.00028
	[0.00171]	[0.00171]
Number of days with EHF>0^2	0.00019***	0.00019***
	[0.00005]	[0.00005]
Annual total precipitation	0.00542***	0.00543***
	[0.00134]	[0.00134]
Province-by-Year FE	YES	YES
Province-by-Month FE	YES	YES
Control variables	YES	YES
Observations	41246	41246
Adjusted R^2	0.726	0.726
Within R^2	0.101	0.101

Notes: The first column in the table represents a fixed-effects regression based on the specification of Model 2, and the second column presents a fixed-effects regression based on the specification of Model 3. The section above the dashed line shows the regression results for the main explanatory variables, while the section below the dashed line presents the parameters of the regression model. The numbers show the regression coefficients of each annual temperature metric on mortality risk of older adults. The first column includes both the linear and quadratic terms of the four annual temperature metrics. Since the quadratic effect of the day-to-day temperature Index variability is insignificant, we retain the quadratic terms for the other three annual temperature metrics and perform a linear fit for the day-to-day temperature index variability (second column). Standard errors are clustered at the county level and shown in parentheses. Within R^2 represents the proportion of the variation in the dependent variable explained by the independent variables, excluding the contribution of the fixed effects. *p<0.1, **p<0.05, ***p<0.01.

Table S17. The interactive effects of the annual temperature metrics on mortality risk of older adults.

Variables	Mortality risk of older adults				
Annual mean temperature index	0.00170	0.00599***	0.00472**	0.00557***	0.00510***
	[0.00467]	[0.00169]	[0.00197]	[0.00136]	[0.00130]
Annual mean temperature index*Day-to-day temperature index variability	0.00124				
	[0.00151]				
Annual mean temperature index*Annual mean EHF		-0.00025			
		[0.00024]			
Annual mean temperature index*Number of days with EHF>0			0.00004		
			[0.00010]		
Day-to-day temperature index variability	0.00772	0.03093***	0.03041***	0.03043***	0.03012***
	[0.03048]	[0.00978]	[0.00979]	[0.00979]	[0.00971]
Day-to-day temperature index variability*Annual mean EHF				0.00035	
				[0.00060]	
Day-to-day temperature index variability*Number of days with EHF>0					0.00028
					[0.00036]
Annual mean EHF	0.00769***	0.01246***	0.00766***	0.00665***	0.00779***
	[0.00155]	[0.00441]	[0.00155]	[0.00139]	[0.00153]
Number of days with EHF>0	0.00544***	0.00551***	0.00472**	0.00555***	0.00496***
	[0.00070]	[0.00071]	[0.00225]	[0.00071]	[0.00098]
Annual total precipitation	0.00581***	0.00591***	0.00588***	0.00586***	0.00573***
	[0.00137]	[0.00136]	[0.00134]	[0.00135]	[0.00135]
Province-by-Year FE	YES	YES	YES	YES	YES
Province-by-Month FE	YES	YES	YES	YES	YES
Control variables	YES	YES	YES	YES	YES
Observations	41246	41246	41246	41246	41246
Adjusted R^2	0.724	0.724	0.724	0.724	0.724
Within R^2	0.0968	0.0968	0.0968	0.0968	0.0968

Notes: Each column in the table represents a separate fixed-effects regression based on the specification of Model 4. The section above the dashed line shows the regression results for the main explanatory variables, while the section below the dashed line presents the parameters of the regression model. The numbers show the regression coefficients of each annual temperature metric on mortality risk of older adults. Standard errors are clustered at the county level and shown in parentheses. Within R^2 represents the proportion of the variation in the dependent variable explained by the independent variables, excluding the contribution of the fixed effects. *p<0.1, **p<0.05, ***p<0.01.

Table S18. The effects of extreme heat events of varying intensities (EHF using 90th, 95th and 99th percentiles as the thresholds) on mortality risk of older adults.

Variables	Mortality risk of older adults					
Annual mean temperature index	0.00513***	0.00519***	0.00475***	-0.01102**	-0.01062*	-0.01156**
	[0.00132]	[0.00130]	[0.00123]	[0.00553]	[0.00553]	[0.00558]
Annual mean temperature index^2				0.00042***	0.00043***	0.00043***
				[0.00015]	[0.00015]	[0.00015]
Day-to-day temperature index variability	0.03482***	0.03061***	0.03356***	0.03957***	0.03939***	0.04120***
	[0.01065]	[0.00968]	[0.00971]	[0.01071]	[0.00969]	[0.00997]
Annual mean EHF (T_{90})	0.00730***			0.00584***		
	[0.00152]			[0.00200]		
Annual mean EHF^2 (T_{90})				0.00006		
				[0.00009]		
Number of days with EHF>0 (T_{90})	0.00409***			-0.00799**		
	[0.00056]			[0.00357]		
Number of days with EHF>0^2 (T_{90})				0.00020***		
				[0.00006]		
Annual mean EHF (T_{95})		0.00767***			-0.00491	
		[0.00155]			[0.00327]	
Annual mean EHF^2 (T_{95})					0.00084***	
					[0.00025]	
Number of days with EHF>0 (T_{95})		0.00546***			-0.00028	
		[0.00069]			[0.00171]	
Number of days with EHF>0^2 (T_{95})					0.00019***	
					[0.00005]	
Annual mean EHF (T_{99})			0.00674***			-0.00003
			[0.00174]			[0.00430]
Annual mean EHF^2 (T_{99})						0.00095**
						[0.00032]
Number of days with EHF>0 (T_{99})			0.00647***			0.00115
			[0.00092]			[0.00236]
Number of days with EHF>0^2 (T_{99})						0.00028***
						[0.00010]
Annual total precipitation	0.00570***	0.00586***	0.00530***	0.00541***	0.00543***	0.00493***
	[0.00139]	[0.00135]	[0.00129]	[0.00137]	[0.00134]	[0.00128]
Province-by-Year FE	YES	YES	YES	YES	YES	YES
Province-by-Month FE	YES	YES	YES	YES	YES	YES
Control variables	YES	YES	YES	YES	YES	YES
Observations	41246	41246	41246	41246	41246	41246
Adjusted R^2	0.724	0.724	0.725	0.724	0.725	0.724
Within R^2	0.0968	0.0970	0.0982	0.0970	0.0986	0.0957

Notes: The first to third columns in the table represent a fixed-effects regression based on the specification of Model 1, and the fourth to sixth columns present a fixed-effects regression based on the specification of Model 3. The section above the dashed line shows the regression results for the main explanatory variables, while the section below the dashed line presents the parameters of the regression model. The numbers show the regression coefficients of each annual temperature metric on mortality risk of older adults. The first to third columns fit the linear effects of EHF identified using the 90th, 95th, and 99th thresholds, respectively, while the fourth to sixth columns fit the quadratic effects of EHF at each threshold. Standard errors are clustered at the county level and shown in parentheses. Within R^2 represents the proportion of the variation in the dependent variable explained by the independent variables, excluding the contribution of the fixed effects. *p<0.1, **p<0.05, ***p<0.01.

Table S19. The interdependence of the annual temperature metrics.

Variables	Mortality risk of older adults				
Annual mean temperature index	0.00519***		0.00600***	0.00607***	0.00451***
	[0.00130]		[0.00125]	[0.00131]	[0.00130]
Day-to-day temperature index variability	0.03061***	0.03816***		0.04022***	0.03861***
	[0.00968]	[0.00984]		[0.00935]	[0.01036]
Annual mean EHF	0.00767***	0.00806***	0.00813***		0.00964***
	[0.00155]	[0.00155]	[0.00153]		[0.00148]
Number of days with EHF>0	0.00546***	0.00538***	0.00557***	0.00600***	
	[0.00069]	[0.00068]	[0.00069]	[0.00065]	
Annual total precipitation	0.00586***	0.00467***	0.00596***	0.00568***	0.00474***
	[0.00135]	[0.00126]	[0.00134]	[0.00136]	[0.00131]
Province-by-Year FE	YES	YES	YES	YES	YES
Province-by-Month FE	YES	YES	YES	YES	YES
Control variables	YES	YES	YES	YES	YES
Observations	41246	41246	41246	41246	41246
Adjusted R^2	0.724	0.724	0.724	0.723	0.722
Within R^2	0.0968	0.0958	0.0961	0.0936	0.0885

Notes: Each column in the table represents a separate fixed-effects regression based on the specification of Model 1. The first column represents the baseline regression model. The section above the dashed line shows the regression results for the main explanatory variables, while the section below the dashed line presents the parameters of the regression model. The numbers show the regression coefficients of each annual temperature metric on mortality risk of older adults. Standard errors are clustered at the county level and shown in parentheses. Within R^2 represents the proportion of the variation in the dependent variable explained by the independent variables, excluding the contribution of the fixed effects. *p<0.1, **p<0.05, ***p<0.01.

Table S20. The explanatory power of individual annual temperature metric.

Variables	Mortality risk of older adults				
Annual mean temperature index	0.00519***	0.00705***			
	[0.00130]	[0.00129]			
Day-to-day temperature index variability	0.03061***		0.06058***		
	[0.00968]		[0.01026]		
Annual mean EHF	0.00767***			0.01082***	
	[0.00155]			[0.00148]	
Number of days with EHF>0	0.00546***				0.00615***
	[0.00069]				[0.00064]
Annual total precipitation	0.00586***	0.00446***	0.00307***	0.00356***	0.00406***
	[0.00135]	[0.00129]	[0.00119]	[0.00122]	[0.00125]
Province-by-Year FE	YES	YES	YES	YES	YES
Province-by-Month FE	YES	YES	YES	YES	YES
Control variables	YES	YES	YES	YES	YES
Observations	41246	41246	41246	41246	41246
Adjusted R^2	0.724	0.720	0.720	0.721	0.722
Within R^2	0.0968	0.0815	0.0822	0.0863	0.0906

Notes: Each column in the table represents a separate fixed-effects regression based on the specification of Model 1. The first column represents the baseline regression model. The section above the dashed line shows the regression results for the main explanatory variables, while the section below the dashed line presents the parameters of the regression model. The numbers show the regression coefficients of each annual temperature metric on mortality risk of older adults. Standard errors are clustered at the county level and shown in parentheses. Within R^2 represents the proportion of the variation in the dependent variable explained by the independent variables, excluding the contribution of the fixed effects. *p<0.1, **p<0.05, ***p<0.01.

Table S21. The individual characteristics heterogeneous impacts of annual mean temperature index on mortality risk of older adults.

Variables	Mortality risk of older adults		
Annual mean temperature index	0.00473***	0.00244*	0.00520***
	[0.00130]	[0.00129]	[0.00125]
Annual mean temperature index*Sex [Woman]	0.00103***		
	[0.00015]		
Annual mean temperature index*Age_group [75-85]		0.00061***	
		[0.00014]	
Annual mean temperature index*Age_group [85-95]		0.00329***	
		[0.00021]	
Annual mean temperature index*Age_group [>85]		0.00514***	
		[0.00029]	
Annual mean temperature index*BMI [24-28]			0.00007
			[0.00021]
Annual mean temperature index*BMI [>28]			0.00112***
			[0.00043]
Day-to-day temperature index variability	0.03065***	0.02824***	0.02966***
	[0.00969]	[0.01007]	[0.00931]
Annual mean EHF	0.00767***	0.00769***	0.00775***
	[0.00155]	[0.00155]	[0.00155]
Number of days with EHF>0	0.00546***	0.00547***	0.00574***
	[0.00069]	[0.00069]	[0.00064]
Annual total precipitation	0.00586***	0.00585***	0.00630***
	[0.00135]	[0.00136]	[0.00115]
Province-by-Year FE	YES	YES	YES
Province-by-Month FE	YES	YES	YES
Control variables	YES	YES	YES
Observations	41246	41246	41246
Adjusted R^2	0.724	0.723	0.723
Within R^2	0.0968	0.0938	0.0968

Notes: Each column in the table represents a separate fixed-effects regression with interaction terms based on the specification of Model 5. The numbers show the regression coefficients of each annual temperature metric on mortality risk of older adults. We interact individual characteristics of older adults (sex, age and BMI) with annual mean temperature index to assess the heterogeneity effects in annual mean temperature index across different individual characteristics. The interaction terms represent the difference in the effects of the annual mean temperature index between the subgroup and the reference group. For the sex subgroup, the reference group is "man"; for the age subgroups, the reference group is "65-75 years old"; and for BMI, the reference group is "BMI < 24". Standard errors are clustered at the county level and shown in parentheses. Within R^2 represents the proportion of the variation in the dependent variable explained by the independent variables, excluding the contribution of the fixed effects. *p<0.1, **p<0.05, ***p<0.01.

Table S22. The individual characteristics heterogeneous impacts of day-to-day temperature index variability on mortality risk of older adults.

Variables	Mortality risk of older adults		
Annual mean temperature index	0.00520***	0.00510***	0.00524***
	[0.00130]	[0.00129]	[0.00125]
Day-to-day temperature index variability	0.02769***	0.01127	0.02943***
	[0.00970]	[0.00991]	[0.00933]
Day-to-day temperature index variability*Sex [Woman]	0.00650***		
	[0.00102]		
Day-to-day temperature index variability*Age_group [75-85]		0.00424***	
		[0.00092]	
Day-to-day temperature index variability*Age_group [85-95]		0.02360***	
		[0.00123]	
Day-to-day temperature index variability*Age_group [>85]		0.03575***	
		[0.00172]	
Day-to-day temperature index variability*BMI [24-28]			0.00001
			[0.00131]
Day-to-day temperature index variability*BMI [>28]			0.00568**
			[0.00252]
Annual mean EHF	0.00767***	0.00768***	0.00776***
	[0.00155]	[0.00155]	[0.00155]
Number of days with EHF>0	0.00547***	0.00545***	0.00574***
	[0.00069]	[0.00069]	[0.00064]
Annual total precipitation	0.00586***	0.00582***	0.00630***
	[0.00135]	[0.00136]	[0.00115]
Province-by-Year FE	YES	YES	YES
Province-by-Month FE	YES	YES	YES
Control variables	YES	YES	YES
Observations	41246	41246	41246
Adjusted R^2	0.724	0.724	0.724
Within R^2	0.0967	0.0953	0.0967

Notes: Each column in the table represents a separate fixed-effects regression with interaction terms based on the specification of Model 5. The numbers show the regression coefficients of each annual temperature metric on mortality risk of older adults. We interact individual characteristics of older adults (sex, age and BMI) with day-to-day temperature index variability to assess the heterogeneity effects in day-to-day temperature index variability across different individual characteristics. The interaction terms represent the difference in the effects of the day-to-day temperature index variability between the subgroup and the reference group. For the sex subgroup, the reference group is "man"; for the age subgroups, the reference group is "65-75 years old"; and for BMI, the reference group is "BMI < 24". Standard errors are clustered at the county level and shown in parentheses. Within R^2 represents the proportion of the variation in the dependent variable explained by the independent variables, excluding the contribution of the fixed effects. *p<0.1, **p<0.05, ***p<0.01.

Table S23. The individual characteristics heterogeneous impacts of annual mean EHF on mortality risk of older adults.

Variables	Mortality risk of older adults		
Annual mean temperature index	0.00524***	0.00517***	0.00527***
	[0.00130]	[0.00128]	[0.00125]
Day-to-day temperature index variability	0.03023***	0.03043***	0.02983***
	[0.00972]	[0.00978]	[0.00928]
Annual mean EHF	0.00678***	0.00627***	0.00761***
	[0.00159]	[0.00136]	[0.00157]
Annual mean EHF*Sex [Woman]	0.00209***		
	[0.00052]		
Annual mean EHF*Age_group [75-85]		0.00035	
		[0.00099]	
Annual mean EHF*Age_group [85-95]		0.00106	
		[0.00132]	
Annual mean EHF*Age_group [>85]		0.00346**	
		[0.00140]	
Annual mean EHF*BMI [24-28]			0.00028
			[0.00078]
Annual mean EHF*BMI [>28]			0.00242*
			[0.00124]
Number of days with EHF>0	0.00546***	0.00545***	0.00574***
	[0.00069]	[0.00070]	[0.00064]
Annual total precipitation	0.00586***	0.00583***	0.00630***
	[0.00136]	[0.00134]	[0.00114]
Province-by-Year FE	YES	YES	YES
Province-by-Month FE	YES	YES	YES
Control variables	YES	YES	YES
Observations	41246	41246	41246
Adjusted R^2	0.724	0.724	0.724
Within R^2	0.0962	0.0960	0.0962

Notes: Each column in the table represents a separate fixed-effects regression with interaction terms based on the specification of Model 5. The numbers show the regression coefficients of each annual temperature metric on mortality risk of older adults. We interact individual characteristics of older adults (sex, age and BMI) with annual mean EHF to assess the heterogeneity effects in annual mean EHF across different individual characteristics. The interaction terms represent the difference in the effects of the annual mean EHF between the subgroup and the reference group. For the sex subgroup, the reference group is "man"; for the age subgroups, the reference group is "65-75 years old"; and for BMI, the reference group is "BMI < 24". Standard errors are clustered at the county level and shown in parentheses. Within R^2 represents the proportion of the variation in the dependent variable explained by the independent variables, excluding the contribution of the fixed effects. *p<0.1, **p<0.05, ***p<0.01.

Table S24. The individual characteristics heterogeneous impacts of number of days with EHF>0 on mortality risk of older adults.

Variables	Mortality risk of older adults		
Annual mean temperature index	0.00522***	0.00519***	0.00525***
	[0.00130]	[0.00128]	[0.00125]
Day-to-day temperature index variability	0.03045***	0.03035***	0.02969***
	[0.00972]	[0.00971]	[0.00929]
Annual mean EHF	0.00769***	0.00760***	0.00775***
	[0.00155]	[0.00153]	[0.00155]
Number of days with EHF>0	0.00508***	0.00439***	0.00576***
	[0.00069]	[0.00081]	[0.00064]
Number of days with EHF>0*Sex [Woman]	0.00090***		
	[0.00019]		
Number of days with EHF>0*Age_group [75-85]		0.00023	
		[0.00053]	
Number of days with EHF>0*Age_group [85-95]		0.00105*	
		[0.00059]	
Number of days with EHF>0*Age_group [>85]		0.00216***	
		[0.00071]	
Number of days with EHF>0*BMI [24-28]			-0.00034
			[0.00024]
Number of days with EHF>0*BMI [>28]			0.00044
			[0.00047]
Annual total precipitation	0.00586***	0.00578***	0.00630***
	[0.00135]	[0.00134]	[0.00115]
Province-by-Year FE	YES	YES	YES
Province-by-Month FE	YES	YES	YES
Control variables	YES	YES	YES
Observations	41246	41246	41246
Adjusted R^2	0.724	0.724	0.724
Within R^2	0.0963	0.0962	0.0963

Notes: Each column in the table represents a separate fixed-effects regression with interaction terms based on the specification of Model 5. The numbers show the regression coefficients of each annual temperature metric on mortality risk of older adults. We interact individual characteristics of older adults (sex, age and BMI) with number of days with EHF>0 to assess the heterogeneity effects in number of days with EHF>0 across different individual characteristics. The interaction terms represent the difference in the effects of the number of days with EHF>0 between the subgroup and the reference group. For the sex subgroup, the reference group is "man"; for the age subgroups, the reference group is "65-75 years old"; and for BMI, the reference group is "BMI < 24". Standard errors are clustered at the county level and shown in parentheses. Within R^2 represents the proportion of the variation in the dependent variable explained by the independent variables, excluding the contribution of the fixed effects. *p<0.1, **p<0.05, ***p<0.01.

Table S25. The economic status heterogeneous impacts of annual mean temperature index on mortality risk of older adults.

Variables	Mortality risk of older adults		
Annual mean temperature index	0.00646***	0.00550***	0.00650***
	[0.00126]	[0.00131]	[0.00129]
Annual mean temperature index*Income_group [Low]	-0.00064***		
	[0.00023]		
Annual mean temperature index*Income_group [Middle]	-0.00133***		
	[0.00026]		
Annual mean temperature index*Income_group [High]	-0.00265***		
	[0.00029]		
Annual mean temperature index*Income_group [Highest]	-0.00343***		
	[0.00045]		
Annual mean temperature index*Residence [Urban]		-0.00073***	
		[0.00017]	
Annual mean temperature index*Residence [Rural]*Income_group [Low]			-0.00068**
			[0.00028]
Annual mean temperature index*Residence [Rural]*Income_group [Middle]			-0.00150***
			[0.00031]
Annual mean temperature index*Residence [Rural]*Income_group [High]			-0.00208***
			[0.00031]
Annual mean temperature index*Residence [Rural]*Income_group [Highest]			-0.00203***
			[0.00030]
Annual mean temperature index*Residence [Urban]*Income_group [Lowest]			-0.00055*
			[0.00033]
Annual mean temperature index*Residence [Urban]*Income_group [Low]			-0.00102***
			[0.00031]
Annual mean temperature index*Residence [Urban]*Income_group [Middle]			-0.00141***
			[0.00032]
Annual mean temperature index*Residence [Urban]*Income_group [High]			-0.00305***
			[0.00035]
Annual mean temperature index*Residence [Urban]*Income_group [Highest]			-0.00306***
			[0.00033]
Day-to-day temperature index variability	0.03039***	0.03080***	0.03078***
	[0.00960]	[0.00965]	[0.00958]
Annual mean EHF	0.00762***	0.00766***	0.00761***
	[0.00154]	[0.00155]	[0.00154]
Number of days with EHF>0	0.00542***	0.00547***	0.00545***
	[0.00070]	[0.00069]	[0.00069]
Annual total precipitation	0.00578***	0.00588***	0.00584***
	[0.00137]	[0.00136]	[0.00135]
Province-by-Year FE	YES	YES	YES
Province-by-Month FE	YES	YES	YES
Control variables	YES	YES	YES
Observations	41379	41246	41246
Adjusted R^2	0.725	0.724	0.725
Within R^2	0.0988	0.0967	0.0993

Notes: Each column in the table represents a separate fixed-effects regression with interaction terms based on the specification of Model 5. The numbers show the regression coefficients of each annual temperature metric on mortality risk of older adults. We interact economic status of older adults (household per capita annual income, urban-rural residence, and the interaction term of urban-rural residence with household per capita annual income groups) with annual mean temperature index to assess the heterogeneity effects in annual mean temperature index across different economic status. The interaction terms represent the difference in the effects of the annual mean temperature index between the subgroup and the reference group. For the income subgroups, the reference group is “lowest income”; for the residence subgroup, the reference group is “rural residence”; and for income-residence interaction term, the reference group is “lowest income and rural residence”. Standard errors are clustered at the county level and shown in parentheses. Within R^2 represents the proportion of the variation in the dependent variable explained by the independent variables, excluding the contribution of the fixed effects. *p<0.1, **p<0.05, ***p<0.01.

Table S26. The economic status heterogeneous impacts of day-to-day temperature index variability on mortality risk of older adults.

Variables	Mortality risk of older adults		
Annual mean temperature index	0.00486***	0.00515***	0.00495***
	[0.00129]	[0.00130]	[0.00129]
Day-to-day temperature index variability	0.04135***	0.03340***	0.04159***
	[0.00979]	[0.00965]	[0.00956]
Day-to-day temperature index variability*Income_group [Low]	-0.00485***		
	[0.00150]		
Day-to-day temperature index variability*Income_group [Middle]	-0.00878***		
	[0.00166]		
Day-to-day temperature index variability*Income_group [High]	-0.01734***		
	[0.00192]		
Day-to-day temperature index variability*Income_group [Highest]	-0.02206***		
	[0.00296]		
Day-to-day temperature index variability*Residence [Urban]		-0.00530***	
		[0.00110]	
Day-to-day temperature index variability*Residence [Rural]*Income_group [Low]			-0.00509***
			[0.00187]
Day-to-day temperature index variability*Residence [Rural]*Income_group [Middle]			-0.00946***
			[0.00209]
Day-to-day temperature index variability*Residence [Rural]*Income_group [High]			-0.01392***
			[0.00208]
Day-to-day temperature index variability*Residence [Rural]*Income_group [Highest]			-0.01328***
			[0.00219]
Day-to-day temperature index variability*Residence [Urban]*Income_group [Lowest]			-0.00392*
			[0.00223]
Day-to-day temperature index variability*Residence [Urban]*Income_group [Low]			-0.00775***
			[0.00214]
Day-to-day temperature index variability*Residence [Urban]*Income_group [Middle]			-0.01029***
			[0.00215]
Day-to-day temperature index variability*Residence [Urban]*Income_group [High]			-0.02007***
			[0.00221]
Day-to-day temperature index variability*Residence [Urban]*Income_group [Highest]			-0.02092***
			[0.00218]
Annual mean EHF	0.00761***	0.00767***	0.00761***
	[0.00155]	[0.00155]	[0.00154]
Number of days with EHF>0	0.00542***	0.00546***	0.00544***
	[0.00070]	[0.00069]	[0.00069]
Annual total precipitation	0.00576***	0.00587***	0.00582***
	[0.00136]	[0.00135]	[0.00135]
Province-by-Year FE	YES	YES	YES
Province-by-Month FE	YES	YES	YES
Control variables	YES	YES	YES
Observations	41379	41246	41246
Adjusted R^2	0.725	0.724	0.725
Within R^2	0.0987	0.0968	0.0992

Notes: Each column in the table represents a separate fixed-effects regression with interaction terms based on the specification of Model 5. The numbers show the regression coefficients of each annual temperature metric on mortality risk of older adults. We interact economic status of older adults (household per capita annual income, urban-rural residence, and the interaction term of urban-rural residence with household per capita annual income groups) with day-to-day temperature index variability to assess the heterogeneity effects in day-to-day temperature index variability across different economic status. The interaction terms represent the difference in the effects of the day-to-day temperature index variability between the subgroup and the reference group. For the income subgroups, the reference group is "lowest income"; for the residence subgroup, the reference group is "rural residence"; and for income-residence interaction term, the reference group is "lowest income and rural residence". Standard errors are clustered at the county level and shown in parentheses. Within R^2 represents the proportion of the variation in the dependent variable explained by the independent variables, excluding the contribution of the fixed effects. *p<0.1, **p<0.05, ***p<0.01.

Table S27. The economic status heterogeneous impacts of annual mean EHF on mortality risk of older adults.

Variables	Mortality risk of older adults		
Annual mean temperature index	0.00504***	0.00511***	0.00494***
	[0.00131]	[0.00130]	[0.00131]
Day-to-day temperature index variability	0.02997***	0.03070***	0.02959***
	[0.00969]	[0.00967]	[0.00974]
Annual mean EHF	0.00955***	0.00881***	0.01126***
	[0.00203]	[0.00166]	[0.00202]
Annual mean EHF*Income_group [Low]	-0.00177**		
	[0.00081]		
Annual mean EHF*Income_group [Middle]	-0.00193*		
	[0.00099]		
Annual mean EHF*Income_group [High]	-0.00386***		
	[0.00118]		
Annual mean EHF*Income_group [Highest]	-0.00158		
	[0.00161]		
Annual mean EHF*Residence [Urban]		-0.00252***	
		[0.00075]	
Annual mean EHF*Residence [Rural]*Income_group [Low]			-0.00149
			[0.00101]
Annual mean EHF*Residence [Rural]*Income_group [Middle]			-0.00248**
			[0.00113]
Annual mean EHF*Residence [Rural]*Income_group [High]			-0.00434***
			[0.00138]
Annual mean EHF*Residence [Rural]*Income_group [Highest]			-0.00363***
			[0.00138]
Annual mean EHF*Residence [Urban]*Income_group [Lowest]			-0.00133
			[0.00135]
Annual mean EHF*Residence [Urban]*Income_group [Low]			-0.00420***
			[0.00119]
Annual mean EHF*Residence [Urban]*Income_group [Middle]			-0.00328**
			[0.00141]
Annual mean EHF*Residence [Urban]*Income_group [High]			-0.00682***
			[0.00139]
Annual mean EHF*Residence [Urban]*Income_group [Highest]			-0.00708***
			[0.00144]
Number of days with EHF>0	0.00545***	0.00548***	0.00550***
	[0.00070]	[0.00069]	[0.00069]
Annual total precipitation	0.00581***	0.00586***	0.00585***
	[0.00137]	[0.00136]	[0.00136]
Province-by-Year FE	YES	YES	YES
Province-by-Month FE	YES	YES	YES
Control variables	YES	YES	YES
Observations	41379	41246	41246
Adjusted R^2	0.724	0.724	0.724
Within R^2	0.0968	0.0966	0.0966

Notes: Each column in the table represents a separate fixed-effects regression with interaction terms based on the specification of Model 5. The numbers show the regression coefficients of each annual temperature metric on mortality risk of older adults. We interact economic status of older adults (household per capita annual income, urban-rural residence, and the interaction term of urban-rural residence with household per capita annual income groups) with annual mean EHF to assess the heterogeneity effects in annual mean EHF across different economic status. The interaction terms represent the difference in the effects of the annual mean EHF between the subgroup and the reference group. For the income subgroups, the reference group is "lowest income"; for the residence subgroup, the reference group is "rural residence"; and for income-residence interaction term, the reference group is "lowest income and rural residence". Standard errors are clustered at the county level and shown in parentheses. Within R^2 represents the proportion of the variation in the dependent variable explained by the independent variables, excluding the contribution of the fixed effects. *p<0.1, **p<0.05, ***p<0.01.

Table S28. The economic status heterogeneous impacts of number of days with EHF>0 on mortality risk of older adults.

Variables	Mortality risk of older adults		
Annual mean temperature index	0.00499***	0.00516***	0.00497***
	[0.00130]	[0.00130]	[0.00131]
Day-to-day temperature index variability	0.03039***	0.03046***	0.03061***
	[0.00964]	[0.00968]	[0.00965]
Annual mean EHF	0.00766***	0.00766***	0.00765***
	[0.00154]	[0.00155]	[0.00154]
Number of days with EHF>0	0.00676***	0.00586***	0.00733***
	[0.00073]	[0.00071]	[0.00070]
Number of days with EHF>0*Income_group [Low]	-0.00089***		
	[0.00030]		
Number of days with EHF>0*Income_group [Middle]	-0.00128***		
	[0.00033]		
Number of days with EHF>0*Income_group [High]	-0.00218***		
	[0.00036]		
Number of days with EHF>0*Income_group [Highest]	-0.00197***		
	[0.00058]		
Number of days with EHF>0*Residence [Urban]		-0.00089***	
		[0.00024]	
Number of days with EHF>0*Residence [Rural]*Income_group [Low]			-0.00101***
			[0.00036]
Number of days with EHF>0*Residence [Rural]*Income_group [Middle]			-0.00164***
			[0.00040]
Number of days with EHF>0*Residence [Rural]*Income_group [High]			-0.00229***
			[0.00041]
Number of days with EHF>0*Residence [Rural]*Income_group [Highest]			-0.00240***
			[0.00047]
Number of days with EHF>0*Residence [Urban]*Income_group [Lowest]			-0.00087*
			[0.00045]
Number of days with EHF>0*Residence [Urban]*Income_group [Low]			-0.00164***
			[0.00044]
Number of days with EHF>0*Residence [Urban]*Income_group [Middle]			-0.00179***
			[0.00043]
Number of days with EHF>0*Residence [Urban]*Income_group [High]			-0.00323***
			[0.00043]
Number of days with EHF>0*Residence [Urban]*Income_group [Highest]			-0.00343***
			[0.00041]
Annual total precipitation	0.00578***	0.00584***	0.00579***
	[0.00136]	[0.00135]	[0.00135]
Province-by-Year FE	YES	YES	YES
Province-by-Month FE	YES	YES	YES
Control variables	YES	YES	YES
Observations	41379	41246	41246
Adjusted R^2	0.725	0.724	0.725
Within R^2	0.0974	0.0966	0.0978

Notes: Each column in the table represents a separate fixed-effects regression with interaction terms based on the specification of Model 5. The numbers show the regression coefficients of each annual temperature metric on mortality risk of older adults. We interact economic status of older adults (household per capita annual income, urban-rural residence, and the interaction term of urban-rural residence with household per capita annual income groups) with the number of days with EHF>0 to assess the heterogeneity effects in number of days with EHF>0 across different economic status. The interaction terms represent the difference in the effects of the number of days with EHF>0 between the subgroup and the reference group. For the income subgroups, the reference group is "lowest income"; for the residence subgroup, the reference group is "rural residence"; and for income-residence interaction term, the reference group is "lowest income and rural residence". Standard errors are clustered at the county level and shown in parentheses. Within R^2 represents the proportion of the variation in the dependent variable explained by the independent variables, excluding the contribution of the fixed effects. *p<0.1, **p<0.05, ***p<0.01.

Table S29. The diet heterogeneous impacts of annual mean temperature index on mortality risk of older adults.

Variables	Mortality risk of older adults				
Annual mean temperature index	0.00519***	0.00535***	0.00526***	0.00511***	0.00522***
	[0.00130]	[0.00129]	[0.00130]	[0.00130]	[0.00131]
Annual mean temperature index*Staple food [Coarse grain]	0.00069				
	[0.00043]				
Annual mean temperature index*Pork [Eating everyday]		-0.00036			
		[0.00052]			
Annual mean temperature index*Fish [Eating everyday]			-0.00055		
			[0.00075]		
Annual mean temperature index*Egg [Eating everyday]				0.00026	
				[0.00061]	
Annual mean temperature index*Bean [Eating everyday]					-0.00018
					[0.00071]
Day-to-day temperature index variability	0.03064***	0.03061***	0.03067***	0.03045***	0.03066***
	[0.00968]	[0.00967]	[0.00966]	[0.00971]	[0.00968]
Annual mean EHF	0.00766***	0.00766***	0.00767***	0.00767***	0.00766***
	[0.00155]	[0.00155]	[0.00155]	[0.00155]	[0.00155]
Number of days with EHF>0	0.00546***	0.00546***	0.00546***	0.00546***	0.00546***
	[0.00069]	[0.00069]	[0.00069]	[0.00069]	[0.00069]
Annual total precipitation	0.00588***	0.00586***	0.00587***	0.00586***	0.00587***
	[0.00135]	[0.00135]	[0.00135]	[0.00135]	[0.00135]
Province-by-Year FE	YES	YES	YES	YES	YES
Province-by-Month FE	YES	YES	YES	YES	YES
Control variables	YES	YES	YES	YES	YES
Observations	41246	41246	41246	41246	41246
Adjusted R^2	0.724	0.724	0.724	0.724	0.724
Within R^2	0.0968	0.0968	0.0968	0.0968	0.0968

Notes: Each column in the table represents a separate fixed-effects regression with interaction terms based on the specification of Model 5. The numbers show the regression coefficients of each annual temperature metric on mortality risk of older adults. We interact diet of older adults (staple food category and whether to eat pork/fish/egg/bean every day) with annual mean temperature index to assess the heterogeneity effects in annual mean temperature index across different diet. The interaction terms represent the difference in the effects of the annual mean temperature index between the subgroup and the reference group. For the staple food subgroup, the reference group is “rice and wheat”; and for whether to eat specific foods every day, the reference group is “not eating every day”. Standard errors are clustered at the county level and shown in parentheses. Within R^2 represents the proportion of the variation in the dependent variable explained by the independent variables, excluding the contribution of the fixed effects. *p<0.1, **p<0.05, ***p<0.01.

Table S30. The diet heterogeneous impacts of annual mean temperature index on mortality risk of older adults.

Variables	Mortality risk of older adults				
Annual mean temperature index	0.00528***	0.00499***	0.00527***	0.00474***	0.00503***
	[0.00134]	[0.00134]	[0.00130]	[0.00142]	[0.00132]
Annual mean temperature index*Pickle [Eating everyday]	-0.00045				
	[0.00071]				
Annual mean temperature index*Tea [Eating everyday]		0.00088			
		[0.00061]			
Annual mean temperature index*Garlic [Eating everyday]			-0.00057		
			[0.00060]		
Annual mean temperature index*Vegetables [Eating everyday]				0.00081	
				[0.00066]	
Annual mean temperature index*Fruits [Eating everyday]					0.00151*
					[0.00080]
Day-to-day temperature index variability	0.03054***	0.03035***	0.03076***	0.03041***	0.03041***
	[0.00970]	[0.00968]	[0.00968]	[0.00966]	[0.00968]
Annual mean EHF	0.00767***	0.00767***	0.00767***	0.00768***	0.00768***
	[0.00155]	[0.00155]	[0.00155]	[0.00155]	[0.00155]
Number of days with EHF>0	0.00546***	0.00547***	0.00546***	0.00547***	0.00546***
	[0.00069]	[0.00069]	[0.00069]	[0.00069]	[0.00069]
Annual total precipitation	0.00586***	0.00587***	0.00586***	0.00586***	0.00584***
	[0.00135]	[0.00135]	[0.00135]	[0.00135]	[0.00136]
Province-by-Year FE	YES	YES	YES	YES	YES
Province-by-Month FE	YES	YES	YES	YES	YES
Control variables	YES	YES	YES	YES	YES
Observations	41246	41246	41246	41246	41246
Adjusted R^2	0.724	0.724	0.724	0.724	0.724
Within R^2	0.0968	0.0968	0.0968	0.0968	0.0969

Notes: Each column in the table represents a separate fixed-effects regression with interaction terms based on the specification of Model 5. The numbers show the regression coefficients of each annual temperature metric on mortality risk of older adults. We interact diet of older adults (whether to eat pickle/tea/garlic/vegetables/fruits every day) with annual mean temperature index to assess the heterogeneity effects in annual mean temperature index across different diet. The interaction terms represent the difference in the effects of the annual mean temperature index between the subgroup and the reference group. For whether to eat specific foods every day, the reference group is "not eating every day". Standard errors are clustered at the county level and shown in parentheses. Within R^2 represents the proportion of the variation in the dependent variable explained by the independent variables, excluding the contribution of the fixed effects. *p<0.1, **p<0.05, ***p<0.01.

Table S31. The diet heterogeneous impacts of day-to-day temperature index variability on mortality risk of older adults.

Variables	Mortality risk of older adults				
Annual mean temperature index	0.00517***	0.00518***	0.00521***	0.00519***	0.00512***
	[0.00130]	[0.00130]	[0.00130]	[0.00130]	[0.00130]
Day-to-day temperature index variability	0.03047***	0.03184***	0.03027***	0.03765***	0.03573***
	[0.00969]	[0.00967]	[0.00968]	[0.00960]	[0.00967]
Day-to-day temperature index variability*Staple food [Coarse grain]	0.00147				
	[0.00220]				
Day-to-day temperature index variability*Pork [Eating everyday]		-0.00361			
		[0.00593]			
Day-to-day temperature index variability*Fish [Eating everyday]			0.00165		
			[0.00137]		
Day-to-day temperature index variability*Egg [Eating everyday]				-0.01619***	
				[0.00582]	
Day-to-day temperature index variability*Bean [Eating everyday]					-0.01884***
					[0.00713]
Annual mean EHF	0.00767***	0.00767***	0.00766***	0.00768***	0.00771***
	[0.00155]	[0.00155]	[0.00155]	[0.00155]	[0.00155]
Number of days with EHF>0	0.00546***	0.00546***	0.00547***	0.00545***	0.00544***
	[0.00069]	[0.00069]	[0.00069]	[0.00069]	[0.00069]
Annual total precipitation	0.00586***	0.00587***	0.00587***	0.00586***	0.00584***
	[0.00135]	[0.00135]	[0.00135]	[0.00135]	[0.00135]
Province-by-Year FE	YES	YES	YES	YES	YES
Province-by-Month FE	YES	YES	YES	YES	YES
Control variables	YES	YES	YES	YES	YES
Observations	41246	41246	41246	41246	41246
Adjusted R^2	0.724	0.724	0.724	0.724	0.724
Within R^2	0.0967	0.0968	0.0967	0.0969	0.0969

Notes: Each column in the table represents a separate fixed-effects regression with interaction terms based on the specification of Model 5. The numbers show the regression coefficients of each annual temperature metric on mortality risk of older adults. We interact diet of older adults (staple food category and whether to eat pork/fish/egg/bean every day) with day-to-day temperature index variability to assess the heterogeneity effects in day-to-day temperature index variability across different diet. The interaction terms represent the difference in the effects of the day-to-day temperature index variability between the subgroup and the reference group. For the staple food subgroup, the reference group is "rice and wheat"; and for whether to eat specific foods every day, the reference group is "not eating every day". Standard errors are clustered at the county level and shown in parentheses. Within R^2 represents the proportion of the variation in the dependent variable explained by the independent variables, excluding the contribution of the fixed effects. *p<0.1, **p<0.05, ***p<0.01.

Table S32. The diet heterogeneous impacts of day-to-day temperature index variability on mortality risk of older adults.

Variables	Mortality risk of older adults				
Annual mean temperature index	0.00520***	0.00519***	0.00519***	0.00520***	0.00515***
	[0.00130]	[0.00130]	[0.00130]	[0.00130]	[0.00130]
Day-to-day temperature index variability	0.03060***	0.03006***	0.03120***	0.03073***	0.03251***
	[0.00969]	[0.00986]	[0.00967]	[0.00970]	[0.00977]
Day-to-day temperature index variability*Pickle [Eating everyday]	-0.00014				
	[0.00098]				
Day-to-day temperature index variability*Tea [Eating everyday]		0.00205			
		[0.00631]			
Day-to-day temperature index variability*Garlic [Eating everyday]			-0.00303		
			[0.00630]		
Day-to-day temperature index variability*Vegetables [Eating everyday]				-0.00024	
				[0.00102]	
Day-to-day temperature index variability*Fruits [Eating everyday]					-0.01070
					[0.00835]
Annual mean EHF	0.00767***	0.00767***	0.00766***	0.00767***	0.00766***
	[0.00155]	[0.00155]	[0.00155]	[0.00155]	[0.00155]
Number of days with EHF>0	0.00546***	0.00546***	0.00546***	0.00546***	0.00546***
	[0.00069]	[0.00069]	[0.00069]	[0.00069]	[0.00069]
Annual total precipitation	0.00586***	0.00587***	0.00586***	0.00586***	0.00586***
	[0.00135]	[0.00135]	[0.00135]	[0.00135]	[0.00135]
Province-by-Year FE	YES	YES	YES	YES	YES
Province-by-Month FE	YES	YES	YES	YES	YES
Control variables	YES	YES	YES	YES	YES
Observations	41246	41246	41246	41246	41246
Adjusted R^2	0.724	0.724	0.724	0.724	0.724
Within R^2	0.0968	0.0968	0.0968	0.0967	0.0968

Notes: Each column in the table represents a separate fixed-effects regression with interaction terms based on the specification of Model 5. The numbers show the regression coefficients of each annual temperature metric on mortality risk of older adults. We interact diet of older adults (whether to eat pickle/tea/garlic/vegetables/fruits every day) with day-to-day temperature index variability to assess the heterogeneity effects in day-to-day temperature index variability across different diet. The interaction terms represent the difference in the effects of the day-to-day temperature index variability between the subgroup and the reference group. For whether to eat specific foods every day, the reference group is "not eating every day". Standard errors are clustered at the county level and shown in parentheses. Within R^2 represents the proportion of the variation in the dependent variable explained by the independent variables, excluding the contribution of the fixed effects. *p<0.1, **p<0.05, ***p<0.01.

Table S33. The diet heterogeneous impacts of annual mean EHF on mortality risk of older adults.

Variables	Mortality risk of older adults				
Annual mean temperature index	0.00520***	0.00520***	0.00520***	0.00517***	0.00510***
	[0.00130]	[0.00130]	[0.00130]	[0.00130]	[0.00130]
Day-to-day temperature index variability	0.03070***	0.03059***	0.03091***	0.03059***	0.03074***
	[0.00967]	[0.00968]	[0.00972]	[0.00968]	[0.00964]
Annual mean EHF	0.00773***	0.00796***	0.00819***	0.00812***	0.00835***
	[0.00157]	[0.00168]	[0.00163]	[0.00170]	[0.00170]
Annual mean EHF*Staple food [Coarse grain]	-0.00154				
	[0.00195]				
Annual mean EHF*Pork [Eating everyday]		-0.00099			
		[0.00122]			
Annual mean EHF*Fish [Eating everyday]			-0.00394**		
			[0.00185]		
Annual mean EHF*Egg [Eating everyday]				-0.00101	
				[0.00089]	
Annual mean EHF*Bean [Eating everyday]					-0.00236*
					[0.00125]
Number of days with EHF>0	0.00546***	0.00547***	0.00546***	0.00545***	0.00544***
	[0.00069]	[0.00069]	[0.00069]	[0.00069]	[0.00069]
Annual total precipitation	0.00587***	0.00587***	0.00587***	0.00586***	0.00585***
	[0.00135]	[0.00135]	[0.00135]	[0.00135]	[0.00135]
Province-by-Year FE	YES	YES	YES	YES	YES
Province-by-Month FE	YES	YES	YES	YES	YES
Control variables	YES	YES	YES	YES	YES
Observations	41246	41246	41246	41246	41246
Adjusted R^2	0.724	0.724	0.724	0.724	0.724
Within R^2	0.0968	0.0968	0.0968	0.0968	0.0969

Notes: Each column in the table represents a separate fixed-effects regression with interaction terms based on the specification of Model 5. The numbers show the regression coefficients of each annual temperature metric on mortality risk of older adults. We interact diet of older adults (staple food category and whether to eat pork/fish/egg/bean every day) with annual mean EHF to assess the heterogeneity effects in annual mean EHF across different diet. The interaction terms represent the difference in the effects of the annual mean EHF between the subgroup and the reference group. For the staple food subgroup, the reference group is “rice and wheat”; and for whether to eat specific foods every day, the reference group is “not eating every day”. Standard errors are clustered at the county level and shown in parentheses. Within R^2 represents the proportion of the variation in the dependent variable explained by the independent variables, excluding the contribution of the fixed effects. *p<0.1, **p<0.05, ***p<0.01.

Table S34. The diet heterogeneous impacts of annual mean EHF on mortality risk of older adults.

Variables	Mortality risk of older adults				
Annual mean temperature index	0.00519***	0.00516***	0.00521***	0.00518***	0.00516***
	[0.00129]	[0.00130]	[0.00130]	[0.00129]	[0.00131]
Day-to-day temperature index variability	0.03062***	0.03084***	0.03054***	0.03076***	0.03046***
	[0.00967]	[0.00968]	[0.00968]	[0.00969]	[0.00967]
Annual mean EHF	0.00769***	0.00730***	0.00796***	0.00721***	0.00794***
	[0.00162]	[0.00163]	[0.00163]	[0.00181]	[0.00156]
Annual mean EHF*Pickle [Eating everyday]	-0.00012				
	[0.00144]				
Annual mean EHF*Tea [Eating everyday]		0.00145			
		[0.00106]			
Annual mean EHF*Garlic [Eating everyday]			-0.00154		
			[0.00108]		
Annual mean EHF*Vegetables [Eating everyday]				0.00082	
				[0.00115]	
Annual mean EHF*Fruits [Eating everyday]					-0.00195**
					[0.00085]
Number of days with EHF>0	0.00546***	0.00546***	0.00545***	0.00545***	0.00547***
	[0.00069]	[0.00069]	[0.00069]	[0.00069]	[0.00069]
Annual total precipitation	0.00586***	0.00585***	0.00586***	0.00586***	0.00586***
	[0.00135]	[0.00135]	[0.00135]	[0.00135]	[0.00135]
Province-by-Year FE	YES	YES	YES	YES	YES
Province-by-Month FE	YES	YES	YES	YES	YES
Control variables	YES	YES	YES	YES	YES
Observations	41246	41246	41246	41246	41246
Adjusted R^2	0.724	0.724	0.724	0.724	0.724
Within R^2	0.0968	0.0968	0.0968	0.0968	0.0967

Notes: Each column in the table represents a separate fixed-effects regression with interaction terms based on the specification of Model 5. The numbers show the regression coefficients of each annual temperature metric on mortality risk of older adults. We interact diet of older adults (whether to eat pickle/tea/garlic/vegetables/fruits every day) with annual mean EHF to assess the heterogeneity effects in annual mean EHF across different diet. The interaction terms represent the difference in the effects of the annual mean EHF between the subgroup and the reference group. For whether to eat specific foods every day, the reference group is “not eating every day”. Standard errors are clustered at the county level and shown in parentheses. Within R^2 represents the proportion of the variation in the dependent variable explained by the independent variables, excluding the contribution of the fixed effects. *p<0.1, **p<0.05, ***p<0.01.

Table S35. The diet heterogeneous impacts of number of days with EHF>0 on mortality risk of older adults.

Variables	Mortality risk of older adults				
Annual mean temperature index	0.00517***	0.00519***	0.00519***	0.00520***	0.00519***
	[0.00130]	[0.00130]	[0.00130]	[0.00130]	[0.00130]
Day-to-day temperature index variability	0.03064***	0.03061***	0.03058***	0.03071***	0.03050***
	[0.00968]	[0.00968]	[0.00969]	[0.00968]	[0.00968]
Annual mean EHF	0.00766***	0.00767***	0.00767***	0.00767***	0.00766***
	[0.00155]	[0.00155]	[0.00155]	[0.00155]	[0.00155]
Number of days with EHF>0	0.00543***	0.00544***	0.00547***	0.00524***	0.00556***
	[0.00070]	[0.00074]	[0.00071]	[0.00075]	[0.00072]
Number of days with EHF>0*Staple food [Coarse grain]	0.00111				
	[0.00104]				
Number of days with EHF>0*Pork [Eating everyday]		0.00008			
		[0.00046]			
Number of days with EHF>0*Fish [Eating everyday]			-0.00008		
			[0.00057]		
Number of days with EHF>0*Egg [Eating everyday]				0.00062	
				[0.00046]	
Number of days with EHF>0*Bean [Eating everyday]					-0.00044
					[0.00055]
Annual total precipitation	0.00586***	0.00587***	0.00586***	0.00587***	0.00586***
	[0.00135]	[0.00135]	[0.00135]	[0.00135]	[0.00135]
Province-by-Year FE	YES	YES	YES	YES	YES
Province-by-Month FE	YES	YES	YES	YES	YES
Control variables	YES	YES	YES	YES	YES
Observations	41246	41246	41246	41246	41246
Adjusted R^2	0.724	0.724	0.724	0.724	0.724
Within R^2	0.0968	0.0968	0.0968	0.0968	0.0968

Notes: Each column in the table represents a separate fixed-effects regression with interaction terms based on the specification of Model 5. The numbers show the regression coefficients of each annual temperature metric on mortality risk of older adults. We interact diet of older adults (staple food category and whether to eat pork/fish/egg/bean every day) with number of days with EHF>0 to assess the heterogeneity effects in number of days with EHF>0 across different diet. The interaction terms represent the difference in the effects of the number of days with EHF>0 between the subgroup and the reference group. For the staple food subgroup, the reference group is "rice and wheat"; and for whether to eat specific foods every day, the reference group is "not eating every day". Standard errors are clustered at the county level and shown in parentheses. Within R^2 represents the proportion of the variation in the dependent variable explained by the independent variables, excluding the contribution of the fixed effects. *p<0.1, **p<0.05, ***p<0.01.

Table S36. The diet heterogeneous impacts of number of days with EHF>0 on mortality risk of older adults.

Variables	Mortality risk of older adults				
Annual mean temperature index	0.00517***	0.00519***	0.00520***	0.00519***	0.00519***
	[0.00130]	[0.00130]	[0.00130]	[0.00130]	[0.00130]
Day-to-day temperature index variability	0.03064***	0.03060***	0.03061***	0.03061***	0.03042***
	[0.00968]	[0.00968]	[0.00968]	[0.00968]	[0.00968]
Annual mean EHF	0.00769***	0.00767***	0.00766***	0.00767***	0.00767***
	[0.00155]	[0.00155]	[0.00154]	[0.00155]	[0.00155]
Number of days with EHF>0	0.00562***	0.00543***	0.00553***	0.00547***	0.00555***
	[0.00072]	[0.00073]	[0.00070]	[0.00072]	[0.00068]
Number of days with EHF>0*Pickle [Eating everyday]	-0.00090				
	[0.00059]				
Number of days with EHF>0*Tea [Eating everyday]		0.00014			
		[0.00049]			
Number of days with EHF>0*Garlic [Eating everyday]			-0.00036		
			[0.00054]		
Number of days with EHF>0*Vegetables [Eating everyday]				-0.00001	
				[0.00046]	
Number of days with EHF>0*Fruits [Eating everyday]					-0.00075
					[0.00062]
Annual total precipitation	0.00587***	0.00587***	0.00586***	0.00586***	0.00586***
	[0.00135]	[0.00135]	[0.00135]	[0.00135]	[0.00135]
Province-by-Year FE	YES	YES	YES	YES	YES
Province-by-Month FE	YES	YES	YES	YES	YES
Control variables	YES	YES	YES	YES	YES
Observations	41246	41246	41246	41246	41246
Adjusted R^2	0.724	0.724	0.724	0.724	0.724
Within R^2	0.0968	0.0968	0.0968	0.0968	0.0968

Notes: Each column in the table represents a separate fixed-effects regression with interaction terms based on the specification of Model 5. The numbers show the regression coefficients of each annual temperature metric on mortality risk of older adults. We interact diet of older adults (whether to eat pickle/tea/garlic/vegetables/fruits every day) with number of days with EHF>0 to assess the heterogeneity effects in number of days with EHF>0 across different diet. The interaction terms represent the difference in the effects of the number of days with EHF>0 between the subgroup and the reference group. For whether to eat specific foods every day, the reference group is "not eating every day". Standard errors are clustered at the county level and shown in parentheses. Within R^2 represents the proportion of the variation in the dependent variable explained by the independent variables, excluding the contribution of the fixed effects. *p<0.1, **p<0.05, ***p<0.01.

REFERENCES AND NOTES

1. E. M. Fischer, S. Sippel, R. Knutti, Increasing probability of record-shattering climate extremes. *Nat. Clim. Chang.* **11**, 689–695 (2021).

2. N. Watts, M. Amann, N. Arnell, S. Ayeb-Karlsson, K. Belesova, M. Boykoff, H. Montgomery, The 2019 report of The *Lancet* Countdown on health and climate change: Ensuring that the health of a child born today is not defined by a changing climate. *Lancet* **394**, 1836–1878 (2019).

3. S. Mukherjee, A. K. Mishra, M. E. Mann, C. Raymond, Anthropogenic warming and population growth may double US heat stress by the late 21st century. *Earth Future* **9**, e2020EF001886 (2021).

4. G. A. Meehl, C. Tebaldi, More intense, more frequent, and longer lasting heat waves in the 21st century. *Science* **305**, 994–997 (2004).

5. E. L. Rezende, F. Bozinovic, A. Szilágyi, M. Santos, Predicting temperature mortality and selection in natural *Drosophila* populations. *Science* **369**, 1242–1245 (2020).

6. Change, IPCC Climate, The physical science basis (2013).

7. L. Alexander, S. Perkins, Debate heating up over changes in climate variability. *Environ. Res. Lett.* **8**, 041001 (2013).

8. C. Huntingford, P. D. Jones, V. N. Livina, T. M. Lenton, P. M. Cox, No increase in global temperature variability despite changing regional patterns. *Nature* **500**, 327–330 (2013).

9. L. Shi, I. Kloog, A. Zanobetti, P. Liu, J. D. Schwartz, Impacts of temperature and its variability on mortality in New England. *Nat. Clim. Chang.* **5**, 988–991 (2015).

10. Y. Guo, A. Gasparrini, B. Armstrong, S. Li, B. Tawatsupa, A. Tobias, E. Lavigne, M. de Sousa Zanotti Stagliorio Coelho, M. Leone, X. Pan, S. Tong, L. Tian, H. Kim, M. Hashizume, Y. Honda, Y.-L. L. Guo, C.-F. Wu, K. Punnasiri, S.-M. Yi, P. Michelozzi, P. H. N. Saldiva, G. Williams, Global variation in the effects of ambient temperature on mortality: A systematic evaluation. *Epidemiology* **25**, 781–789 (2014).

11. K. G. Burkart, M. Brauer, A. Y. Aravkin, W. W. Godwin, S. I. Hay, J. He, V. C. Iannucci, S. L. Larson, S. S. Lim, J. Liu, C. J. L. Murray, P. Zheng, M. Zhou, J. D. Stanaway, Estimating the cause-specific relative risks of non-optimal temperature on daily mortality: A two-part modelling approach applied to the Global Burden of Disease Study. *Lancet* **398**, 685–697 (2021).

12. È. Martínez-Solanas, M. Quijal-Zamorano, H. Achebak, D. Petrova, J.-M. Robine, F. R. Herrmann, X. Rodó, J. Ballester, Projections of temperature-attributable mortality in Europe: A time series analysis of 147 contiguous regions in 16 countries. *Lancet Planet. Health* **5**, e446–e454 (2021).

13. J. L. Kephart, B. N. Sánchez, J. Moore, L. H. Schinasi, M. Bakhtsiyarava, Y. Ju, N. Gouveia, W. T. Caiaffa, I. Dronova, S. Arunachalam, A. V. D. Roux, D. A. Rodríguez, City-level impact of extreme temperatures and mortality in Latin America. *Nat. Med.* **28**, 1700–1705 (2022).

14. R. Chen, P. Yin, L. Wang, C. Liu, Y. Niu, W. Wang, Y. Jiang, Y. Liu, J. Liu, J. Qi, J. You, H. Kan, M. Zhou, Association between ambient temperature and mortality risk and burden: Time series study in 272 main Chinese cities. *BMJ* **363**, k4306 (2018).

15. A. Gasparrini, Y. Guo, M. Hashizume, E. Lavigne, A. Zanobetti, J. Schwartz, A. Tobias, S. Tong, J. Rocklöv, B. Forsberg, M. Leone, M. De Sario, M. L. Bell, Y.-L. L. Guo, C.-f. Wu, H. Kan, S.-M. Yi, M. de Sousa Zanotti Stagliorio Coelho, Paulo Hilario Nascimento Saldiva, Y. Honda, H. Kim, B. Armstrong, Mortality risk attributable to high and low ambient temperature: A multicountry observational study. *Lancet* **386**, 369–375 (2015).

16. W. Ma, R. Chen, H. Kan, Temperature-related mortality in 17 large Chinese cities: How heat and cold affect mortality in China. *Environ. Res.* **134**, 127–133 (2014).

17. M. Burke, F. González, P. Baylis, S. Heft-Neal, C. Baysan, S. Basu, S. Hsiang, Higher temperatures increase suicide rates in the United States and Mexico. *Nat. Clim. Chang.* **8**, 723–729 (2018).

18. Q. Yin, J. Wang, Z. Ren, J. Li, Y. Guo, Mapping the increased minimum mortality temperatures in the context of global climate change. *Nat. Commun.* **10**, 4640 (2019).

19. Z. Xu, G. FitzGerald, Y. Guo, B. Jalaludin, S. Tong, Impact of heatwave on mortality under different heatwave definitions: A systematic review and meta-analysis. *Environ. Int.* **89–90**, 193–203 (2016).

20. C. He, H. Kim, M. Hashizume, W. Lee, Y. Honda, S. E. Kim, P. L. Kinney, A. Schneider, Y. Zhang, Y. Zhu, L. Zhou, R. Chen, H. Kan, The effects of night-time warming on mortality burden under future climate change scenarios: A modelling study. *Lancet Planet. Health* **6**, e648–e657 (2022).

21. N. Langlois, J. Herbst, K. Mason, J. Nairn, R. W. Byard, Using the excess heat factor (EHF) to predict the risk of heat related deaths. *J. Forensic Leg. Med.* **20**, 408–411 (2013).

22. G. Hatvani-Kovacs, M. Belusko, J. Pockett, J. Boland, Can the excess heat factor indicate heatwave-related morbidity? A case study in Adelaide, South Australia. *Ecohealth* **13**, 100–110 (2016).

23. J. Nairn, B. Ostendorf, P. Bi, Performance of excess heat factor severity as a global heatwave health impact index. *Int. J. Environ. Res. Public Health* **15**, 2494 (2018).

24. S. Ai, H. Lu, H. Liu, J. Cao, F. Li, X. Qiu, J. Gong, T. Xue, T. Zhu, All-cause mortality attributable to long-term changes in mean temperature and diurnal temperature variation in China: A nationwide quasi-experimental study. *Environ. Res. Lett.* **19**, 014002 (2024).

25. J. A. Patz, D. Engelberg, J. Last, The effects of changing weather on public health. *Annu. Rev. Public Health* **21**, 271–307 (2000).

26. S. C. Sheridan, C. C. Lee, M. J. Allen, The mortality response to absolute and relative temperature extremes. *Int. J. Environ. Res. Public Health* **16**, 1493 (2019).

27. L. Madaniyazi, Y. Chung, Y. Kim, A. Tobias, C. F. S. Ng, X. Seposo, Y. Guo, Y. Honda, A. Gasparrini, B. Armstrong, M. Hashizume, Seasonality of mortality under a changing climate: A time-series analysis of mortality in Japan between 1972 and 2015. *Environ. Health Prev. Med.* **26**, 69 (2021).

28. A. Bunker, J. Wildenhain, A. Vandenbergh, N. Henschke, J. Rocklöv, S. Hajat, R. Sauerborn, Effects of air temperature on climate-sensitive mortality and morbidity outcomes in the elderly; a systematic review and meta-analysis of epidemiological evidence. *EBioMedicine* **6**, 258–268 (2016).

29. K. L. Ebi, A. Capon, P. Berry, C. Broderick, R. de Dear, G. Havenith, Y. Honda, R. S. Kovats, W. Ma, A. Malik, N. B. Morris, L. Nybo, S. I. Seneviratne, J. Vanos, O. Jay, Hot weather and heat extremes: Health risks. *Lancet* **398**, 698–708 (2021).

30. Z. Ren, "China aging research report 2022." http://caoss.org.cn/UploadFile/pic/20229281791192316.pdf.

31. M. Tatum, China's population peak. *Lancet* **399**, 509 (2022).

32. W. Cai, C. Zhang, S. Zhang, Y. Bai, M. Callaghan, N. Chang, B. Chen, H. Chen, L. Cheng, X. Cui, H. Dai, B. Danna, W. Dong, W. Fan, X. Fang, T. Gao, Y. Geng, D. Guan, Y. Hu, J. Hua, C. Huang, H. Huang, J. Huang, L. Jiang, Q. Jiang, X. Jiang, H. Jin, G. Kiesewetter, L. Liang, B. Lin, H. Lin, H. Liu, Q. Liu, T. Liu, X. Liu, X. Liu, Z. Liu, Z. Liu, S. Lou, C. Lu, Z. Luo, W. Meng, H. Miao, C. Ren, M. Romanello, W. Schöpp, J. Su, X. Tang, C. Wang, Q. Wang, L. Warnecke, S. Wen, W. Winiwarter, Y. Xie, B. Xu, Y. Yan, X. Yang, F. Yao, L. Yu, J. Yuan, Y. Zeng, J. Zhang, L. Zhang, R. Zhang, S. Zhang, S. Zhang, Q. Zhao, D. Zheng, H. Zhou, J. Zhou, M. F.-C. C. Fung, Y. Luo, P. Gong, The 2022 China report of the Lancet Countdown on health and climate change: Leveraging climate actions for healthy ageing. *Lancet Public Health* **7**, e1073–e1090 (2022).

33. W. Cai, C. Zhang, S. Zhang, S. Ai, Y. Bai, J. Bao, B. Chen, N. Chang, H. Chen, L. Cheng, X. Cui, H. Dai, B. Danna, Q. Di, W. Dong, W. Dong, D. Dou, W. Fan, X. Fan, X. Fang, Y. Gao, T. Gao, Y. Geng, D. Guan, Y. Guo, Y. Hu, J. Hua, C. Huang, H. Huang, J. Huang, I. Hamilton, Q. Jiang, X. Jiang, P. Ke, G. Kiesewetter, P. Lampard, C. Li, R. Li, S. Li, L. Liang, B. Lin, H. Lin, H. Liu, Q. Liu, X. Liu, Y. Liu, Z. Liu, Z. Liu, X. Liu, S. Lou, C. Lu, Y. Luo, Z. Luo, W. Ma, A. M. Gushin, Y. Niu, C. Ren, Z. Ruan, W. Schöpp, Y. Shan, J. Su, T. Sun, Q. Wang, C. Wang, S. Wen, Y. Xie, H. Xiong, B. Xu, M. Xu, Y. Yan, J. Yang, L. Yang, X. Yang, L. Yu, Y. Yue, Y. Zeng, Y. Zhang, S. Zhang, Z. Zhang, J. Zhang, L. Zhao, Q. Zhao, Z. Zhao, J. Zhao, M. Zhao, J.

Zhou, Z. Zhu, M. C. F. Fu-Chun, P. Gong, The 2021 China report of the Lancet Countdown on health and climate change: Seizing the window of opportunity. *Lancet Public Health* **6**, e932–e947 (2021).

34. X. Ye, R. Wolff, W. Yu, P. Vaneckova, X. Pan, S. Tong, Ambient temperature and morbidity: A review of epidemiological evidence. *Environ. Health Perspect.* **120**, 19–28 (2012).

35. T. Liu, C. Zhou, H. Zhang, B. Huang, Y. Xu, L. Lin, L. Wang, R. Hu, Z. Hou, Y. Xiao, J. Li, X. Xu, D. Jin, M. Qin, Q. Zhao, W. Gong, P. Yin, Y. Xu, J. Hu, J. Xiao, W. Zeng, X. Li, S. Chen, L. Guo, Z. Rong, Y. Zhang, C. Huang, Y. Du, Y. Guo, S. Rutherford, M. Yu, M. Zhou, W. Ma, Ambient temperature and years of life lost: A national study in China. *Innovation* **2**, 100072 (2021).

36. J. R. Buzan, M. Huber, Moist heat stress on a hotter Earth. *Annu. Rev. Earth Planet. Sci.* **48**, 623–655 (2020).

37. S. Heft-Neal, J. Burney, E. Bendavid, M. Burke, Robust relationship between air quality and infant mortality in Africa. *Nature* **559**, 254–258 (2018).

38. A. Terriau, J. Albertini, E. Montassier, A. Poirier, Q. Le Bastard, Estimating the impact of virus testing strategies on the COVID-19 case fatality rate using fixed-effects models. *Sci. Rep.* **11**, 21650 (2021).

39. K. Grace, Considering climate in studies of fertility and reproductive health in poor countries. *Nat. Clim. Chang.* **7**, 479–485 (2017).

40. W. C. Willett, M. J. Stampfer, Current evidence on healthy eating. *Annu. Rev. Public Health* **34**, 77–95 (2013).

41. S. B. Seidelmann, B. Claggett, S. Cheng, M. Henglin, A. Shah, L. M. Steffen, A. R. Folsom, E. B. Rimm, W. C. Willett, S. D. Solomon, Dietary carbohydrate intake and mortality: A prospective cohort study and meta-analysis. *Lancet Public Health* **3**, e419–e428 (2018).

42. GBD 2017 Diet Collaborators, Health effects of dietary risks in 195 countries, 1990–2017: A systematic analysis for the Global Burden of Disease Study 2017. *Lancet* **393**, 1958–1972 (2019).

43. S. E. Perkins-Kirkpatrick, S. C. Lewis, Increasing trends in regional heatwaves. *Nat. Commun.* **11**, 3357 (2020).

44. C. Tuholske, K. Caylor, C. Funk, A. Verdin, S. Sweeney, K. Grace, P. Peterson, T. Evans, Global urban population exposure to extreme heat. *Proc. Natl. Acad. Sci. U.S.A.* **118**, e2024792118 (2021).

45. C. B. Field, V. R. Barros, Eds., *Climate change 2014—Impacts, Adaptation and Vulnerability: Regional Aspects* (Cambridge Univ. Press, 2014).

46. H. Green, J. Bailey, L. Schwarz, J. Vanos, K. Ebi, T. Benmarhnia, Impact of heat on mortality and morbidity in low and middle income countries: A review of the epidemiological evidence and considerations for future research. *Environ. Res.* **171**, 80–91 (2019).

47. P. F. D. Scheelbeek, A. D. Dangour, S. Jarmul, G. Turner, A. J. Sietsma, J. C. Minx, M. Callaghan, I. Ajibade, S. E. Austin, R. Biesbroek, K. J. Bowen, T. Chen, K. Davis, T. Ensor, J. D. Ford, E. K. Galappaththi, E. T. Joe, I. J. Musah-Surugu, G. N. Alverio, P. N. Schwerdtle, P. Pokharel, E. A. Salubi, G. Scarpa, A. C. Segnon, M. Siña, S. Templeman, J. Xu, C. Zavaleta-Cortijo, L. Berrang-Ford, The effects on public health of climate change adaptation responses: A systematic review of evidence from low-and middle-income countries. *Environ. Res. Lett.* **16**, 073001 (2021).

48. O. Jay, A. Capon, P. Berry, C. Broderick, R. de Dear, G. Havenith, Y. Honda, R. S. Kovats, W. Ma, A. Malik, N. B. Morris, L. Nybo, S. I. Seneviratne, J. Vanos, K. L. Ebi, Reducing the health effects of hot weather and heat extremes: From personal cooling strategies to green cities. *Lancet* **398**, 709–724 (2021).

49. N. Watts, W. N. Adger, P. Agnolucci, J. Blackstock, P. Byass, W. Cai, S. Chaytor, T. Colbourn, M. Collins, A. Cooper, P. M. Cox, J. Depledge, P. Drummond, P. Ekins, V. Galaz, D. Grace, H.

Graham, M. Grubb, A. Haines, I. Hamilton, A. Hunter, X. Jiang, M. Li, I. Kelman, L. Liang, M. Lott, R. Lowe, Y. Luo, G. Mace, M. Maslin, M. Nilsson, T. Oreszczyn, S. Pye, T. Quinn, M. Svensdotter, S. Venevsky, K. Warner, B. Xu, J. Yang, Y. Yin, C. Yu, Q. Zhang, P. Gong, H. Montgomery, A. Costello, Health and climate change: Policy responses to protect public health. *Lancet* **386**, 1861–1914 (2015).

50. B. K. Kennedy, S. L. Berger, A. Brunet, J. Campisi, A. M. Cuervo, E. S. Epel, C. Franceschi, G. J. Lithgow, R. I. Morimoto, J. E. Pessin, T. A. Rando, A. Richardson, E. E. Schadt, T. Wyss-Coray, F. Sierra, Geroscience: Linking aging to chronic disease. *Cell* **159**, 709–713 (2014).

51. US Burden of Disease Collaborators, A. H. Mokdad, K. Ballestros, M. Echko, S. Glenn, H. E. Olsen, E. Mullany, A. Lee, A. R. Khan, A. Ahmadi, A. J. Ferrari, A. Kasaeian, A. Werdecker, A. Carter, B. Zipkin, B. Sartorius, B. Serdar, B. L. Sykes, C. Troeger, C. Fitzmaurice, C. D. Rehm, D. Santomauro, D. Kim, D. Colombara, D. C. Schwebel, D. Tsoi, D. Kolte, E. Nsoesie, E. Nichols, E. Oren, F. J. Charlson, G. C. Patton, G. A. Roth, H. D. Hosgood, H. A. Whiteford, H. Kyu, H. E. Erskine, H. Huang, I. Martopullo, J. A. Singh, J. B. Nachega, J. R. Sanabria, K. Abbas, K. Ong, K. Tabb, K. J. Krohn, L. Cornaby, L. Degenhardt, M. Moses, M. Farvid, M. Griswold, M. Criqui, M. Bell, M. Nguyen, M. Wallin, M. Mirarefin, M. Qorbani, M. Younis, N. Fullman, P. Liu, P. Briant, P. Gona, R. Havmoller, R. Leung, R. Kimokoti, S. Bazargan-Hejazi, S. I. Hay, S. Yadgir, S. Biryukov, S. E. Vollset, T. Alam, T. Frank, T. Farid, T. Miller, T. Vos, T. Bärnighausen, T. T. Gebrehiwot, Y. Yano, Z. Al-Aly, A. Mehari, A. Handal, A. Kandel, B. Anderson, B. Biroscak, D. Mozaffarian, E. R. Dorsey, E. L. Ding, E.-K. Park, G. Wagner, G. Hu, H. Chen, J. E. Sunshine, J. Khubchandani, J. Leasher, J. Leung, J. Salomon, J. Unutzer, L. Cahill, L. Cooper, M. Horino, M. Brauer, N. Breitborde, P. Hotez, R. Topor-Madry, S. Soneji, S. Stranges, S. James, S. Amrock, S. Jayaraman, T. Patel, T. Akinyemiju, V. Skirbekk, Y. Kinfu, Z. Bhutta, J. B. Jonas, C. J. L. Murray, The state of US health, 1990-2016: Burden of diseases, injuries, and risk factors among US states. *JAMA* **319**, 1444–1472 (2018).

52. G. P. Kenny, O. Jay, Thermometry, calorimetry, and mean body temperature during heat stress. *Compr. Physiol.* **3**, 1689–1719 (2013).

53. L. B. Rowell, Cardiovascular aspects of human thermoregulation. *Circ. Res.* **52**, 367–379 (1983).

54. W. C. Adams, R. H. Fox, A. J. Fry, I. C. MacDonald, Thermoregulation during marathon running in cool, moderate, and hot environments. *J. Appl. Physiol.* **38**, 1030–1037 (1975).

55. W. J. Welch, Mammalian stress response: Cell physiology, structure/function of stress proteins, and implications for medicine and disease. *Physiol. Rev.* **72**, 1063–1081 (1992).

56. A. D. Flouris, P. C. Dinas, L. G. Ioannou, L. Nybo, G. Havenith, G. P. Kenny, T. Kjellstrom, Workers' health and productivity under occupational heat strain: A systematic review and meta-analysis. *Lancet Planet. Health* **2**, e521–e531 (2018).

57. R. Khosla, N. D. Miranda, P. A. Trotter, A. Mazzone, R. Renaldi, C. M. Elroy, F. Cohen, A. Jani, R. Perera-Salazar, M. M. Culloch, Cooling for sustainable development. *Nat. Sustain.* **4**, 201–208 (2021).

58. X. Peng, China’s demographic history and future challenges. *Science* **333**, 581–587 (2011).

59. Copernicus Climate Change Service, ECMWF Reanalysis v5 (ERA5). https://ecmwf.int/en/forecasts/dataset/ecmwf-reanalysis-v5.

60. EartH2Observe, L.S., WFDEI and ERA-interim data merged and bias-corrected for ISIMIP (EWEMBI). V. 1.1. GFZ Data Services. (2019). https://isimip.org/gettingstarted/input-data-bias-adjustment/details/27.

61. G. B. Anderson, M. L. Bell, Heat waves in the United States: Mortality risk during heat waves and effect modification by heat wave characteristics in 43 U.S. communities. *Environ. Health Perspect.* **119**, 210–218 (2011).

62. O. A. Alduchov, R. E. Eskridge, Improved Magnus form approximation of saturation vapor pressure. *J. Appl. Meteorol.* **35**, 601–609 (1996).

63. M. Kotz, L. Wenz, A. Stechemesser, M. Kalkuhl, A. Levermann, Day-to-day temperature variability reduces economic growth. *Nat. Clim. Chang.* **11**, 319–325 (2021).

64. J. Nairn, R. Fawcett, D. Ray, “Defining and predicting excessive heat events, a national system” in *Modelling and Understanding High Impact Weather: Extended Abstracts of the Third CAWCR Modelling Workshop* (2009), pp. 83–86, vol. 30.

65. J. D. Angrist, J. S. Pischke, *Mostly Harmless Econometrics: An Empiricist's Companion* (Princeton Univ. Press, 2009).

66. J. M. Wooldridge, *Econometric Analysis of Cross Section and Panel Data* (MIT Press, 2010).

67. B. F. Zhou, Predictive values of body mass index and waist circumference for risk factors of certain related diseases in Chinese adults--study on optimal cut-off points of body mass index and waist circumference in Chinese adults. *Biomed. Environ. Sci.* **15**, 83–96 (2002).

68. L. S. Aiken, *Multiple Regression: Testing and Interpreting Interactions* (Sage, 1991).

69. H. Aguinis, *Regression Analysis for Categorical Moderators* (Guilford Press, 2004).

70. T. A. Carte, C. J. Russell, In pursuit of moderation: Nine common errors and their solutions. *MIS Q.* **27**, 479–501 (2003).

71. M. Kotz, A. Levermann, L. Wenz, The effect of rainfall changes on economic production. *Nature* **601**, 223–227 (2022).

72. L. Li, A. McMurray, X. Li, Y. Gao, J. Xue, The diminishing marginal effect of R&D input and carbon emission mitigation. *J. Clean. Prod.* **282**, 124423 (2021).

73. R. G. Steadman, The assessment of sultriness. Part I: A temperature-humidity index based on human physiology and clothing science. *J. Appl. Meteorol. Climatol.* **18**, 861–873 (1979).

74. R. G. Steadman, The assessment of sultriness. Part II: Effects of wind, extra radiation and barometric pressure on apparent temperature. *J. Appl. Meteorol.* **18**, 874–885 (1979).

75. L. P. Rothfusz, The heat index equation (or, more than you ever wanted to know about heat index) (NWS Southern Region Headquarters, Fort Worth, Texas: National Oceanic and Atmospheric Administration, National Weather Service, Office of Meteorology 9023, 640, 1990).

76. T. A. Carleton, S. M. Hsiang, Social and economic impacts of climate. *Science* **353**, 6304 (2016).

77. M. Auffhammer, S. M. Hsiang, W. Schlenker, A. Sobel, Using weather data and climate model output in economic analyses of climate change. *Rev. Environ. Econ. Policy* **7**, 181–198 (2013).

78. C. D. Kolstad, F. C. Moore, Estimating the economic impacts of climate change using weather observations. *Rev. Environ. Econ. Policy* **14**, 1–24 (2020).

79. D. A. Freedman, On regression adjustments in experiments with several treatments. *Ann. Appl. Stat.* **2**, 176–196 (2008).

80. O. Hellevik, Linear versus logistic regression when the dependent variable is a dichotomy. *Qual. Quant.* **43**, 59–74 (2009).

81. W. C. Horrace, R. L. Oaxaca, Results on the bias and inconsistency of ordinary least squares for the linear probability model. *Econ. Lett.* **90**, 321–327 (2006).

82. C. J. Muller, R. F. MacLehose, Estimating predicted probabilities from logistic regression: Different methods correspond to different target populations. *Int. J. Epidemiol.* **43**, 962–970 (2014).

83. N. Beck, Estimating grouped data models with a binary-dependent variable and fixed effects via a logit versus a linear probability model: The impact of dropped units. *Political Anal.* **28**, 139–145 (2020).

84. E. B. Andersen, *Conditional Inference and Models for Measuring* (Mentalhygiejnisk Forlag, 1973).

85. H. Cheng, “Logit and probit models” in *The Econometrics of Panel Data: Handbook of Theory and Applications* (Springer, 1992), pp. 223–241.

86. A. Ritschl, *Spurious Significance of Treatment Effects in Overfitted Fixed Effect Models* (London School of Economics, 2009).

87. B. H. Baltagi, S. H. Song, Unbalanced panel data: A survey. *Stat. Pap.* **47**, 493–523 (2006).

88. M. Verbeek, T. Nijman, Testing for selectivity bias in panel data models. *Int. Econ. Rev.* **33**, 681–703 (1992).

89. J. M. Brick, Unit nonresponse and weighting adjustments: A critical review. *J. Off. Stat.* **29**, 329–353 (2013).

90. A. Abadie, S. Athey, G. W. Imbens, J. M. Wooldridge, When should you adjust standard errors for clustering? *Q. J. Econ.* **138**, 1–35 (2023).

91. J. G. MacKinnon, M. Ø. Nielsen, M. D. Webb, Cluster-robust inference: A guide to empirical practice. *J. Econ.* **232**, 272–299 (2023).
